# Functional coordination of alternative splicing in the mammalian central nervous system

**DOI:** 10.1186/gb-2007-8-6-r108

**Published:** 2007-06-12

**Authors:** Matthew Fagnani, Yoseph Barash, Joanna Y Ip, Christine Misquitta, Qun Pan, Arneet L Saltzman, Ofer Shai, Leo Lee, Aviad Rozenhek, Naveed Mohammad, Sandrine Willaime-Morawek, Tomas Babak, Wen Zhang, Timothy R Hughes, Derek van der Kooy, Brendan J Frey, Benjamin J Blencowe

**Affiliations:** 1 Banting and Best Department of Medical Research, Centre for Cellular and Biomolecular Research, University of Toronto, 160 College Street, Toronto, Ontario, Canada. M5S 3E1; 2 Department of Molecular and Medical Genetics, Centre for Cellular and Biomolecular Research, University of Toronto, 160 College Street, Toronto, Ontario, Canada. M5S 3E1; 3 Department of Electrical and Computer Engineering, University of Toronto, 40 St. George's Street, Toronto, Ontario, Canada; 4 School of Computer Science and Engineering, Hebrew University, Jerusalem 91904, Israel

## Abstract

**Background:**

Alternative splicing (AS) functions to expand proteomic complexity and plays numerous important roles in gene regulation. However, the extent to which AS coordinates functions in a cell and tissue type specific manner is not known. Moreover, the sequence code that underlies cell and tissue type specific regulation of AS is poorly understood.

**Results:**

Using quantitative AS microarray profiling, we have identified a large number of widely expressed mouse genes that contain single or coordinated pairs of alternative exons that are spliced in a tissue regulated fashion. The majority of these AS events display differential regulation in central nervous system (CNS) tissues. Approximately half of the corresponding genes have neural specific functions and operate in common processes and interconnected pathways. Differential regulation of AS in the CNS tissues correlates strongly with a set of mostly new motifs that are predominantly located in the intron and constitutive exon sequences neighboring CNS-regulated alternative exons. Different subsets of these motifs are correlated with either increased inclusion or increased exclusion of alternative exons in CNS tissues, relative to the other profiled tissues.

**Conclusion:**

Our findings provide new evidence that specific cellular processes in the mammalian CNS are coordinated at the level of AS, and that a complex splicing code underlies CNS specific AS regulation. This code appears to comprise many new motifs, some of which are located in the constitutive exons neighboring regulated alternative exons. These data provide a basis for understanding the molecular mechanisms by which the tissue specific functions of widely expressed genes are coordinated at the level of AS.

## Abstract 2

A microarray analysis provides new evidence suggesting that specific cellular processes in the mammalian CNS are coordinated at the level of alternative splicing, and that a complex splicing code underlies CNS-specific alternative splicing regulation.

## Background

Alternative splicing (AS) is the process by which the exon sequences of primary transcripts are differentially included in mature mRNA, and it represents an important mechanism underlying the regulation and diversification of gene function [1-4]. Comparisons of data from transcript sequencing efforts and microarray profiling experiments have provided evidence that AS is more frequent in organisms with increased cellular and functional specialization [4-6]. It is estimated that more than 66% of mouse and human genes contain one or more alternative exons [7]. Moreover, transcripts expressed in organs consisting of large numbers of specialized cell types and activities, such as the mammalian brain, are known to undergo relatively frequent AS [8,9].

The extent to which AS events in different cell and tissue types are regulated in a coordinated fashion to control specific cellular functions and processes is not known. Evidence for coordination of cellular functions by AS was recently provided by a study that employed a custom microarray to profile AS in mouse tissues. It was shown that deletion of the mouse gene that encodes Nova-2 (a neural specific AS factor) primarily affects AS events associated with genes encoding proteins that function in the synapse and in axon guidance [10]. In the absence of Nova-2, about 7% of AS events were detected to undergo differential inclusion levels between brain and thymus tissues [10], suggesting that additional neural specific AS events, and alternative exons specifically regulated in other tissues, might also be under coordinated control by specific splicing factors. The idea that AS coordinates the activities of functionally related genes is also supported by the results of studies on the *Drosophila *AS factor Transformer-2 (Tra2). Binding of Tra2 to a specialized exonic splicing enhancer element regulates the AS of transcripts encoding the transcription factors Doublesex and Fruitless, which activate sets of genes that are involved in sex determination and courtship behavior, respectively [11,12].

Current evidence indicates that tissue specific AS events may be regulated in some cases by different combinations of widely expressed factors and in other cases by cell/tissue specific factors [1,13,14]. In addition to the Nova AS regulators (Nova-1/2), several other proteins have been shown to participate in differential regulation of AS in the nervous system. These proteins include nPTB/BrPTB (a neural enriched paralog of the widely expressed polypyrimidine tract binding protein) and members of the CELF/Bruno-like, Elav, Fox, and Muscleblind families of RNA binding proteins, which can also regulate AS in other tissues [13-17]. Proteins that are known to be involved in tissue specific regulation of AS tend to recognize relatively short (typically five to ten nucleotides) sequences that are located in or proximal to regulated alternative exons. The binding of cell/tissue specific factors to these *cis*-acting elements is known to affect splice site choice by a variety of specific mechanisms that generally result in the promotion or disruption of interactions that are required for the recruitment of core splicing components during early stages of spliceosome formation [1,13,14].

In several cases, *cis*-acting sequences bound by AS regulators were initially identified by deletion and mutagenesis studies employing model pre-mRNA reporter constructs, in conjunction with *in vitro *or transfection based assays that recapitulate cell or tissue specific AS patterns [18]. In other studies, sequence motifs recognized by AS factors were identified by SELEX (systematic evolution of ligands by exponential enrichment) based methods and/or cross-linking/mapping approaches [19,20]. However, only a small number of physiologically relevant target AS events are known for most of the previously defined splicing factors, and systematic approaches to linking tissue regulated AS events with relevant *cis*-acting control sequences and cognate regulatory factors have only just been attempted [21,22]. Such studies will be important for defining the nature of the 'code' that underlies the regulation and coordination of cell and tissue type specific AS events.

In the present study, we used a new microarray to profile AS levels for thousands of cassette type alternative exons (namely, exons that are flanked by intron sequences and that are skipped or included in the final message) across a diverse spectrum of mouse tissues. Analyses of these data resulted in the identification of genes with single or multiple alternative exons that display tissue correlated AS levels and the discovery of many new central nervous system (CNS) associated AS events that are enriched in functionally related genes. A computational search also led to the identification of *cis*-acting motifs, many of which are new, that correlate strongly with CNS associated regulation of AS. Unexpectedly, many of these new motifs are located in neighboring constitutive exons and adjacent intron sequences. Together, our results suggest a widespread role for tissue coordinated AS events and associated *cis*-acting regulatory elements in controlling important functions in the mouse CNS.

## Results and discussion

**Figure 1:**
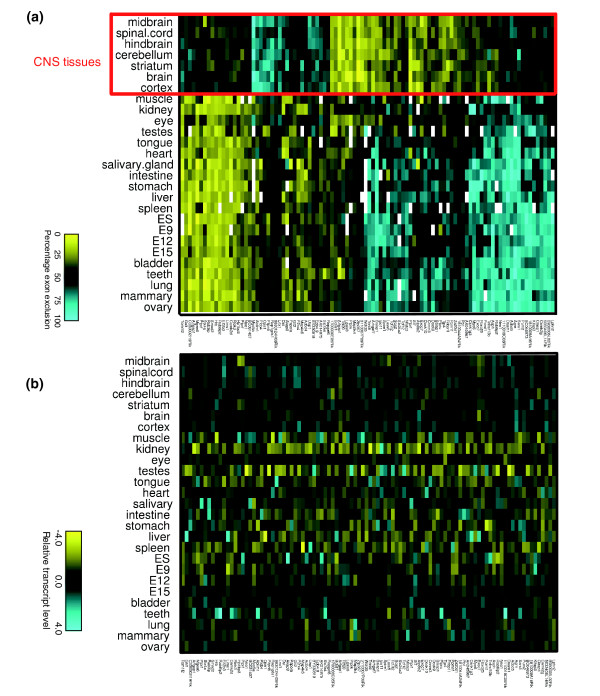
Identification of widely expressed genes with CNS specific regulation of AS. Microarray profiled genes with single or multiple alternative exons displaying differential alternative splicing (AS) in the central nervous system (CNS) were identified using statistical procedures that control for covariation in transcript levels (see Results and Materials and methods). **(a) **The top 100 genes with the most significant CNS associated AS levels are hierarchically clustered on both axes, based on their overall AS level similarity across 27 profiled tissues. **(b) **The corresponding transcript levels of the same genes, displayed in the same order. Color scales representing AS levels (percentage exon exclusion) and transcript levels (z-score scale) are shown below each panel. The z-score represents the number of standard deviations from the mean transcript level (center of the scale, in black) of the given event. Increasingly bright yellow represents lower transcript levels, and increasingly bright blue represents higher transcript levels. White rectangles in the AS clustergram indicate removed GenASAP (Generative Model for the Alternative Splicing Array Platform) values. These values were removed when transcript levels from the same genes (as measured using probes specific for constitutive exons on the microarray) were below the 95th percentile of the negative control probes.

**Figure 2:**
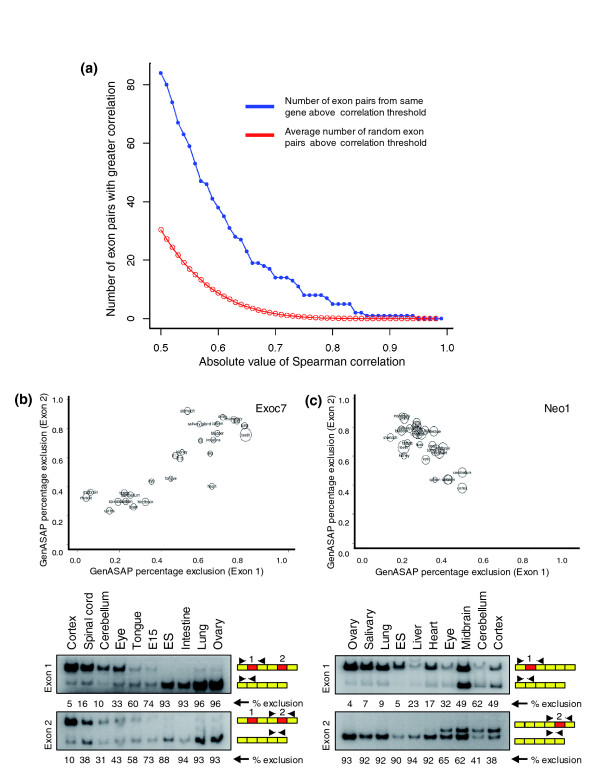
Coordination between AS events belonging to the same genes. **(a) **The correlation between the alternative splicing (AS) levels of pairs of alternative exons belonging to the same genes was assessed using standard Spearman correlation. The cumulative distribution plot shows the number of exon pairs (y-axis) observed to have an absolute value standard Spearman correlation higher than the value given on the x-axis. The blue curve with closed circles represents the number of observed exon pairs from the same gene above a given correlation threshold. The red curve with open circles is the average number of random pairs above a given correlation threshold, as determined using permutation resampling analysis (see Additional data file 1 [Materials and methods]). Also, representative examples of pairs of alternative exons with correlated splicing levels are shown **(b) **for a pair of exons with positively correlating inclusion levels from the Exo70 gene and **(c) **for a pair of exons with negatively correlating inclusion levels from the Neo1 gene. Upper panels show plots comparing the GenASAP percentage exon exclusion levels for each exon in a correlating pair, with the percentage exclusion levels for each exon separately plotted on the y-axis and x-axis. Circle sizes indicate relative transcript levels for the corresponding gene in each tissue shown, with larger circles indicating higher transcript levels. Lower panels show radioactive reverse transcription polymerase chain reaction (RT-PCR) assays performed with primer pairs targeted to constitutive exons flanking each alternative exon in a correlated pair. Percentage exclusion levels for each alternative exon, as measured using a phosphorimager (see Materials and methods), are shown. Additional examples of correlated pairs of exons validated by RT-PCR assays are shown in Additional data file 1 [Figure 2]. ES, embryonic stem cells.

Using a new AS microarray, we generated quantitative profiling data for 3,707 cassette-type AS events in 27 diverse mouse cells and tissues. These AS events were mined from expressed sequence tag (EST) and cDNA sequences represented by 3,044 UniGene clusters (see Materials and methods, below). The profiled tissues included whole brain, five brain subregions, spinal cord, three embryonic stages, embryonic stem cells, three muscle-based tissues (skeletal muscle, heart, and tongue), gastrointestinal and reproductive tissues, and several additional adult tissues. Quantitative, confidence-ranked estimates for percentage exclusion ('skipping') levels of each alternative exon were determined using the computational analysis tool GenASAP (Generative Model for the Alternative Splicing Array Platform) [23,24]. Confirming our previous findings [23,25], GenASAP percentage exon exclusion values ranking in the top one-third portion of the data correlated well (Pearson correlation coefficient > 0.80), with reverse transcription polymerase chain reaction (RT-PCR) measurements (see below and Additional data file 1 [Figures 1 and 2]).

In the present study, we used our dataset to detect alternative exons that display inclusion level differences specific to groups of physiologically related tissues, as compared with all other tissues. We also considered whether pairs of alternative exons belonging to the same genes have coordinated inclusion levels across the profiled tissues. From these analyses, we investigated which AS events may be coordinated functionally and potentially form AS-regulated networks, and which sequence elements in transcripts are likely to play a role in the regulation of functionally coordinated AS events.

### Tissue-specific regulation of AS in non-CNS tissues

AS events specific to groups of related tissues were initially analyzed. The use of the term 'specific' in this context, and below, refers to the detection of a statistically significant AS level difference in a group of tissues, relative to all of the other profiled tissues (see Additional data file 1 [Materials and methods] for details). We observed that about ten alternative exons displayed inclusion level differences in embryonic stem cells and the three whole embryo samples representing different stages of development, relative to the other profiled tissues. In addition, about ten alternative exons displayed pronounced inclusion level differences in the three muscle-based tissues (heart, skeletal muscle, and tongue), and five alternative exons displayed AS patterns common to both CNS and muscle tissues. Interestingly, some of the genes displaying AS differences in embryonic stem cells and embryonic samples are associated with regulation of development, and several of the genes with differential AS levels in muscle-based tissues are associated with muscle specific functions. These and other non-CNS-regulated AS events are described in Additional data file 1 and are listed in Additional data file 2. These findings suggest that AS could play an important role in coordinating gene functions in a tissue specific manner, although a larger set of tissue specific AS events is required to test this hypothesis.

### Regulation of alternative splicing in mouse CNS tissues

The largest numbers of tissue dependent AS events detected in our microarray data were associated with CNS tissues, with about 110 events displaying specific AS level differences (Figure 1a). This observation is consistent with previous reports providing evidence that AS is relatively frequent in the nervous system (see Introduction, above). Genes with these CNS tissue specific AS events were selected based on an analysis that controls for covariations in transcript levels in these tissues (see Additional data file 1). Approximately 35 additional CNS specific AS events were detected in genes that also displayed significant covariations at the transcript level across the tissues. These covariations could reflect effects on AS levels caused by co-transcriptional coupling [26] or independent CNS tissue dependent regulation at the transcriptional and splicing levels. However, we cannot exclude the possibility that some of the additional CNS specific AS events are detected as a consequence of measurement error resulting from varying transcript levels.

The probable functional relevance of the majority of the 110 most significant CNS-associated AS events is underscored by the observation that 60% of the alternative exons in this group could be detected in aligned human EST and cDNA sequences, whereas only about 24% of the non-CNS-associated alternative exons represented on the microarray could be detected in both human and mouse cDNA/EST sequences. This finding represents a statistically significant enrichment of conserved cassette alternative exons with detected CNS-associated AS levels, while controlling for variable cDNA/EST counts (*P *< 1 × 10^-16^; see Additional data file 1).

Consistent with this observation, and with the results of previous reports [21,27], we found that intron sequences within about 100 nucleotides of the CNS tissue regulated alternative exons (where AS regulatory motifs are often found; see below) more often overlap with the most conserved vertebrate genomic regions [28], as compared with the overlap observed for the corresponding intron sequences flanking non CNS tissue regulated alternative exons (see Additional data file 1; data not shown). For example, 50% of CNS specific AS events versus 25% in other events have at least 25 of the first 50 upstream intronic nucleotides located in these highly conserved elements, and 25% of CNS specific AS events versus 10% of other events have the entire first 50 nucleotides of the upstream intron covered by the conserved regions (Additional data file 1 [Figure 5]). A similar conservation level distribution was also observed in the 50 nucleotides downstream of the alternative exons, although with a smaller (10% to 20%) proportion of CNS-specific AS events versus non-CNS-specific AS events overlapping the most highly conserved regions (Additional data file 1 [Figure 5]). The proportion of CNS associated AS events that preserve reading frame in both isoforms is also significantly higher than observed for the other profiled AS events (81% versus 44%; *P *= 7.95 × 10^-14^, by Fisher's exact test). Only 8% of the CNS regulated exons have the potential to introduce a premature termination codon that could elicit nonsense mediated mRNA decay, in contrast to about 37% of the other AS events (*P *= 2.6 × 10^-6^, by Fisher's exact test). These results are consistent with recent findings indicating that a relatively small proportion of conserved AS events introduce premature termination codons [25,29], and further indicate that AS-coupled nonsense mediated mRNA decay is not a widespread mode of regulation of gene expression in the mammalian CNS. Taken together, our results thus indicate that a relatively large fraction of CNS associated AS events are under negative or purifying selection pressure to conserve sequences required to produce alternatively spliced forms; they are therefore likely to be functionally important.

We also examined the potential impact of the CNS regulated AS events at the protein level. The CNS associated AS events have the potential to result in partial or complete domain disruption in 13% (4/31) of cases, whereas 34% (201/599) of the non-CNS AS events represented on the arrays could result in such a change (*P *= 0.017, by Fisher's exact test). This difference, although based on a small sample size, is consistent with our observation that CNS regulated AS events are significantly enriched in conserved alternative exons, whereas AS events with the potential to disrupt conserved protein coding sequences are known to be significantly under-represented by conserved alternative exons compared with species-specific alternative exons [30]. In this regard, it is interesting to note that the alternative exons regulated in a CNS-specific manner are significantly shorter than the other profiled alternative exons (median of 75 nucleotides versus 102 nucleotides; *P *= 4.6 × 10^-7^, by Wilcoxon-Mann-Whitney test), whereas the alternative exons of AS events predicted to result in domain disruption have longer median exon lengths than those that are not predicted to result in domain disruption (116 nucleotides versus 99 nucleotides). Thus, the shorter alternative exon lengths of the CNS specific AS events appear to account, at least in part, for the lower proportion of predicted domain disruptions resulting from this set of exons. Given that these regulated exons are often conserved in human, it is interesting to consider that they may contribute numerous important roles, such as the formation and regulation of protein-protein interactions associated with neural specific complexes and pathways.

Remarkably, an extensive literature search revealed that 50 (40%) of the top 125 genes (ranked according to the significance of the CNS associated AS level difference) have a reported specific functional link with the nervous system. Nervous system specific functions of genes containing CNS regulated AS events are listed in Table 1, and a more detailed description of the roles of some of these genes is provided in Additional data files 2 and 3. Because about 20% of the genes with CNS-regulated AS in our list have not been characterized on any level or are poorly characterized, the proportion of genes with specific functional roles in the nervous system is likely to be considerably higher than 40%.

**Table 1:** List of genes with CNS tissue-specific AS regulation

Category	Gene name	Accession	CNS function/phenotype
Signaling	*Arhgef7*	AF247655	Synapse formation
	*Camk2d*	BC042895	Phosphorylates PSD-95 in postsynaptic density
	*Camk2g*	BU560927	Phosphorylates PSD-95 in postsynaptic density
	*Git2*	BU614137	Postsynaptic density interactions
	*Map4k6*	BC011346	Axon guidance
	*Map3k4*	AK122219	Neural tube development
	*Opa1*	AK050383	Neuropathy
	*Plcb4*	BC051068	Metabotropic glutamate neurotransmitter signaling pathway, synaptic depression
	*Ptprf*	AF300943	Synapse formation
	*Ptprk*	L10106	Neurite outgrowth
	*Rapgef6*	BC019702	Neurogenesis, gliogenesis
	*Rap1ga1*	BY234371	Neurite outgrowth
	*Vav2*	U37017	Neurite outgrowth
Cytoskeleton	*Ablim1*	AK122196	Axon guidance
	*Clasp1*	CA326660	Axon guidance
	*Dst*	AK037206	Neurodegeneration, myelination, retrograde axonal transport
	*Kifap3*	D50367	Axonal vesicle transport
	*Myo5a*	CA469310	Synaptic vesicle transport
	*Myo6*	U49739	Neurotransmitter endocytosis
	*Syne1*	BC041779	Synaptic nuclear envelope anchor at neuromuscular junction
	*Tmod2*	AU035865	Learning/memory, long-term potentiation
Vesicular transport	*Dlgh4*	D50621	Synaptic vesicle maturation
	*Dnm1*	BC034679	Synaptic vesicle endocytosis
	*Exoc7*	AF014461	Neurotransmitter receptor membrane targeting
	*Rab6ip2*	AF340029	Neurotransmitter release
	*Snap23*	AA450833	Neurotransmitter exocytosis
	*Sgip1*	BC017596	Neural energy balance regulation
	*Syngr1*	AK010442	Synaptic vesicle component
mRNA processing	*Adarb1*	AF525421	Glutamate receptor mRNA editing
	*Papola*	NM_011112	Regulated polyadenylation at synapses
Transcription factors	*Apbb1*	BM950527	Learning/memory, Alzheimer's disease
	*Nfatc3*	BC021835	Axon outgrowth, neuronal survival, astrocyte function
	*Tcf12*	X64840	Transcription factor involved in neuronal plasticity, CNS development
Tight junctions	*Baiap1*	AK032350	Nervous system signaling
	*Magi3/6530407C02Rik*	AF213258	CNS signaling, neurotransmitter receptor regulation
	*Tjp4*	BU612515	Interacts with synaptic protein
Ion channels	*P2rx4*	AF089751	Neurotransmitter receptor
	*Slc24a2*	NM_172426	Calcium ion channel in axon terminals
Other functions	*Agrn*	BG803812	Regulates formation of postsynaptic structure at the neuromuscular junction
	*Kidins220/C330002I19Rik*	AK083260	Neural signaling
	*Mgea6*	BI962144	Meningioma antigen
	*Mgrn1*	BY567496	Neuronal degradation, astrocytosis
	*Neo1*	Y09535	Axon guidance, neuronal survival
	*NIBP/1810044A24Rik*	BC034590	Neurite outgrowth, nerve growth factor signaling
	*Pcmt1*	AA981003	Memory, synaptic function, seizures
	*Sca2*	AF041472	Neurodegenerative disease spinocerebellar ataxia
	*Serpinh1*	BB613516	Glial cell protection, CNS development

Microarray-profiled genes displaying central nervous system (CNS) tissue specific alternative splicing regulation are listed, along with a description of known functions of the genes in the nervous system. More detailed information on the same gene list, including published information on the CNS tissue regulated exons and relevant literature, is provided in Additional data file 3.

Consistent with the previous observation that about 7% of AS events are differentially regulated between neocortex and thymus by the AS regulator factor Nova-2 [10] (see Introduction, above), seven of the 110 CNS regulated AS events identified in our analysis are common to 50 neocortex regulated events reported in this previous study. Moreover, 16 of the CNS regulated AS events identified in our study overlap with a set of brain specific alternative exons reported by Sugnet and coworkers [21] in another microarray profiling study involving mouse tissues. An additional 54 AS events reported to be brain specific in this latter study also overlapped with AS events represented by probes on our microarray. However, our microarray data and analyses, as well as the RT-PCR experiments in the present study and in that by Sugnet and coworkers, do not provide support for more than a few of these as being brain specific. In contrast, 17 out of 17 (100%) of the CNS tissue specific AS events from our list of 110 were subsequently confirmed by RT-PCR assays as having CNS tissue specific splicing patterns (Figure 2; also see Additional data file 1 [Figures 1 and 2]; data not shown). The results of extensive literature searches (see Additional data file 1) further indicate that approximately two-thirds or more of the CNS associated AS events identified from our microarray data either have not been reported, or if reported they were not previously known to undergo nervous system specific AS (see below).

### Different contributions of alternative splicing and transcriptional regulation in the mouse CNS

We then considered the extent to which the set of genes with regulated neural specific AS events in our data overlap the set of genes regulated in a neural specific manner at the transcriptional level (the total level of the exon included and exon excluded splice variants displaying significant CNS specific changes). Using information provided by the microarray probes targeting the constitutive exons flanking each alternative exon, we identified about 200 genes that have CNS associated changes at the transcript level, as represented by statistically significant changes relative to most of the other profiled tissues (see Additional data file 1 [Materials and methods]). Consistent with previous findings indicating that AS and transcript level regulation control different subsets of genes in mammalian tissues [23,30,31], the majority (about 80%) of the approximately 150 genes with the most significant CNS associated AS levels do not overlap with the approximately 200 genes regulated in a CNS specific manner at the transcriptional level (Additional data file 1 [Figure 4]). Many of the remaining (about 20%) of genes could reflect regulation of AS via co-transcriptional coupling or AS events that are independently regulated at the AS and transcriptional levels.

### Coordination between AS events belonging to the same genes

In addition to the detection of individual alternative exons that display regulatory patterns associated with single tissues or groups of physiologically related tissues, we investigated whether pairs of alternative exons belonging to the same genes display tissue coordinated AS levels. Previous studies of EST/cDNA sequences identified a few cases in which different alternative exons belonging to the same genes appear to be coordinated [32,33]. However, these studies did not address whether multiple exons in the same genes can be co-regulated in a tissue-dependent manner, or the extent to which coordination between alternative exons occurs in a large number of genes. Approximately 500 of the 3,044 genes represented on our microarray contain between two and five alternative exons. The AS levels for all pair-wise combinations of the alternative exons belonging to the same genes, with sufficiently high transcript levels in 20 or more tissues, were compared using both standard and partial Spearman correlation. The statistical significance of observed correlations was assessed by comparing the observed number of correlated pairs of exons at a given correlation level with the average number of pairs at the same correlation level obtained from 1,000 random samples of pairs of exons belonging to different genes (Figure 2a; see Additional data file 1 for details).

Approximately 15 of the pairs of alternative exons have significantly correlating (absolute standard Spearman correlation ≥ 0.70) inclusion levels across the tissues, with an expected false-positive detection rate of one exon pair (Additional data file 4; also see Additional data file 1 for details). However, higher than expected numbers of exon pairs with correlated AS levels are observed over a wide range of lower correlation levels (Figure 2a). For example, 38 pairs of exons display an absolute standard Spearman correlation of 0.60 or greater, although with an expected false positive detection rate of six to ten exons. Approximately 65% of the pairs of exons displayed tissue dependent changes in inclusion levels in the same direction (positive correlation), whereas 35% of the pairs displayed tissue specific AS level changes in the opposite direction (negative correlation; Figure 2 and Additional data file 4). Six pairs of exons with significantly correlating AS levels were analyzed by RT-PCR assays in ten of the 27 tissues (Figure 2 and Additional data file 1 [Figure 2]). In each case the tissue RNA samples were selected for analysis on the basis of availability and displaying a broad range of inclusion levels for each exon in a coordinated pair. All six pairs displayed the overall expected AS level differences between the tissues, indicating that our predictions for correlated AS levels between exons belonging to the same genes are accurate.

**Figure 3:**
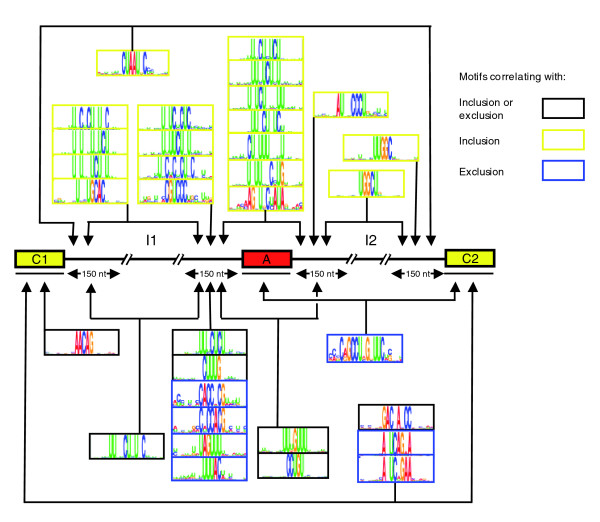
Detection of new motifs in exons and introns that correlate with CNS regulated AS events. Motifs correlating with central nervous system (CNS) associated alternative splicing (AS) levels were detected in exon sequences (C1, A, C2) and intron sequences (I1, I2) using the SeedSearcher algorithm [41]. *Ab initio *searches for motifs were performed in the individual exon and intron sequences, and in concatenations of intron/exon sequences. Motifs enriched in these locations, as indicated by lines with arrowheads, are correlated with either increased inclusion (yellow boxes), increased exclusion (blue boxes), or a change in inclusion (black boxes), respectively.

Exons with high positive correlation (at a standard Spearman correlation ≥ 0.6) are mostly within one to four exons of each other, with a median of two intervening exons (Additional data file 1 [Figure 3]). In contrast, exon pairs with high negative correlation (at a standard Spearman correlation ≤ -0.60) have a median of four intervening exons, and exon pairs that are not highly correlated (with an absolute standard Spearman correlation < 0.6) have a median of four intervening exons (Additional data file 1 [Figure 3]). The difference in intervening exon numbers between the positively correlated pairs of exons and pairs of exons that are not highly correlated is statistically significant (*P *= 0.021, by Wilcoxon-Mann-Whitney rank sum test). Consistent with these results, exon pairs displaying positive correlation are also significantly closer to each other in terms of nucleotide length, as compared with pairs of exons with high negative correlation or without high correlation (Additional data file 1 [Figure 3]). In a few of the cases shown in Additional data file 4, pairs of alternative exons with significant positive correlation are adjacent to each other. One example is a pair of alternative exons in the gene encoding Agrin, a proteoglycan that functions in the aggregation of acetylcholine receptors in postsynaptic membranes, which is a key step in neuromuscular junction development. Consistent with our microarray data indicating that this pair of exons has increased inclusion levels in CNS tissues relative to the other profiled tissues, it has been reported that the same pair of exons can be included in nervous system tissues but are excluded in all other tissues examined [34]. These results suggest the interesting possibility that proximal pairs of alternative exons, whether adjacent or separated by at least one intervening exon, may positively influence each other and thereby facilitate tissue specific coordination of AS events belonging to the same genes.

As in the case of the pair of the positively correlated exons in Agrin transcripts, the levels of inclusion of exons belonging to a correlated pair are generally highly similar among the various CNS tissues (Figure 2 and Additional data file 1 [Figure 2]). Consistent with this observation and the analyses described above, about 50% of genes with significantly correlated pairs of AS events are known to have neural specific functions (Additional data file 4). In other examples, a pair of positively correlated alternative exons with distinct neural specific splicing levels is detected in transcripts from the *Exoc7/Exo70 *gene (Figure 2b), and a pair of negatively correlated alternative exons, with each exon also displaying distinct levels in CNS tissues, is detected in transcripts from the Neogenin (*Neo1*) gene (Figure 2c). *Exoc7/Exo70 *is a component of exocyst complex that is involved in vesicle-mediated exocytosis and functions in membrane targeting of neurotransmitter receptors for γ-aminobutyric acid (GABA) and *N*-methyl-D-aspartate [35-37], and *Neo1 *is a widely expressed cell surface receptor that is involved in axon guidance and in the regulation of neuronal survival [38,39]. Collectively, these findings indicate that pairs of alternative exons belonging to the same genes can be regulated in a coordinated manner in different mouse tissues, and that many of these pairs of exons are probably associated with CNS specific functions.

### Different groups of functionally related genes display CNS associated AS and transcript level regulation

Subsets of AS events regulated in a tissue dependent manner may serve to coordinate specific biologic functions and therefore are of considerable interest. To assess more systematically the functions of the genes containing alternative exons with CNS associated AS levels, and to address whether these genes operate in common cellular processes and pathways, we considered whether genes with the most significant CNS associated AS level differences are enriched in Gene Ontology (GO) terms. Enrichment was observed for terms including the following: GTPase-based signaling, cell-cell signaling, cytoskeletal organization and biogenesis, vesicular mediated transport, transmission of nerve impulse, and neurophysiologic process (false discovery rate < 0.15; Table 2). Enrichment of these terms appears to be specific to the group of genes with CNS regulated AS events, because a group of about 100 genes from our data that contain alternative exons regulated in non-CNS tissues are not enriched for the same terms (data not shown). Approximately 30% of the genes in our list were linked to one or more annotations associated with these GO processes, as was also supported by independent information provided by manual literature searching. Although the genes associated with these GO processes are generally widely expressed, most have documented nervous system specific functions (Table 1). In addition, some of the genes are known to encode proteins that physically interact or function in the same biological pathways (see Additional data file 1 and Conclusions [below] for more information).

**Table 2:** Gene Ontology terms enriched in genes with CNS specific AS and transcript levels

Function/pathway	GO term	CNS count	CNS proportion	Total count	Total proportion	FDR
**GO term enrichment in genes with CNS tissue specific AS levels**						
Signaling pathways	Cell-cell signaling	7	0.065	17	0.011	0.02820
	Rho guanyl-nucleotide exchange factor activity	5	0.047	8	0.005	0.02820
	Guanyl-nucleotide exchange factor activity	7	0.065	20	0.013	0.04400
	Rho GTPase binding	3	0.028	3	0.002	0.04400
	GTPase regulator activity	11	0.103	54	0.035	0.08280
Vesicular transport	Vesicle-mediated transport	13	0.121	64	0.042	0.04400
Cytoskeleton	Cytoskeletal protein binding	11	0.103	52	0.034	0.06720
	Actin binding	8	0.075	32	0.021	0.09650
Nervous system	Transmission of nerve impulse	5	0.047	13	0.008	0.09770
	Neurophysiologic process	6	0.056	20	0.013	0.10800
Other functions	Protein binding	49	0.458	449	0.291	0.03520
	Plasma membrane	17	0.159	118	0.077	0.10800
**GO term enrichment in genes with CNS tissue specific transcript levels**						
Signaling pathways	Cell-cell signaling	12	0.085	34	0.015	0.00022
	Cell communication	35	0.246	334	0.143	0.03030
	Insulin secretion	3	0.021	5	0.002	0.08710
	Peptide hormone secretion	3	0.021	5	0.002	0.08710
	Ionotropic glutamate receptor activity	2	0.014	2	0.001	0.09940
	G-protein-coupled receptor binding	2	0.014	2	0.001	0.09940
	Glutamate-gated ion channel activity	2	0.014	2	0.001	0.09940
	Excitatory extracellular ligand-gated ion channel activity	3	0.021	6	0.003	0.09940
	Cyclic-nucleotide-mediated signaling	3	0.021	7	0.003	0.13900
	cAMP-mediated signaling	3	0.021	7	0.003	0.13900
	G-protein coupled receptor protein signaling pathway	9	0.063	58	0.025	0.14800
Secretory pathways	Secretory pathway	10	0.070	45	0.019	0.02340
	Secretion	10	0.070	51	0.022	0.04610
	Regulated secretory pathway	4	0.028	11	0.005	0.09940
Cytoskeleton	Microtubule	8	0.056	40	0.017	0.08980
	microtubule associated complex	5	0.035	19	0.008	0.10800
	Cytoskeleton	16	0.113	136	0.058	0.14800
	Microtubule-based process	7	0.049	39	0.017	0.14800
Nervous system	Transmission of nerve impulse	9	0.063	26	0.011	0.00456
	Postsynaptic membrane	7	0.049	16	0.007	0.00512
	Synaptic transmission	8	0.056	25	0.011	0.01100
	Nervous system development	14	0.099	75	0.032	0.01500
	Synaptosome	5	0.035	12	0.005	0.03030
	Neurophysiologic process	9	0.063	43	0.018	0.04820
	Neurogenesis	9	0.063	46	0.020	0.07040
	Neurotransmitter secretion	4	0.028	10	0.004	0.08710
	Neuron development	7	0.049	33	0.014	0.09940
	Neuron differentiation	7	0.049	36	0.015	0.11400
	Regulation of neurotransmitter levels	4	0.028	14	0.006	0.14800
Other functions	Membrane fraction	11	0.077	54	0.023	0.02340
	System development	15	0.106	79	0.034	0.01020
	Localization	44	0.310	487	0.209	0.09940

Gene Ontology (GO) terms significantly enriched in the top approximately 100 genes with the most significant central nervous system (CNS) tissue specific alternative splicing (AS) levels, and the top approximately 200 genes with the most CNS specific transcript levels are shown. CNS counts (number of times a GO term appears in the CNS tissue regulated group) and total counts (number of times a GO term appears in the total group of microarray-profiled genes with sufficient expression across 15 tissues) are shown. Proportions in each group are also shown. FDR denotes the false discovery rate of a GO term. This value represents the expected proportion of false positive GO terms out of all positive GO terms. Only GO terms with a FDR below 0.15 are shown.

Many of the genes containing CNS tissue regulated alternative exons encode factors belonging to Rho, Rap, Rab, and Arf GTPase mediated signaling pathways. A subset of these genes are associated with neural specific functions such as dendrite morphogenesis, neurite growth, synapse formation, and axon guidance (see Table 1 and Additional data file 1). CNS associated AS events were also detected in multiple members of the mitogen-activated protein kinase and calmodulin kinase signaling pathways, and in different phosphatases, some of which are involved in signaling in the nervous system (Table 1 and Additional data file 1). CNS regulated AS events were detected in multiple genes associated with actin, myosin, and microtubule based cytoskeletal components. Genes among this group have neural specific functions associated with vesicular transport, axon pathfinding/neurite outgrowth, glutamate receptor endocytosis, and neuroepithelial development (Table 1; see Additional data file 1). A prominent feature of the genes containing CNS associated AS events is their functional association with different stages of vesicle trafficking in neurons, such as synaptic vesicle endocytosis and exocytosis. Other functional categories containing multiple CNS specific events were mRNA processing, transcription factors, tight junctions, and ion channels (Table 1). These observations support the conclusion that signaling pathways, the cytoskeleton, and vesicular transport are highly regulated by AS in the mouse CNS.

Interestingly, genes regulated in a CNS-specific manner at the transcript level are enriched in an overlapping yet distinct set of GO annotation terms compared with the genes with CNS associated AS events (Table 2). These terms include synaptic function, nerve impulse and transmission, nervous system development, cytoskeletal organization and biogenesis, and secretory pathways. This supports the conclusion that AS and transcription are regulated in a CNS specific manner to coordinate the activities of mostly distinct genes that operate in partially overlapping processes and pathways.

### Intronic and exonic motifs correlated with CNS-regulated AS

We next aimed to identify *cis*-acting motifs, either known or novel, that comprise the 'code' underlying the regulation of CNS associated AS events. Previous studies conducted to identify motifs associated with tissue-dependent regulation of AS have largely focused on searches within regulated alternative exons or the immediate flanking intron sequences of alternative exons [3,4]. However, regulation of AS can also involve more distally acting *cis *elements located in introns or in neighboring exons [22,40]. Also, it is possible that some *cis *elements are not confined to a specific region but rather can function from one of two or more locations, for example from an intron location that is either upstream or downstream of a regulated alternative exon. We therefore performed a systematic *ab initio *motif search covering the following sequences: alternative exons, constitutive exons located directly upstream and downstream of each alternative exon, and 150 nucleotides of intron sequence flanking each of these three exons (see Figure 3 and Additional data file 1 for details). We also searched different concatenations of these sequences in order to detect motifs that may function from one of two possible locations.

The *ab initio *search was performed using a modified version of the SeedSearcher algorithm [41] (see Additional data file 1 for details). This algorithm enabled us to identify motifs that discriminate the sequences associated with the top approximately 100 CNS regulated AS events (summarized above) from the corresponding sequences associated with non-CNS-regulated AS events. Specifically, we searched for motifs that best discriminate AS events belonging to groups that display a significant increase in exon inclusion in CNS tissues, a significant increase in exon exclusion in CNS tissues, or either an increase or decrease in inclusion in CNS tissues, as compared with the non-CNS tissues. Each search was performed for motifs with a length of between five and 20 nucleotides and with various degrees of sequence flexibility. The statistical significance of each motif was computed and assigned a *P *value that was corrected for multiple hypotheses testing, and each motif was also compared against a database of previously reported motifs associated with splicing (see Additional data file 1 for details on motif scoring and comparison procedures). Because sequence conservation reflects selection pressure acting to preserve biologic activity, it can be used as a proxy to assess the probable functional importance of motifs. Accordingly, we also analyzed the relative conservation levels of the SeedSearcher motifs in the corresponding intron and exon regions of the orthologous human genes, and the statistical significance of detected conservation was determined (see Additional data file 1 for details).

All 39 of the SeedSearcher motifs found to be significantly enriched in the CNS regulated AS events (corrected *P *< 0.05) are shown in Additional data file 5, alongside any known similar motifs. Of these motifs, 26 had at least 20 occurrences in the three groups defined above, and this number of occurrences facilitated further analysis of these motifs for statistically significant, relative conservation levels. Seventeen of the 26 motifs were found to be significantly more conserved than the surrounding regions (binomial *P *< 0.05; see Additional data files 1 and 10 for details). Finally, we also directly searched for enrichment of *cis *elements with a previously described link to regulation of AS in the nervous system (Additional data file 6). The results of this search, as well as the *ab initio *search, are described in more detail below.

Figure 3 illustrates part of a putative code for CNS AS regulation based on the results of our *ab initio *search. Apart from interesting features of the motifs themselves, a number of important general observations can be made. First, we note that no motifs are found in the alternative exons, whereas several motifs are located in the neighboring constitutive (C1 and C2) exons and in the intron sequences flanking these and the alternative exons. Second, some motifs are detected when sequence regions were concatenated, indicating that they could function in a spatially flexible manner and do not have to reside within a specific exon or intron location. Third, there is a high enrichment for variations of C/U-rich motifs. These predominantly reside within the 150 nucleotide intron region immediately upstream of alternative exons, although some C/U-rich motifs are also found within the 150 nucleotide intron region downstream of alternative exons. These motifs are specific to these regions, because they are not significantly enriched in the 150 nucleotide intron regions immediately flanking the C1 and C2 exons. Moreover, none of the motifs shown in Figure 3 and listed in Additional data file 5 were enriched in exons or flanking intron regions of exons that are upstream and downstream of the C1-A-C2 region (data not shown). Finally, we note that the most significantly enriched motifs, and the intronic C/U-rich motifs in particular, are more often associated with alternative exons that display preferential inclusion in CNS tissues, rather then preferential exclusion.

The C/U-rich motifs resemble binding sites for nPTB and PTB, which are known to function in the regulation of alternative exon inclusion in the nervous system (see Introduction) [42,43]. In particular, consistent with the observation that these motifs are more strongly associated with increased inclusion of alternative exons in CNS tissues relative to other profiled tissues, previous studies on the neural specific c-src N1 exon and an exon within the GABA(A) receptor gamma2 pre-mRNA suggested that binding of nPTB to pyrimidine-rich sequences adjacent to these exons can promote their inclusion [42,43].

Consistent with the results from the *ab initio *search, C/U-rich motifs were also found to be the most significantly enriched (hypergeometric *P *about 10^-6^) when directly searching using subsequences of known motifs, including those shown in previous experiments to directly bind nPTB and PTB (Additional data file 6). Many of the C/U-rich motifs identified in the directed searches resemble those identified by the *ab initio *search. However, in both searches, the identified motifs are considerably shorter and more degenerate than those inferred previously by experimental approaches and, as such, could represent the core recognition sites for nPTB/PTB or potentially other AS regulatory factors that specifically recognize pyrimidine-rich regions to regulate neural specific splicing. The C/U-rich motifs that we detected are different from the UGYUUUC motif that Sugnet and coworkers [21] found to be enriched in the 150 nucleotide intron flanks upstream of the alternative exons, which they scored as having increased inclusion in nervous system tissues. These authors did not observe enrichment of the sequence CUCUCU, which is known to bind PTB/nPTB, when they searched the intron flanks of their predicted nervous system-regulated exons, whereas this sequence was found to be significantly and specifically enriched in the sequences upstream of alternative exons displaying increased inclusion in CNS tissues in our data (Additional data files 6 and 7).

The differences between our findings and the results reported by Sugnet and coworkers could be due to the different sets of AS events analyzed (see above) as well as differences between the motif search algorithms implemented in the two studies. To investigate the latter possibility, we employed the Improbizer algorithm described by Sugnet and coworkers to identify and score motifs enriched in our set of CNS regulated AS events. The Improbizer searches resulted in detection of 20 statistically significant (*P *≤ 0.05; see Additional data file 1 for details) position-specific scoring matrix (PSSM) based motifs (Additional data file 9). Consistent with the results obtained with SeedSearcher, Improbizer detected enrichment of several C/U-rich motifs in the intron regions flanking the regulated alternative exons, and only one motif was detected in the regulated alternative exons, although this motif barely passed the score threshold. There are obvious similarities between many of the 20 Improbizer PSSM motifs and the 39 SeedSearcher motifs, and in some cases combinations of multiple SeedSearcher motifs appear to be similar to individual Improbizer PSSMs (but not necessarily *vice versa*). It is also noteworthy that although the UGYUUUC motif previously reported by Sugnet and coworkers was not detected in intron sequences flanking the CNS regulated exons in our data, Improbizer did report another motif (UUUSYUU) that matched one of the SeedSearcher motifs (UUUGYUU; see Additional data file 5). Our comparative results thus indicate that both differences in the sets of AS events analyzed as well as in the motif search procedures probably account for differences between the numbers and types of motifs detected in the two studies.

In addition to the detection of strong enrichment of C/U-rich motifs, the directed searches on our dataset revealed a relatively modest enrichment (P about 10^-4^) of motifs corresponding to the binding sites for Fox-1/2 and Nova-1/2 motifs. This is not surprising because, as mentioned before, Nova proteins probably regulate about 7% of neural specific AS events [10], and Fox proteins are known to regulate AS in several cell and tissue types in addition to nervous system tissues [15]. The detection of these and the C/U-rich motifs in expected regions (see Additional data file 7) indicates that our microarray data and search procedures are reliable and probably result in the identification of functionally relevant *cis*-acting sequences associated with CNS tissue regulated AS.

Importantly, the *ab initio *searches resulted in the detection of many motifs that are more highly enriched than those found in the directed searches, and several of these motifs appear to be novel (Figure 3 and Additional data files 5 and 8). For example, two closely related motifs with the sequences ANUCAGNA (where N represents a position where any base may occur) and ANUCNGAA are enriched in C1 exon and C1-C2 concatenation, and are associated with increased CNS tissue specific exclusion of the adjacent alternative exon. Another motif with the sequence CUAAUNC is enriched in the C2I2 intron sequence and C1I1-C2I2 concatenation and is associated with CNS tissue specific inclusion of the adjacent alternative exons. These motifs could correspond to the recognition sites for as yet unidentified CNS tissue specific splicing factors that function by binding to constitutive exons and intron sequences flanking constitutive exons, respectively. It is interesting to note in this regard that recent evidence suggests that Nova dependent regulation of alternative splicing involves clusters of Nova binding sites, some of which are located in proximal constitutive exons, as well as in the intron regions flanking these constitutive exons [22].

In summary, our findings suggest a previously unanticipated and widespread role for C/U-rich motifs bound by AS regulators such as nPTB and PTB in CNS specific AS. In addition, our data suggest that CNS specific AS involves many new motifs and as yet unidentified factors that bind to these motifs. Many of these new regulatory elements are predicted to function from the proximal constitutively spliced exons and their flanking intron sequences.

## Conclusion

Using a new AS microarray applied to the profiling of 27 diverse mouse cell and tissue types, we detected a large number of new examples of tissue dependent differential regulation of AS. Most of these regulated AS events were observed in CNS tissues, and approximately two-thirds have not previously been reported in the literature. At least 3% of pairs of alternative exons belonging to the same genes appear to be spliced in a coordinated manner across the profiled cells and tissues, with many exon pairs displaying the most distinct inclusion level differences in nervous system tissues. Pairs of alternative exons that are spliced in a positively correlated manner across mouse cells and tissues tend to be relatively close to one another (often separated by a single intervening exon), and this implies that coordination between AS events belonging to the same genes may involve communication between splicing factors assembled at proximally located splicing signals.

**Figure 4:**
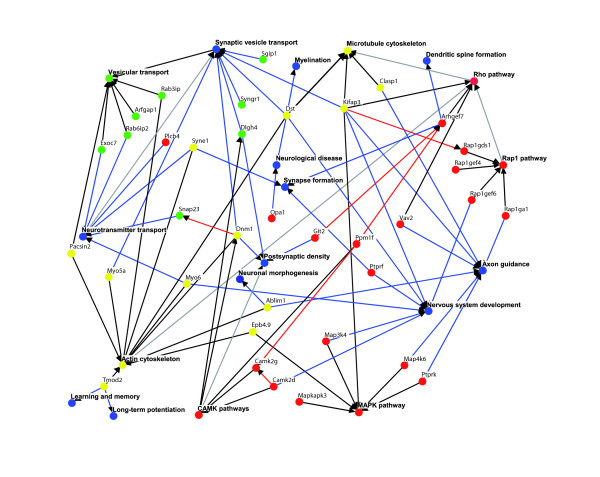
Network diagram comprising genes and functional processes associated with regulated AS events in the CNS. Experimental evidence supporting interactions, pathway and functional relationships among genes with microarray detected central nervous system (CNS) specific alternative splicing (AS) events was retrieved from the literature and from the Online Predicted Human Interaction Database [45], and used to construct a network diagram using the Osprey program [46]. Yellow nodes denote cytoskeletal pathways/genes, green nodes denote vesicle-mediated transport pathways/genes, red nodes denote signaling pathways/genes, and blue nodes denote CNS functions. Red edges denote protein-protein interactions, black edges denote gene-pathway associations, blue edges denote gene-CNS function associations, and gray edges denote pathway-pathway or pathway-CNS function associations. Pathways and CNS functions are in bold letters. Gene names are the NCBI Entrez Gene standard gene symbols.

Approximately half of the genes containing single and pairs of exons that are differentially spliced in CNS tissues have known neural specific functions. The CNS associated AS events we have detected by microarray profiling are significantly enriched in genes with GO terms related to GTPase based signaling, vesicular transport and cytoskeletal functions, as well as nervous system specific GO terms. Similar to the proposed role for Nova proteins in the coordinated regulation of alternative exons belonging to genes associated with functions at the synapse [11,22] (see Introduction, above), our results suggest a more widespread role for coordinated AS events to modify the proteins of widely expressed genes, such that these proteins can operate in multiple different CNS associated functions and pathways. Based on documented experimental evidence (see Additional data file 1), we have constructed a network illustrating possible connections between many of the GO enriched genes and their associated nervous system specific functions (Figure 4). This network highlights the nature of the possible interactions between widely expressed genes that have CNS regulated AS events (see the legend to Figure 4 for additional information).

New and known motifs were identified that correlate strongly with the CNS regulated AS events. Our results thus provide a large number of new CNS regulated AS events, many of which are associated with functionally related genes, as well as detailed information on sequence motifs that are predicted to regulate these CNS-associated AS events. These motifs probably comprise part of the sequence 'code' underlying the regulation of CNS tissue specific AS. As such, the data resulting from our analyses should provide a valuable resource for establishing molecular mechanisms by which the neural specific functions of widely expressed genes are regulated and coordinated at the level of AS.

## Materials and methods

### Identification of AS events in mouse transcripts

The detection of AS events was performed essentially as previously described [23,30].

### Microarray hybridization, image processing, and data analysis

Microarray design, hybridization and data analysis for 3,707 mouse cassette AS events from 3,044 UniGene clusters (represented on a single 44 K microarray manufactured by Agilent Technologies, Inc., Santa Clara, CA 95051, USA) was performed essentially as described previously [23,24,44]. Information on AS events represented on the mouse microarray, and GenASAP estimates for percentage exon exclusion levels for the cassette AS events in 27 mouse tissues are provided in Additional data file 1. Methods for detection and analysis of single tissue regulated AS events, and correlated pairs of alternative exons, are described in Additional data file 1.

### Motif detection and analysis

Detection of motifs enriched in exon and intron sequences associated with CNS regulated AS events was performed using a variant of the SeedSearcher algorithm [41]. Details on methods for motif searches, the assessment of statistical significance of individual motifs, and comparisons of SeedSearcher detected motifs with previously identified motifs are provided in Additional data file 1.

### RT-PCR assays

RT-PCR reactions were carried out as described previously [25].

## Additional data files

The following additional data are available with the online version of this paper. Additional data file 1 provides supplemental information on genes with microarray detected tissue specific AS levels, additional details regarding the materials and methods used, and additional illustrations. Additional data file 2 provides information on tissue specific AS events. Additional data file 3 provides information on CNS regulated AS events. Additional data file 4 provides information on correlated pairs of AS events belonging to the same genes. Additional data file 5 summarizes motifs associated with CNS specific AS events detected from *ab initio *searches. Additional data file 6 summarizes experimentally defined sequences/motifs associated with neural specific AS used for searches. Additional data file 7 summarizes experimentally defined motifs/subsequences significantly enriched in exons and introns associated with CNS regulated AS events identified in the AS microarray data. Additional data file 8 provides the number and statistical significance of *ab initio *motifs detected at each exonic and intronic location, and in each group. Additional data file 9 summarizes motifs associated with CNS specific AS events detected by searching with the Improbizer program. Additional data file 10 summarizes conservation levels of motifs associated with CNS specific AS events detected from *ab initio *searches. Additional data file 11 shows GenASAP values and C1-A-C2 exon sequences for 3,707 AS events profiled in 27 mouse tissues.

## Supplementary Material

Additional data file 1 Provided is supplemental information on genes with microarray detected tissue specific AS levels, additional details on Materials and Methods, and additional Figures (1-5). Click here for file

Additional data file 2 Provided is information on tissue specific AS events. Click here for file

Additional data file 3 Provided is information on CNS regulated AS events. Click here for file

Additional File 4 Provided is information on correlated pairs of AS events belonging to the same genes. Click here for file

Additional data file 5 Summarized are motifs associated with CNS specific AS events detected from *ab initio *searches. Click here for file

Additional data file 6 Summarized are experimentally defined sequences/motifs associated with neural specific AS used for searches Click here for file

Additional data file 7 Summarized are experimentally defined motifs/subsequences significantly enriched in exons and introns associated with CNS regulated AS events identified in the AS microarray data. Click here for file

Additional data file 8 Summarized are the number and statistical significance of *ab initio *motifs detected at each exonic and intronic location, and in each group. Click here for file

Additional data file 9 Summarized are motifs associated with CNS specific AS events detected by searching with the Improbizer program. Click here for file

Additional data file 10 Shown are conservation levels of motifs associated with CNS specific AS events detected from *ab initio *searches. Click here for file

Additional data file 11 Provided are GenASAP values and C1-A-C2 exon sequences for 3,707 AS events profiled in 27 mouse tissues. Additional microarray data has been deposited in GEO under accession GSE8081. Click here for file

## References

[B1] Smith CW, Valcarcel J (2000). Alternative pre-mRNA splicing: the logic of combinatorial control.. Trends Biochem Sci.

[B2] Graveley BR (2001). Alternative splicing: increasing diversity in the proteomic world.. Trends Genet.

[B3] Matlin AJ, Clark F, Smith CW (2005). Understanding alternative splicing: towards a cellular code.. Nat Rev Mol Cell Biol.

[B4] Blencowe BJ (2006). Alternative splicing: new insights from global analyses.. Cell.

[B5] Lareau LF, Green RE, Bhatnagar RS, Brenner SE (2004). The evolving roles of alternative splicing.. Curr Opin Struct Biol.

[B6] Lee C, Roy M (2004). Analysis of alternative splicing with microarrays: successes and challenges.. Genome Biol.

[B7] Johnson JM, Castle J, Garrett-Engele P, Kan Z, Loerch PM, Armour CD, Santos R, Schadt EE, Stoughton R, Shoemaker DD (2003). Genome-wide survey of human alternative pre-mRNA splicing with exon junction microarrays.. Science.

[B8] Modrek B, Resch A, Grasso C, Lee C (2001). Genome-wide detection of alternative splicing in expressed sequences of human genes.. Nucleic Acids Res.

[B9] Yeo G, Holste D, Kreiman G, Burge CB (2004). Variation in alternative splicing across human tissues.. Genome Biol.

[B10] Ule J, Ule A, Spencer J, Williams A, Hu JS, Cline M, Wang H, Clark T, Fraser C, Ruggiu M (2005). Nova regulates brain-specific splicing to shape the synapse.. Nat Genet.

[B11] Forch P, Valcarcel J (2003). Splicing regulation in *Drosophila *sex determination.. Prog Mol Subcell Biol.

[B12] Dulac C (2005). Sex and the single splice.. Cell.

[B13] Ladd AN, Cooper TA (2002). Finding signals that regulate alternative splicing in the post-genomic era.. Genome Biol.

[B14] Black DL (2003). Mechanisms of alternative pre-messenger RNA splicing.. Annu Rev Biochem.

[B15] Nakahata S, Kawamoto S (2005). Tissue-dependent isoforms of mammalian Fox-1 homologs are associated with tissue-specific splicing activities.. Nucleic Acids Res.

[B16] Barreau C, Paillard L, Mereau A, Osborne HB (2006). Mammalian CELF/Bruno-like RNA-binding proteins: molecular characteristics and biological functions.. Biochimie.

[B17] Pascual M, Vicente M, Monferrer L, Artero R (2006). The Muscleblind family of proteins: an emerging class of regulators of developmentally programmed alternative splicing.. Differentiation.

[B18] Cooper TA (2005). Use of minigene systems to dissect alternative splicing elements.. Methods.

[B19] Jensen KB, Musunuru K, Lewis HA, Burley SK, Darnell RB (2000). The tetranucleotide UCAY directs the specific recognition of RNA by the Nova K-homology 3 domain.. Proc Natl Acad Sci USA.

[B20] Ule J, Jensen KB, Ruggiu M, Mele A, Ule A, Darnell RB (2003). CLIP identifies Nova-regulated RNA networks in the brain.. Science.

[B21] Sugnet CW, Srinivasan K, Clark TA, O'Brien G, Cline MS, Wang H, Williams A, Kulp D, Blume JE, Haussler D (2006). Unusual intron conservation near tissue-regulated exons found by splicing microarrays.. PLoS Comput Biol.

[B22] Ule J, Stefani G, Mele A, Ruggiu M, Wang X, Taneri B, Gaasterland T, Blencowe BJ, Darnell RB (2006). An RNA map predicting Nova-dependent splicing regulation.. Nature.

[B23] Pan Q, Shai O, Misquitta C, Zhang W, Saltzman AL, Mohammad N, Babak T, Siu H, Hughes TR, Morris QD (2004). Revealing global regulatory features of mammalian alternative splicing using a quantitative microarray platform.. Mol Cell.

[B24] Shai O, Morris QD, Blencowe BJ, Frey BJ (2006). Inferring global levels of alternative splicing isoforms using a generative model of microarray data.. Bioinformatics.

[B25] Pan Q, Saltzman AL, Kim YK, Misquitta C, Shai O, Maquat LE, Frey BJ, Blencowe BJ (2006). Quantitative microarray profiling provides evidence against widespread coupling of alternative splicing with nonsense-mediated mRNA decay to control gene expression.. Genes Dev.

[B26] Kornblihtt AR, de la Mata M, Fededa JP, Munoz MJ, Nogues G (2004). Multiple links between transcription and splicing.. RNA.

[B27] Minovitsky S, Gee SL, Schokrpur S, Dubchak I, Conboy JG (2005). The splicing regulatory element, UGCAUG, is phylogenetically and spatially conserved in introns that flank tissue-specific alternative exons.. Nucleic Acids Res.

[B28] Siepel A, Bejerano G, Pedersen JS, Hinrichs AS, Hou M, Rosenbloom K, Clawson H, Spieth J, Hillier LW, Richards S (2005). Evolutionarily conserved elements in vertebrate, insect, worm, and yeast genomes.. Genome Res.

[B29] Baek D, Green P (2005). Sequence conservation, relative isoform frequencies, and nonsense-mediated decay in evolutionarily conserved alternative splicing.. Proc Natl Acad Sci USA.

[B30] Pan Q, Bakowski MA, Morris Q, Zhang W, Frey BJ, Hughes TR, Blencowe BJ (2005). Alternative splicing of conserved exons is frequently species-specific in human and mouse.. Trends Genet.

[B31] Le K, Mitsouras K, Roy M, Wang Q, Xu Q, Nelson SF, Lee C (2004). Detecting tissue-specific regulation of alternative splicing as a qualitative change in microarray data.. Nucleic Acids Res.

[B32] Xing Y, Resch A, Lee C (2004). The multiassembly problem: reconstructing multiple transcript isoforms from EST fragment mixtures.. Genome Res.

[B33] Fededa JP, Petrillo E, Gelfand MS, Neverov AD, Kadener S, Nogues G, Pelisch F, Baralle FE, Muro AF, Kornblihtt AR (2005). A polar mechanism coordinates different regions of alternative splicing within a single gene.. Mol Cell.

[B34] Bezakova G, Ruegg MA (2003). New insights into the roles of agrin.. Nat Rev Mol Cell Biol.

[B35] Kee Y, Yoo JS, Hazuka CD, Peterson KE, Hsu SC, Scheller RH (1997). Subunit structure of the mammalian exocyst complex.. Proc Natl Acad Sci USA.

[B36] Farhan H, Korkhov VM, Paulitschke V, Dorostkar MM, Scholze P, Kudlacek O, Freissmuth M, Sitte HH (2004). Two discontinuous segments in the carboxyl terminus are required for membrane targeting of the rat gamma-aminobutyric acid transporter-1 (GAT1).. J Biol Chem.

[B37] Gerges NZ, Backos DS, Rupasinghe CN, Spaller MR, Esteban JA (2006). Dual role of the exocyst in AMPA receptor targeting and insertion into the postsynaptic membrane.. EMBO J.

[B38] Keeling SL, Gad JM, Cooper HM (1997). Mouse Neogenin, a DCC-like molecule, has four splice variants and is expressed widely in the adult mouse and during embryogenesis.. Oncogene.

[B39] Matsunaga E, Tauszig-Delamasure S, Monnier PP, Mueller BK, Strittmatter SM, Mehlen P, Chedotal A (2004). RGM and its receptor neogenin regulate neuronal survival.. Nat Cell Biol.

[B40] Martinez-Contreras R, Fisette JF, Nasim FU, Madden R, Cordeau M, Chabot B (2006). Intronic binding sites for hnRNP A/B and hnRNP F/H proteins stimulate pre-mRNA splicing.. PLoS Biol.

[B41] Barash Y, Bejerano G, Friedman N (2001). A simple hyper-geometric approach for discovering putative transcription factor binding sites.. Lecture Notes Computer Sci.

[B42] Ashiya M, Grabowski PJ (1997). A neuron-specific splicing switch mediated by an array of pre-mRNA repressor sites: evidence of a regulatory role for the polypyrimidine tract binding protein and a brain-specific PTB counterpart.. RNA.

[B43] Markovtsov V, Nikolic JM, Goldman JA, Turck CW, Chou MY, Black DL (2000). Cooperative assembly of an hnRNP complex induced by a tissue-specific homolog of polypyrimidine tract binding protein.. Mol Cell Biol.

[B44] Zhang W, Morris QD, Chang R, Shai O, Bakowski MA, Mitsakakis N, Mohammad N, Robinson MD, Zirngibl R, Somogyi E (2004). The functional landscape of mouse gene expression.. J Biol.

[B45] Brown KR, Jurisica I (2005). Online predicted human interaction database.. Bioinformatics.

[B46] Breitkreutz BJ, Stark C, Tyers M (2003). Osprey: a network visualization system.. Genome Biol.

