# Supplementary material for: Functional coordination of alternative splicing in the mammalian central nervous system
Source: Genome Biol. 2007 Jun 12;8(6):R108. doi: 10.1186/gb-2007-8-6-r108 (PMC2394768; doi:10.1186/gb-2007-8-6-r108)
Supplement: Additional data file 1 — Provided is supplemental information on genes with microarray detected tissue specific AS levels, additional details on Materials and Methods, and additional Figures (1-5). [file gb-2007-8-6-r108-S1.pdf]

ADDITIONAL DATA FILE 1:

Functional coordination of alternative splicing in the mammalian central nervous system

Matthew Fagnani[1-3], Yoseph Barash[1,2,4], Joanna Ip[2,3], Christine Misquitta[2], Qun Pan[2], Arneet L. Saltzman[2,3], Ofer Shai[4], Leo Lee[4], Aviad Rozenhek[5], Naveed Mohammad[3], Sandrine Willaime-Morawek[5], Tomas Babak[2,3], Wen Zhang[2,3], Timothy R. Hughes[2,3], Derek van der Kooy[3], Brendan J. Frey[2,4,6] and Benjamin J. Blencowe[2,3,6]

[1] Co-first authors
[6] Co-corresponding authors

[2] Banting and Best Department of Medical Research
[3] Department of Molecular and Medical Genetics
[4] Department of Electrical and Computer Engineering
University of Toronto
[5] Computer Science and Engineering, Hebrew University of Jerusalem,
Jerusalem, Israel 91904

Correspondence:

B.J. Blencowe, PhD
Centre for Cellular and Biomolecular Research
Donnelly CCBR Building
160 College Street
Toronto, Ontario
M5S 3E1
Canada

Tel **416-978-3016**
Fax **416-978-8287**
Email b.blencowe@utoronto.ca
frey@psi.utoronto.ca

SUPPLEMENTAL INFORMATION ON GENES WITH MICROARRAY DETECTED TISSUE-SPECIFIC AS LEVELS

Selected AS events associated with muscle tissues

Several of the genes containing alternative exons that are differentially regulated in muscle-based tissues have known functions in muscle and/or have been implicated in diseases associated with muscle tissues. The largest AS level change among the ~10 muscle-associated AS events was detected in transcripts from the Mitofusin-2 (Mfn2/Fzo) gene, a GTPase involved in mitochondrial fusion that undergoes increased expression in skeletal muscle after exercise, and Mfn2 expression in skeletal muscle may be altered by obesity (Bach et al., 2003; Chen et al., 2003; Cartoni et al., 2005; Neuspiel et al., 2005). This muscle-specific AS event was validated by RT-PCR assays (data not shown). Another large muscle-specific AS change was found in Musashi2/Msi2h, which encodes an RNA-binding protein involved in neural stem cell development (Sakakibara et al. 2001, Sakakibara et al. 2002, Aubert et al. 2003). In another example, we detected a muscle-specific AS difference in transcripts encoding the RNA binding Fox/Fxh/Rbm9 AS factor, which regulates AS in muscle as well as in other tissues (Jin et al., 2003; Nakahata and Kawamoto, 2005; Underwood et al., 2005). Fox-2 contains a previously documented pair of mutually exclusive alternative exons, involving one exon (B40) that is highly included in brain and one exon (M43) that is highly included in muscle (Nakahata and Kawamoto, 2005). Our data indicates that the B40 exon is actually highly included in all tissues except muscle. This suggests that muscle-specific regulation of the B40 exon is important for controlling the function of Fox-2 in muscle tissue.

We found correlated, muscle-associated AS in two exons of Sorbin, the SH3 domain containing 1 (Sorbs1)/c-cbl-associated protein (CAP)/ponsin/SH3P12. Sorbs1 is involved in insulin receptor signalling (Ribon et al. 1998a, Baumann et al. 2000, Kimura et al. 2001, Mitra et al. 2003), and it also functions in cell adhesion (Ribon et al. 1998b, Mandai et al. 1999). Mutations in Sorbs1 have been associated with obesity and type 2 diabetes (Lin et al. 2001, Yang et al. 2003). Sorbs1 mRNA has complex AS isoform patterns with widely varying tissue distributions (Mandai et al. 1999, Lebre et al. 2001, Lin et al. 2001, Zhang et al. 2003, Alcazar et al. 2004, Matson et al. 2005). One exon (25) of mouse Sorbs1/CAP has been found to be muscle-specific (Zhang et al. 2003), and this exon does not correspond to the two exons (5th and $\sim 13^{th}$) we observe to have muscle-specific AS changes.

Protein tyrosine phosphatase-like member a (Ptpla) has been found to be most highly expressed in mouse muscle tissues (Uwanogho et al. 1999, Li et al. 2000). A tRNA-like SINE exonic insertion in the dog Ptpla gene results in splicing defects that result in autosomal recessive centronuclear myopathy (Pele et al. 2005). We observed muscle-related AS changes in exon 5 of Ptpla, and two isoforms of the orthologous exon 5 of dog Ptpla included and excluded were found in skeletal muscle (Pele et al. 2005). Sarcolemma-associated protein (Slmap) is a sarcoplasmic membrane protein that is a component of the centrosome (Wigle et al. 1997, Wielowieyski et al. 2000, Guzzo et al. 2004). Slmap has been associated with muscle excitation and contraction (Guzzo et al. 2004), and deregulated expression of the shortest splice isoform of Slmap correlates with endothelial dysfunction in type 2 diabetic mice (Ding et al. 2005). Exclusion of two conserved alternative exons from Slmap in rabbit muscle tissues was previously described (Wielowieyski et al. 2000), and we observed the muscle-associated exclusion of one of these exons (referred to as exon IV in Wielowieyski et al. 2000).

We found ~5 cases in which there appeared to be an AS pattern common to CNS and muscle tissues. One

such gene is Sorting nexin-14 (Snx14), a gene expressed in the mouse nervous system (Carroll et al. 2001). Snx14 is a member of the sorting nexin family which is known to be involved in phospholipid binding and endocytosis (Worby et al. 2003). Requiem/Ubi-d4/Dpf2 is a d4-zinc finger domain-containing transcription factor involved in apoptosis (Gabig et al. 1994, Chestkov et al. 1996, Gabig et al. 1998, Mertsalov et al. 2000). Alternative splicing of Requiem/Ubi-d4/Dpf2 does not appear to have been previously described, and our data leads us to suggest a potential common AS regulation of Requiem/Ubi-d4/Dpf2 in neural and muscle tissues. A couple of uncharacterized genes (4632411B21Rik and R3Dhm2/1300003K24Rik) with common muscle and CNS AS patterns were also observed.

Embryonic-specific AS events

We observed embryonic-specific AS patterns for several genes. Drebrin (Dbn1) is associated with the cytoskeleton and cell-cell junctions (Shirao 1995, Peitsch et al. 1999, Butkevich et al. 2004). Dbn1 has known functions in embryonic brain development (Kojima et al. 1988, Kojima et al. 1993, Shirao 1995, Mizui et al. 2005), adult neurons (Shirao et al. 1992, Jin et al. 2002, Kobayashi et al. 2001, Shim and Lubec 2002), stomach, and kidney (Peitsch et al. 1999, Keon et al. 2000, Peitsch et al. 2003). Exon 11a of mouse Dbn1 was found to be excluded specifically in embryonic brain and included in adult brain (Jin et al. 2002); embryonic and adult brain specific splice isoforms have also been found in chicken (Kojima et al. 1993). Consistent with these results, we find that exon 11a highly excluded in embryonic tissues. Paralemmin (Palm) is a plasma membrane-anchored protein with known roles in embryonic and adult brain and eye (Burwinkel et al. 1998, Kutzleb et al. 1998, Bagchi et al. 2003, Castellini et al. 2005, Basile et al. 2006). Exon 8 of Paralemmin has been previously found to be increasingly included during the progression of mouse brain and eye development (Kutzleb et al. 1998, Castellini et al. 2005), and we observe a large increase in exon 8 inclusion in adult tissues relative to the embryonic tissues. SC35 is a pre-mRNA splicing factor of the SR protein family (Xu and Maniatis 1992, Sureau and Perbal 1994). SC35 autoregulates the alternative splicing of its pre-mRNA (Sureau et al. 2001). We observed an embryonic-specific change in splicing of SC35, consistent with previous observations that SC35 isoform levels have been observed in uterus and embryos during pregnancy (Nie et al. 2000, Nie et al. 2002, Salamonsen et al. 2002).

The proto-oncogene Ect2 encodes a RhoGEF that regulates cytokinesis in mammals (Miki et al. 1993, Takai et al. 1998, Tatsumoto et al. 1999, Kimura et al. 2000, Saito et al. 2003, Saito et al. 2004, Liu et al. 2004) and Drosophila (Prokopenko et al. 1999, Somers and Saint 2003, Schumacher et al. 2004, Smallhorn et al. 2004). We observed an embryonic-dependent change in AS of Ect2, and potential tissue-specific isoforms of Ect2 do not appear to have been previously described. DNA polymerase alpha subunit B (Polr2) is required for DNA replication in all eukaryotes (Collins et al. 1993, Miyazawa et al. 1993, Mizuno et al. 1998, Mizuno et al. 1999, Nishikawa et al. 2000). An embryonic-associated AS change in exon 2 of Polr2 was observed; however, transcript levels from this gene appear to be much higher in embryonic versus other tissues. Embryonic-related AS changes were also found in uncharacterized genes including. Transmembrane protein 39 A (Tmem39a).

CNS-regulated AS events in genes associated with signalling pathways

Many of the genes found to undergo CNS-specific AS in our study act in signalling pathways. In the interest of brevity, only the known or putative functions of these genes in the nervous system, as described in the published literature, are summarized below. Several of the CNS-specific alternative exons belong to genes involved in the Rho/Rac/Cdc42, Rap, MAPK, and CAMKII signalling pathways. Significant CNS-specific AS changes were detected in four members of the Rap1 pathway (Rap1ga1,

Rap1gds1, Rap1gef4, Rap1gef6). Alternative isoforms of human Rap1GAP1 are important in neurite outgrowth (Jordan et al., 2005). Rapgef6 has been linked to both neurogenesis and gliogenesis (Kempermann et al., 2006). Three alternative isoforms of human Rapgef6/PDZ-GEF2/RA-GEF2 have been studied, and the orthologous exon that we detected as having a neural-specific AS change appears to distinguish the highly expressed full-length PDZ-GEF2A (which we find to be more included in CNS) and the low abundance C-terminally truncated PDZ-GEF2B isoforms (Gao et al., 2001; Kuiperij et al., 2003).

We detect a CNS-specific AS event in transcripts of the Rap1 GTP-GDP dissociation stimulator 1 (Rap1gds1)/Smg (p21)/GDS1. This protein activates the Rho, Ras, and Rap1 GTPases and has been found to act in cell survival pathways (Kaibuchi et al., 1991; Mizuno et al., 1991; Shirataki et al., 1991; Kotani et al., 1992; Orita et al., 1993; Rebhun et al., 2000; Takakura et al., 2000). Rap1gds1 does not appear to have a known neural function, and to our knowledge only an uncharacterized variant of Rap1gds1, detected in bovine brain, has been reported (Kaibuchi et al., 1991). CNS-specific AS level differences were detected in three mitogen-activated protein kinase (MAPK) pathway components (Map3k4, Map4k6 and Mapkapk3). Mouse Map3k4/MEKK4/MAPKKK4 mutants have neural tube development defects (Abell et al., 2005; Chi et al., 2005). Our data indicates that exon 16 of Map3k4/MEKK4 is more highly included in CNS tissues relative to other tissues. Alternative isoforms of MEKK4alpha and MEKK4beta which, respectively, contain or lack this exon, have been reported but their splicing patterns were not noted to differ across eight mouse tissues that were analyzed (Gerwins et al., 1997). Mouse Map4k6/Misshapen/NIKs-related kinase (MINK) is upregulated in the postnatal brain, and its *C. elegans* ortholog, mig-15, is required for correct axon guidance (Dan et al., 2000; Poinat et al., 2002). Human Map4k6/hMINK has been reported to have at least five splice isoforms, and the exon that we observe to be more highly excluded in CNS tissues appears to be orthologous to an exon previously reported to be excluded in the hMINKgamma (primarily brain) and hMINKdelta (primarily muscle) isoforms (Hu et al., 2004).

The calcium/calmodulin-dependent protein kinase (CaMK) pathway is known to be regulated by alternative splicing (Bayer et al., 2002; Xu et al., 2005). We find that two members (Camk2d and Camk2g) of the CaMK pathway undergo AS in a CNS-specific manner. The role of CaMKII in the nervous system has been well studied (Colbran and Brown, 2004). . Camk2d is alternatively spliced during neural development (Donai et al., 2000), and both Camk2d and Camk2g have astrocyte-specific alternative isoforms (Vallano et al., 2000). CaMKII phosphorylates PSD-95/Dlgh4 and dynamin/Dnm1 (two other genes with neural-specific AS discussed below) in the postsynaptic density of rats (Yoshimura et al., 2000).

Arhgef7/betaPIX, GIT2, and Scribbled are signalling genes, that we detect to undergo CNS-specific AS. Arhgef7/betaPIX is a Rho family (Rho, Rac, and Cdc42) guanine exchange factor that regulates dendritic spine morphogenesis and the synapse formation in hippocampal neurons in a pathway dependent on the GIT2 homolog GIT1 (Zhang et al., 2005). The CNS-specific event we observed in Arhgef7/betaPix appears to be the 177-bp exon that is included in the betaPIX-b isoform found in brain and testes (Kim et al., 2000). GIT2 is an ADP-ribosylation GTPase-activating protein that undergoes complex AS patterns, and GIT2 is known to interact with Arhgef7/betaPIX and the postsynaptic cytomatrix protein Piccolo (Premont et al., 2000; Kim et al., 2003). Scribbled is necessary for neural tube development in mice (Murdoch et al., 2003), and human Scribbled forms a complex with Arhgef7/betaPIX and GIT1 (Audebert et al., 2004; Lahuna et al., 2005). Thus, the neural-specific function of the Scribbled/betaPIX

complex is likely regulated by AS. Arhgef7/betaPIX also interacts with the POPX2/FEM-2/Protein phosphatase 1F/Ppm1f (Koh et al. 2002), and Ppm1f contains another CNS-specific AS event. In addition, POPX2/FEM-2/Ppm1f interacts with Calcium/Calmodulin-dependent kinase II/CaMKII (Tan et al. 2001, Harvey et al. 2004). Other Rho family signalling proteins found to be associated with CNS-specific AS based on our data are IQGAP1,Vav2, Dock9/Zizimin1 and Dock10/Zizimin3. Vav2 has been implicated in axon guidance and neurite outgrowth (Aoki et al., 2005; Cowan et al., 2005).

We detect CNS-associated AS events in transcripts of two related Rho family GEFs, Dock9/Zizimin1 and Dock10/Zizimin3, which have not yet been found to have a neural-related function (Cote and Vuori, 2002; Meller et al., 2002; Meller et al., 2004; Meller et al., 2005; Nishikimi et al., 2005). A large CNS-specific difference in AS was detected for two exons of Dock9/Zizimin1, which has been noted previously to have five alternatively spliced isoforms that are conserved between mouse and human (Nishikimi et al., 2005). Although Dock9/Zizimin1 and Dock10/Zizimin3 were found to be expressed in several mouse tissues (including brain), the tissue distributions of the various splice isoforms of this gene were not determined (Nishikimi et al., 2005). Based on our results it is possible to speculate that certain splice forms of Dock9/Zizimin1 and Dock10/Zizimin3 have neural-specific functions.

A CNS-specific AS event was detected in phospholipase C-beta 4/Plcb4, a protein that is involved in the metabotropic glutamate neurotransmitter signalling pathway and synaptic depression in the cerebellum (Kim et al., 1997a; Hashimoto et al., 2001; Hirono et al., 2001; Miyata et al., 2001; Miyata et al., 2003). Alternative splicing of Plcb4 transcripts was previously shown to be regulated by Nova-2 (Ule et al., 2005).

We detect CNS tissue-specific AS of transcripts corresponding to three protein tyrosine phosphatases, including two events in Ptprf, one in Ptprk, and one in LMW-PTP/Acp1. Neural-specific AS of the Ptprf/LAR receptor protein phosphatase has been previously reported (Zhang and Longo, 1995; Honkaniemi et al., 1998; Zhang et al., 1998). Ptprf/LAR functions in excitatory synapse development (Van Lieshout et al., 2001; Dunah et al., 2005). Ptprk has been found to stimulate neurite growth via the MAPK pathway (Drosopoulos et al., 1999).

OPA1 is a dynamin-related GTPase that regulates mitochondrial fusion and morphology (Cipolat et al., 2004; Griparic et al., 2004), and mutations in OPA1 result in the hereditary neuropathy Autosomal dominant optic atrophy 1 (Alexander et al., 2000; Delettre et al., 2000). A distinct mRNA isoform of mouse OPA1, with enriched expression in forebrain, cerebellum and brain stem, relative to other tissues, has been reported, but the specific exon(s) accounting for these different isoforms was not determined (Misaka et al., 2002).

Our observations lead us to conclude that AS regulates multiple components of Rap, Rho, MAPK and CAMK signalling pathways in a nervous system-specific manner. In particular, GTPase-activating proteins and guanine exchange factors appear to be highly regulated by AS in the CNS.

CNS-regulated AS in genes associated with cytoskeletal functions.

We detected an overrepresentation of CNS-specific splicing events in transcripts that are known to be involved in cytoskeletal-associated functions. Two CNS-specific AS events were detected in transcripts of the actin-binding LIM protein 1 (Ablim1) gene, which is a component of the actin cytoskeleton (Roof et al., 1997) and regulates axon guidance (Erkman et al., 2000; Lu et al., 2003). The *C. elegans* ortholog

of Ablim1, UNC-115, is an effector of the Rac signalling pathway and is associated with the actin cytoskeleton during axon pathfinding, and the gene controls neuronal morphogenesis (Wightman et al., 1997; Lundquist et al., 1998; Struckhoff and Lundquist, 2003; Yang and Lundquist, 2005). We also detect a CNS-specific AS event in transcripts of Dystonin/BPAG1. This protein connects the actin and microtubule-based cytoskeletons (Yang et al., 1996; Yang et al., 1999), and is involved in the neurodegenerative-disorder Dystonia musculorum (Brown et al., 1995), nervous system development (Guo et al., 1995), myelination (Saulnier et al., 2002), and retrograde axonal transport (Liu et al., 2003). Neural-specific splice variants of Dystonin/BPAG1 transcripts have been reported (Leung et al., 2001; Okumura et al., 2002), although they do not correspond to the CNS-specific AS event we have identified. Transcripts of the synaptic nuclear envelope 1 (Syne-1/Nesprin-1/CPG2) gene, which encodes an actin-binding protein that anchors the synaptic nuclear envelope to the cytoskeleton at neuromuscular junctions (Apel et al., 2000; Zhang et al., 2002; Grady et al., 2005), were detected to undergo CNS-specific AS. This observation is consistent with the previous finding of a brain/synapse-specific splice isoform (CPG2) which regulates endocytosis of glutamate receptors (Cottrell et al., 2004).

We detect CNS-specific AS of Tropomodulin 2 (Tmod2) transcripts. Tmod2 is an actin filament capping protein (Cox and Zoghbi, 2000). Mice without Tmod2 have learning and memory deficits and enhanced long-term potentiation (Cox et al., 2003). The rat ortholog, N-tropomodulin, is known to bind to brain tropomyosin (Watakabe et al., 1996). We observe CNS-specific splicing of Alpha-spectrin 2 (Spna2) exon 4; a similar change in the inclusion level was also noted by Ule et al. (2005). Spna2 binds to actin cytoskeleton components in a neural-specific manner (Clark et al., 1994; Gelot et al., 1994; Schmitz, 2001), and Spna2 has been found in complex with the neurotransmitter receptor 5-HT(2C) (Becamel et al., 2002).

Dematin/Epb4.9 is an actin and spectrin interacting cytoskeletal protein required for erythroid membrane stability (Rana et al., 1993; Azim et al., 1995; Azim et al., 1996; Azim et al., 1999; Khanna et al., 2002; Frank et al., 2004). It is also known to modulate MAPK pathways (Lutchman et al., 2002). Two brain-specific splice isoforms of human dematin/Epb4.9 have been identified (Kim et al., 1998), one of which corresponds to a highly significant CNS-specific AS event we have observed. These findings suggest a currently unknown neural-specific function for dematin/Epb4.9.

We detected CNS-specific AS in transcripts encoding two myosins, Myo5a (which has a pair of positively correlated exons) and Myo6. Myosin Va is an actin-based processive motor that transports synaptic vesicles in axons (Prekeris and Terrian, 1997; Bridgman, 1999; Naisbitt et al., 2000; Libby et al., 2004; Watanabe et al., 2005). Myo6 functions in AMPA/glutamate receptor endocytosis, and Myo6 deficient mice have abnormal neurons and astroglia (Wu et al., 2002; Osterweil et al., 2005). Kinesins are molecular motors that move cargo along microtubules, and they are important in directional transport in axons and dendrites (Hirokawa and Takemura, 2005). We observe CNS specific AS of an exon in transcripts of kinesin-superfamily associated protein 3 (Kifap3/KAP3/SMAP), a component of the KIF3 complex involved in axonal vesicle transport during the processes of neurite growth and neuroepithelial development (Yamazaki et al., 1996; Takeda et al., 2000; Teng et al., 2005). Additionally, human KAP3/SMAP interacts with components of the Rap1 and MAPK signaling pathways, including SmgGDS/Rap1gds1, which we observe to also have a CNS-specific AS event (see Table 1 and Additional Data File 3) (Shimizu et al., 1996; Nagata et al., 1998). We also find that exon 9 of transcripts of the microtubule-associated gene CLASP1 undergoes neural-specific splicing, and this finding is consistent with the previous observation that this exon is regulated by Nova-2 (Ule et al., 2005). The

Drosophila and vertebrate orthologs of CLASP1 are involved in axon guidance (Lee et al., 2004). In summary, our results reveal that the transcripts of multiple actin, myosin and microtubule-based cytoskeletal components are regulated in a CNS-specific manner by AS.

CNS-regulated AS events in genes associated with vesicle transport

Vesicle-mediated transport is essential in many neural processes including the transport of neurotransmitters to the synapse (Li and Chin, 2003). We observed neural-specific splicing of transcripts of several genes encoding vesicular transport proteins. Two exons of ADP-ribosylation factor (ARF) GTPase activating protein-1 (ArfGAP1), which regulates COPI vesicles during ER-Golgi transport, appear to undergo significant negatively-correlated AS, and the exons have distinct inclusion levels in CNS tissues. This pair of ArfGAP1 exons has recently been shown to be spliced in a brain-specific manner in rat with the same pattern we observe (Parnis et al., 2006).

We detect a large CNS tissue-specific difference in the AS levels of transcripts encoding Dynamin 1 (Dnm1), a GTPase necessary for synaptic vesicle endocytosis (Smillie and Cousin, 2005; Yamashita et al., 2005). Eight splice isoforms of Dnm1 have been identified in rat tissues (Cao et al., 1998). Dynamin has also been linked to the actin cytoskeleton (Orth and McNiven, 2003; Schafer, 2004). Thus, ArfGAP1 and Dynamin 1 are GTPases involved in vesicular transport regulated by AS. The Rab GTPase interacting proteins Rab6ip2/ERC1/CAST2 and Rab3ip/Rabin3/Rabin8 show large nervous system-associated differential AS levels. Rat Rab6ip2/ERC1/CAST2/ELKS interacts with presynaptic active zone RIM (Rab3-interacting molecule) proteins to regulate neurotransmitter release in an AS regulated process (Wang et al., 2002). Rab6ip2 binds Rab6 GTPase, which is involved in the endosome-Trans Golgi Network vesicular transport pathway (Monier et al., 2002). We observe significant negative correlation between two alternative exons in Rab6ip2; one of these exons is orthologous to the human Rab6ip2/ERC1b brain-specific exon (Wang et al., 2002), while the other exon is orthologous to exon 14a of human ELKS/Rab6ip2, which was found to be more highly excluded in brain (Nakata et al., 2002; Deguchi-Tawarada et al., 2004). Transcripts of Rab3ip/Rabin3/Rabin8, a Rab8-GEF and Rab3A binding protein involved in actin remodeling and polarized vesicular transport, were found to have a CNS-specific AS event (Brondyk et al., 1995; Hattula et al., 2002). Alternative splicing of Rab3ip/Rabin3/Rabin8 does not appear to have been described previously. Therefore, some of the Rab GTPase vesicular transport pathways could be regulated by AS of Rab6ip2/ERC1/CAST2, and Rab3ip/Rabin3/Rabin8.

The syndapin II/PACSIN2 protein is involved in receptor mediated endocytosis and actin cytoskeleton organization, and the related syndapin I protein is involved in synaptic vesicle endocytosis (Ritter et al., 1999; Modregger et al., 2000; Qualmann and Kelly, 2000; Kessels and Qualmann, 2002; Kessels and Qualmann, 2004). We detect a CNS tissue-associated AS event in syndapin II, and this suggests a possible nervous system-specific function for this protein. Synaptosomal associated protein 23 (SNAP-23) is a t-SNARE involved in vesicle membrane fusion during exocytosis in many tissues; in neurons, SNAP-23 releases glutamate receptors to the cell surface (Washbourne et al., 2004; Fournier and Robinson, 2006). Nervous system-associated exclusion level changes are observed for two exons in SNAP-23 transcripts. Five splicing isoforms of the human SNAP-23 have been previously described, but the tissue specificity of these isoforms was not established (Shukla et al., 2001). SNAP-23 and Dynamin 1 have been found to be part of a membrane-bound protein complex involved in endocytic vesicular transport (Predescu et al., 2003).

Significant positive correlation of AS levels for two exons in Exoc7/Exo70 transcripts (Figure 2) was observed, and both exons display neural specific AS level differences. Exoc7/Exo70 is a component of the exocyst complex involved in vesicle-mediated exocytosis (Kee et al., 1997), and it is directly involved in the membrane targeting of the neurotransmitter receptors for GABA and NMDA (Farhan et al., 2004; Gerges et al., 2006). Highly significant CNS-specific AS levels were observed in an exon of Postsynaptic-density 95(PSD-95)/Discs, large homolog 4 (Dlgh4). PSD-95 is a well-studied protein involved in synaptic vesicle maturation (reviewed by (Hata and Takai, 1999; Kim and Sheng, 2004), and multiple splice isoforms have been observed (Bence et al., 2005).

We detect a CNS-specific AS event in Src homology 3-domain growth factor receptor-bound 2-like (endophilin)interacting protein 1 (Sgip1). Sgip1 regulates energy balance in the rat hypothalamus (Trevaskis et al., 2005), and is known to interact with endophilins, which are proteins involved in synaptic vesicle endocytosis (Trevaskis et al., 2005). Synaptogyrin 1 (Syngr1) is a synaptic vesicle component with roles in synaptic plasticity and Ca2+-dependent exocytosis (Stenius et al., 1995; Kedra et al., 1998; Janz et al., 1999; Sugita et al., 1999; Belfort and Kandror, 2003), and mutations in Syngr1 have been associated with schizophrenia and bipolar disorder (Verma et al., 2004; Verma et al., 2005). Human Syngr1 has an alternatively spliced form that is highly expressed in brain (Kedra et al., 1998), and the exon associated with this isoform may be orthologous to the mouse alternative exon we detect as having CNS-specific AS. Collectively, the CNS-specific AS patterns described above lead us to conclude that vesicle-mediated transport is highly regulated by AS in the nervous system.

CNS-specific AS events in genes with known neural functions

We detect a CNS-specific AS event in transcripts encoding adenosine deaminase ADARB1/ADAR2, an RNA editing enzyme that alters glutamate receptor mRNAs (Lai et al., 1997; Yang et al., 1997; Higuchi et al., 2000). The AS of ADARB1/ADAR2 is auto-regulated by RNA editing (Rueter et al., 1999; Slavov and Gardiner, 2002; Kawahara et al., 2005). The CNS-regulated exon (referred to as exon 1b) in Adarb1 is not conserved in human. Insertion of exon 1b results in a frameshift that potentially results in expression of a truncated protein (Slavov and Gardiner, 2002). Expression of exon 1b was previously surveyed only in brain and liver (Slavov and Gardiner, 2002). We observed reduced inclusion levels of exon 1b specifically in CNS tissues, which suggests a possible role for this exon in controlling the CNS-specific activity of Adarb1.

Amyloid beta (A4) precursor protein-binding, family B, member 1 (Appb1)/Fe65 is a ligand of beta-amyloid precursor, which is involved in Alzheimer's disease (Sabo et al., 2003; Wang et al., 2004a). Human Fe65 transcripts undergo neural-specific splicing of exon 9 (Hu et al., 1999); we detect a CNS-specific AS event involving what appears to be a rarely-included cassette exon between the first and second constitutive exons of the mouse Fe65 gene. Membrane associated guanylate kinase inverted 1 (MAGI-1)/BAI1-associated protein 1 (BAIAP1) is a tight-junction protein that is involved in Delta-Notch and Rap1 signalling in the nervous system (Dobrosotskaya et al., 1997; Shiratsuchi et al., 1998; Mino et al., 2000; Wright et al., 2004; Mizuhara et al., 2005; Sakurai et al., 2006). We detect CNS-tissue specific AS of two exons in MAGI-1 transcripts (exons 15, ~20). Brain-specific AS has previously been observed for human and mouse exon 15 (Laura et al., 2002; Sugnet et al., 2006). MAGI-3 is a paralog of MAGI-1 that has also been implicated in nervous system Delta-Notch signalling, and MAGI-3 binds beta1-adrenergic receptor thereby inhibiting ERK/MAPK signalling (Wu et al., 2000; Wright et al., 2004; He et al., 2006). We observe CNS tissue-specific AS of the penultimate exon in MAGI-3, which was also found to be brain-specific by Sugnet et al. (2006).

An AS event displaying a large CNS-specific exon inclusion level difference was observed in transcripts of the Tight junction protein 4 (Tjp4/Pilt), and human Pilt is known to interact with the synapse associated protein 97/hDlg1 (Kawabe et al., 2001). The Kinase D-interacting substrate of 220 kDa (Kidins220)/ankyrin repeat-rich membrane spanning protein (ARMS)/C330002I19Rik functions in neuronal signalling pathways involving neurotrophin and ephrin receptor tyrosine kinase, Rap1-mediated MAPK and protein kinase C/D pathways (Iglesias et al., 2000; Kong et al., 2001; Arevalo et al., 2004; Cabrera-Poch et al., 2004; Chang et al., 2004; Luo et al., 2005; Arevalo et al., 2006).

Transcripts encoding a meningioma-expressed antigen (MGEA6/MEA6/CTAGE5) have been reported previously to undergo tumor-specific AS (Heckel et al., 1997; Comtesse et al., 2002; Usener et al., 2003). We detect a CNS-specific AS event in MGEA6 transcripts. We also detect a pronounced CNS-specific differential AS event in transcripts encoding mahoganoid/mahogunin ring finger 1 (Mgrn1). Homozygous Mgrn1 knockout mice show neuronal degeneration and astrocytosis phenotypes (He et al., 2003). Mgrn1 has E3-ubiquitin ligase activity and this suggests that it may have a role in proteolysis (He et al., 2003).

We detect CNS-specific AS of exon 11 (referred to as EIII) in transcripts of Meis2/Mrg1, a homeodomain transcription factor involved in mouse brain development and Meis2 is known to regulate the transcription of the dopamine D1 receptor gene in adult brain tissues (Cecconi et al., 1997; Oulad-Abdelghani et al., 1997; Toresson et al., 2000; Yang et al., 2000). The *C. elegans* ortholog of Meis2 (unc-63) is required for neuroblast migration (Yang et al., 2005), while the *Drosophila* ortholog is involved in axonal patterning (Nagao et al., 2000). Four splice isoforms of mouse Meis2 are known: Meis2a and Meis2b, which include the EIII exon, are most abundant in brain, and Meis2c and Meis2d, which exclude the EIII exon, and are most abundant in female genital organs (Oulad-Abdelghani et al., 1997). Consistent with these previous findings, our data indicate that the EIII exon is more highly included in the nervous system, as compared to the profiled other tissues.

Neogenin (Neo1) is a cell-surface receptor involved in axon guidance that mediates the effect of the repulsive guidance molecule, and is also known to regulate neuron survival in vertebrates (Vielmetter et al., 1994; Gad et al., 1997; Meyerhardt et al., 1997; Matsunaga et al., 2004; Rajagopalan et al., 2004). We detect strong negative correlation between the AS levels of two alternative exons in Neo1 transcripts (see Figure 2). One of these is the fourth alternative exon (Exon ~21), which overlaps the coding region of the cytoplasmic domain of the protein, and this exon has been shown previously to be regulated during mouse embryogenesis (Keeling et al., 1997).

NFATc3/NFATx/NFAT4 is a calcineurin-regulated transcription factor that regulates axon outgrowth, neuronal apoptosis and astrocyte function (Graef et al., 2003; Jones et al., 2003; Jayanthi et al., 2005). Four splice isoforms of human NFATc3/NFATx/NFAT4 have been identified and shown to be expressed at variable levels across tissues. An orthologous isoform (NFATx2) has also been identified although its tissue distribution was not well characterized (Imamura et al., 1998). We detect CNS-specific inclusion of an alternative exon in mouse NFATx2; the NFATx1 isoform lacks this alternative exon (Liu et al., 1997).

The purinergic receptor P2X4 regulates presynaptic glutamatergic transmission in neurons in a dynamin-dependent endocytic process, and studies using rat nerve injury models have shown that P2X4 mediates

allodynia in spinal microglia (Bobanovic et al., 2002; Tsuda et al., 2003; Ashour and Deuchars, 2004; Schwab et al., 2005). We detect CNS-specific AS of a cassette exon that was previously reported to differ between splice isoforms of P2X4 (P2X4 and P2X4a; (Townsend-Nicholson et al., 1999). The distribution of these isoforms was only studied in brain in this previous study, and evidence has also been presented suggesting that human P2X4 is alternatively spliced in a brain subregion-specific manner (Carpenter et al., 1999) .

In agreement with a previous study (Zhao and Manley, 1996), we find that a splice isoform of RNA poly(A) polymerase/Papola named PAP IV is enriched in brain tissues. This alternative isoform of PAP may be involved in polyadenylation at synapses (Huang et al., 2002).

We detect a CNS-specific AS event in transcripts of Protein-L-isoaspartate (D-aspartate) O-methyltransferase 1 (Pcmt1). Mice deficient in Pcmt1 die from seizures, and have impaired spatial memory and synaptic function (Kim et al., 1997b; Yamamoto et al., 1998; Kim et al., 1999; Ikegaya et al., 2001). Consistent with our data, Pcmt1 has been reported to have testis and brain specific isoforms (Galus et al., 1994; Mizobuchi et al., 1994; Ogawara et al., 2002).

Mutation of the polyglutamine tract of Ataxin-2/Sca2 results in the neurodegenerative disease spinocerebellar ataxia 2 (Imbert et al., 1996; Pulst et al., 1996; Sanpei et al., 1996; Huynh et al., 2000). We observe that exon 21 of Sca2 transcripts is included at higher levels in CNS tissues, compared to the other microarray-profiled tissues. The tissue specificity of this AS event was not apparent from a previous study which surveyed only five mouse tissues (Affaitati et al., 2001).

Hsp47/Serpinh is a collagen-binding protein with a putative role in nervous system development and glial cell protection (Walsh et al., 1997; Acarin et al., 2002). Our data indicate that two adjacent exons in Hsp47 transcripts display a positively correlated CNS-specific pattern. AS of these exons was previously shown to induced by heat shock (Takechi et al., 1994).

We detect a previously unreported CNS-specific AS event in transcripts encoding the potassium-dependent sodium/calcium ion exchanger NCKX2/Slc24a2, which is involved in mediating calcium ion clearance in axon terminals (Tsoi et al., 1998; Dong et al., 2001; Lee et al., 2002; Sergeeva et al., 2004).

The mouse transcription factor Tcf12/HEB/ME1/REB/ALF1/HTF4 has an expression profile indicative of a role in nervous system development and neuronal plasticity (Neuman et al., 1993; Chiaramello et al., 1995; Uittenbogaard and Chiaramello, 1999; Uittenbogaard and Chiaramello, 2002), and its ortholog in *Drosophila* (*daughterless*) is involved in neural precursor development (Caudy et al., 1988; Vaessin et al., 1994; Hassan and Vaessin, 1997; Ramain et al., 2000). In agreement with previous observations (Nielsen et al., 1992), we detect CNS-specific AS of an alternative exon in Tcf12 transcripts.

We detect a CNS-specific AS event in transcripts of the uncharacterized mouse gene 1810044A24Rik, an ortholog the human protein NIK and IKK-beta-binding protein (NIBP). NIBP was recently shown to be involved in NF-kappaB and nerve growth factor pathways, and siRNA knockdown of NIBP prevents neurite extension (Hu et al., 2005).

We detect a CNS-specific AS event in transcripts of the F-box protein 25 (Fbxo25), which is involved in protein ubiquitination, and Fbxo25 has been found to be disrupted in a patient with mental retardation

and epilepsy (Hagens et al., 2006; Maragno et al., 2006). Alternative exons have been identified in human and mouse Fbxo25, but the tissue distributions of the alternative splice isoforms of this gene were not determined in detail (Hagens et al., 2006).

CNS-specific AS events in genes without known neural functions or genes that are not well characterized

We observed many cases of CNS-specific AS changes in mRNAs of uncharacterized genes or genes without a known neural function. A core component of the cytoplasmic mRNA decay sites, referred to as GW/P-bodies, GW182/Tnrcc6a (Eystathioy et al. 2002, Yang et al. 2004), shows a large CNS-specific AS change in one exon. Human GW182 has recently been identified as being an important part of microRNA-based mRNA decay and RNA interference in GW/P-bodies (Liu et al. 2005A, Liu et al. 2005B, Jakymiw et al. 2005, Lian et al. 2006). GW182/Tnrcc6a was originally noted for containing a large number of CAG repeats that are often found in genes associated with neurological disorders (Margolis et al. 1997), and GW182 was identified as an autoantigen from a patient with sensory ataxic neuropathy (Eystathioy et al. 2003). Two isoforms of human GW182 differing by exon 10 (likely the exon we observed) have been described, but their tissue distribution was not determined (Eystathioy et al. 2002). Thus, the observed AS change may reflect an specific need requirement for GW182-mediated mRNA decay in neural tissues.

Target of myb-like 2 (Tom1l2) and its homologs have recently been found to be involved with clathrin recruitment to endosomes and Src signalling (Katoh et al. 2006, Puertollano 2005, Franco et al. 2006). The large CNS-specific AS change we detect in Tom1l2 transcripts leads us to suggest a potential function of Tom1l2 in synaptic vesicle endocytosis.

We found a large AS change in HIV-Rev binding protein (Hrb)/RIP, an ArfGAP domain-containing that in vertebrates is implicated primarily in sperm formation (Kang-Decker et al. 2001, Juneja 2005) and nuclear export of HIV mRNA (Fritz et al. 1995, Fritz et al. 1996, Bogerd et al. 1995, Fridell et al. 1996, Sanchez-Velar et al. 2004). The Drosophila ortholog of Hrb, Drongo, has been implicated in both neurogenesis and oogenesis (Pritchard et al. 1997). In addition, Hrb interacts with Eps15 (Doria et al. 1999), and Eps15 is a clathrin receptor-mediated endocytic protein that has been implicated in synaptic vesicle recycling, nerve terminal stimulation, and glutamate receptor endocytosis (Chen et al. 1999, Salcini et al. 2001, Pula et al. 2004). We suggest that this combined evidence supports the hypothesis that mouse Hrb/RIP may be involved in nervous system function through endocytosis in an AS-regulated manner.

The uncharacterized gene Claudin containing domain 1 (Cldnd1)/1110019C08Rik has a human ortholog (C3orf4/Cldnd1) that has been found to have distinctive expression patterns in the myelin-producing regions of the brain, and it contains a domain that is present in a peripheral myelin protein (PMP-22/EMP/MP20/Claudin domain) (Fayein et al. 2002). Together with the detection of the large CNS-specific AS change that we observe, the evidence suggests a potential function of Cldnd1 in the nervous system.

A CNS-specific AS change was also detected in Polybromo 1 (Pb1)/BAF180 transcripts. We found correlated CNS-related changes in the splicing of two exons in Pb1/BAF180, and multiple alternatively splice isoforms have been identified previously in the human ortholog (Horikawa et al. 2002). Our

findings lead us to speculate about a potential neural-specific function for the PBAF/SWI/SNF-B complex; this possibility is supported by the existence of a distinct but related neural-specific chromatin remodeling complex called bBAF (Olave et al. 2002).

OS-9/4632413K17Rik binds to N-copine, which is involved in synaptic plasticity in a calcium-dependent manner (Nakayama et al. 1999). An isoform of mouse OS-9, which includes an alternative exon that we detect to have a CNS-specific AS change, was previously found to bind to meprin-beta, while the isoform with the exon excluded was shown not bind to meprin-beta (Litovchick et al. 2002).

Acbd4 and Acbd5 are two highly homologous proteins that contain acyl-coenzyme A binding domains. Apart from the cloning from a brain cDNA library and expression profiling of Acbd5/KIAA1996 as part of a large-scale project (Ohara et al. 2002), both Acbd4 and Acbd5 appear to be uncharacterized. The significant CNS-related AS changes in exons from both Acbd4 and Acbd5 we have detected lead us to believe that these proteins could have a common function in the nervous system. Proline arginine rich coiled coil 1/RPRC1/BC019977 is an uncharacterized gene containing an alternative exon that we and Sugnet et al. (2006) have found to have neural-associated AS change. RPRC1 is part of a small family that contains an E-MAP-115 domain, which is present in the microtubule associated protein ensconsin/E-MAP-115/MAP7 (Faire et al. 1999, Bulinski et al. 2001).

Lrp12/St7 is a lipoprotein receptor that has been found to be misregulated in certain cancer cells (Qing et al. 1999, Battle et al. 2003, Garnis et al. 2004). We observed neural-regulated splicing of exon 7 of Lrp12, and the splicing of this exon is regulated by Nova-2 (Ule et al. 2005).

MATERIALS AND METHODS

Correlation of AS levels between exons in the same gene

Multiple distinct AS events from the same genes were grouped. The standard Spearman rank correlation coefficients were calculated for each pair of exon exclusion levels in the same gene. Also, the partial Spearman correlation coefficients of each exon's exclusion level with respect to the corresponding transcript levels (mean of the C1 and C2 probe levels) were calculated to remove possible effects of co-transcriptional coupling and/or technical error due to the changes in transcript level/measurement error. A lower threshold on acceptable expression levels was taken to be the 95th percentile of ~100 negative control probes on the arrays (the 95th percentile was found to be 5.25 on arcsinh scale), and AS events with transcript levels below this threshold were removed before analysis. Data for duplicate AS events were also removed before analysis. The exon pairs chosen for RT-PCR testing had absolute values of partial Spearman correlations greater than 0.6. The GenASAP values shown in Figure 2 and in Additional Data File 1 Figures 1 and 2 are based on a calibration method (Shai et al. 2006) using 241 RT-PCR measurements (not including RT-PCR measurements corresponding to the correlated exons in Additional Data File Figure 1), and there is a small but acceptable increase in measurement bias associated with these calibrated values.

A permutation-based resampling procedure was used to assess the statistical significance of the correlations. All exon labels and associated AS level profiles were randomly permuted so that exons from different genes were assigned to the same gene label. The correlations were computed as before, and this procedure was repeated 1000 times. The mean of the number of times a correlation coefficient greater than a given level (empirical cumulative distribution function) was observed in the permuted data was determined. These procedures were executed using the R statistical programming language/environment

(Ihaka and Gentleman, 1996; www.r-project.org).

Exon locations were mapped to the UCSC mouse genome assembly mm7 (Hinrichs et al. 2006) using NCBI-BLAST (Altschul et al. 1997). The Galaxy program (Giardine et al. 2005) was used to determine the number and lengths of exons in the interval between each exon pair using tracks containing exons from the UCSC Known mouse genes, Refseq genes, Ensembl genes, and MGC genes (Hinrichs et al. 2006). Where necessary, separate tracks were merged into a single track without any overlapping regions.

Detection of tissue group-specific AS events and transcript levels from microarray data

Tissues were classified into two main groups, one comprising central nervous system (brain and spinal cord) tissues and one group for all other tissues. Since the AS data often involves multiple outliers, we used robust linear models as implemented in the rlm function of the R package MASS (Venables and Ripley, 2002). It is not certain that the assumptions of normally distributed errors implicit in the use of standard linear models is correct for any given AS event. Thus, we used bootstrap resampling of model residuals using methods in the R package boot (Davison and Hinckley, 1997) to determine two-sided p-values that represent the fraction of bootstrap resamples in which the tissue grouping parameter's t-statistic was greater than observed for the model using the actual data. This procedure was implemented using a method adapted from Fox (2002). Additionally, we added to the model the corresponding event's transcript levels on each array (average of the C1 and C2 probe measurements) as a covariate to account for possible transcriptional coupling and/or technical error contributing to varying AS prediction qualities. The ~30 events referred to in the text as having significantly correlated AS-transcript level changes are events that show significant corrected p-values when using the model without transcript levels as a covariate, but that do not have significant corrected p-values when transcript levels are used as a model covariate. Each AS event's model residuals was resampled 100,000 times. This method is similar to that previously used to determine brain-regulated AS events from microarray data (Sugnet et al., 2006).

For an AS event to be included in our analyses, we required that expression (average of the probe set C1 and C2 probe measurements) was above the 95th percentile of background control probes in all 7 CNS tissues and in 8 other tissues. Due to the inherent measurement error of 10-15% for the GenASAP method (Pan et al., 2004; Pan et al., 2006), we required that the absolute value of the mean difference between the groups was at least 15% for the event to be considered a CNS-specific AS event. To account for the 3,707 comparisons made, a False Discovery Rate correction (with the original adjustment method) was used with a threshold of 0.05 (Benjamini and Hochberg, 1995). The muscle and embryonic tissue groups were analyzed using the procedures as above, with the groupings changed accordingly. Genes with CNS-specific transcript levels were determined using a resampling-based method and a FWER maxT correction using methods implemented in the R package multtest (Ge et al., 2003, Gentleman et al. 2004).

Further analyses of CNS-specific events

In most cases, the significance of proportion comparisons were calculated with Fisher's Exact test (R function fisher.test). In the case of the comparison of proportion of CNS-specific events with EST/cDNA evidence of isoform conservation, a logistic regression model (R function glm) was used with the tissue grouping effect, and CNS counts for both isoforms in mouse and human EST databases were included as covariates in order to correct for the higher counts of ESTs in the CNS-specific group. A t-test of the Tissue effect in the model was performed to assess the adjusted significance of the CNS grouping. EST counts used in the above analysis were from Pan et al. (2005).

The phastCons Most Conserved elements (Siepel et al., 2005) for mouse assembly mm7 (August 2005) were imported from the UCSC Genome Browser (Hinrichs et al., 2006) into the Galaxy program (Giardine et al., 2005). The number of nucleotides in the upstream and downstream intronic regions of the AS events on the array that overlap these Most Conserved elements was calculated using the Coverage command of the Galaxy program. Then, empirical cumulative distribution function plots of the number of nucleotides overlapping Most Conserved elements in each given region were created using the ecdf function in the stats package of the R language. The "other" events group definition was defined as those events that were tested using the linear model methods (ie. events that have expression above the 95th percentile of negative control probes in all 7 CNS tissues and >= 8 other tissues) and are not CNS-specific events.

Gene Ontology enrichment analysis

Gene Ontology (GO) enrichment analysis was performed using GOstat (Beissbarth and Speed, 2004). GOstat was used to determine p-values and these were calculated using a one-sided Fisher's exact test. These p-values were then False Discovery Rate-adjusted (Benjamini and Hochberg, 1995) to control for false positives due to the large number of GO terms tested. We assessed the over-representation of GO terms in the set of genes with CNS-specific AS events (including the events with significant AS-transcript level correlation), relative to the set of all other AS genes represented on the microarray that had expression above background in a sufficient number of tissues (i.e. all 7 CNS tissues and 8 other tissues).

Literature searching

The gene names and aliases of the tissue-specific events were retrieved from NCBI Entrez Gene database (Maglott et al. 2005), and the names of the orthologs of these genes were retrieved from the NCBI Homologene database (Wheeler et al. 2006). Using these gene names and aliases, Medline searches were performed using Pubmed (Wheeler et al., 2006). Literature searches were also performed using the text mining programs Chilibot (Chen and Sharp, 2004) and the Agilent Literature Search (Vailaya et al., 2005) plugin of the Cytoscape program (Shannon et al. 2003). Full-text articles were searched to find cases of tissue-specific functions and known alternative isoforms/exons. The Online Predicted Human Interaction Database (OPHID) (Brown and Jurisica, 2005) was searched for protein-protein interactions between genes with tissue-specific AS events. The Osprey program (Breitkreutz et al., 2003) was used to create a network diagram showing relationships between genes with CNS-specific AS events in signalling, cytoskeletal, and vesicle-mediated transport pathways, nervous system functions, and biological pathways.

Motif searches and analyses

<u>*Ab initio* motif search</u>

The *ab initio* motif search was performed using a modified version of the SeedSearcher algorithm (Barash et al., 2001). SeedSearcher identifies sequence motifs that discriminate a set of query sequences (the "positive" group) from a pool of control sequences (the "negative" group). In the present study, the following positive groups were defined: (a) CNSex - all alternative exons with higher exclusion ratios in CNS tissues; (b) CNSin - all alternative exons with higher inclusion ratios in CNS tissues; (c) CNSch – all alternative exons that present higher inclusion *or* exclusion ratios in CNS tissues. For each of these positive groups the non CNS-regulated AS events were used as the comparison or "negative" group. We searched for discriminative motifs in the following regions: the alternative exons ("A"), the constitutive exons directly upstream and downstream of each alternative exon (C1 and C2, respectively), the 150nt regions directly upstream and downstream of the alternative exons (AI1, AI2) and the 150nt intron

regions downstream of C1 (C1I1) and upstream of C2 (C2I2). To identify motifs which may operate in a flexible manner from one of two different regions, we also searched in the following concatenated regions: C1_C2, C1_A, A_C2, AI1_AI2, C1I1_AI1, AI2_C2I2 and C1I1_C2I2.

SeedSearcher was configured to search for motifs comprising short subsequences (or "seeds") of 5-20 nt with various degrees of sequence flexibility (i.e. "wild cards"). Based on initial tests we constrained the search runs to motifs of 5 nt (no wild cards), 6 nt (no wild cards), 7 nt (one wild card), 8 nt (2 wild cards) and 10 nt (with 2, 3 or 4 wild cards). SeedSearcher was configured to score each motif by assigning a hypergeometric p-value representing the probability of observing at least the same number of sequences from the "positive" group with the motif, given its abundance in the positive and negative groups (Barash et al., 2001). To correct for multiple hypothesis testing (each search setting included evaluating many possible motifs and therefore each motif can be considered as a "hypothesis") we used a random permutation test in which we computed the empirical p-value of scores in each setting, using 250 shuffled data sets such that the gene labels associated with the AS events and their corresponding exon and intron sequences were randomly associated with one another. We reported only motifs that had an empirical p-value < 0.05.

The statistical significance correction described above was aimed to handle multiple hypothesis testing resulting from many highly correlated motifs observed in a single search. However, our search also included motifs of different length and over different sequence definitions, some of which were concatenations. The p-value correction described above does not address this. To assess the statistical significance of the results under these search configurations, we also computed, using the 250x shuffled runs described above, the average and standard deviation for the number of motifs we observe as passing the corrected (empirical) p-value threshold, for each sequence/group search configuration and for the total number of all searches for each sequence or group. These results, along with the implied z-score of the number of motifs we observe are summarized in Additional Data File 8. Finally, We note that observing a motif as enriched in a concatenated region (e.g. AI1_AI2) does not necessarily mean it is not enriched in only a single region (e.g. AI1), nor does this imply that the change in its score over a single region is significant. For example, several of the motifs listed in Additional Data File 8 where found to have a similar score between single sequence areas and concatenations of two areas.

Comparisons of SeedSearcher-detected motifs with previously defined motifs:

Statistically significant motifs were compared against a database comprising previously reported, experimentally-defined, motifs associated with splicing (Fairbrother et al., 2002; Cartegni et al., 2003; Wang et al., 2004b; Zhang and Chasin, 2004; Stamm et al., 2006). These comparisons were performed using an algorithm that computes a similarity score for any two given PSSM (Position Specific Scoring Matrix), based on the best ungapped alignment of the two, allowing for partial overlap (Friedman et al., manuscript in preparation). The score for the alignment is based on the sum of the scores for all pairs of aligned positions. The score for aligning two positions (i.e. two distributions over A/C/G/U in this case) is based on the Jensen Shannon (JS) divergence (Lin et al., 1991) between the two distributions, penalized by the JS divergence between the average of the two distributions and the background distribution. Positions in non-overlapping regions are assumed to be aligned against a background distribution, which is uniform by default. For any motif aligned against a known motif of length K, we computed the empirical distribution of the scores we observe when aligning the motif against 10000 randomly chosen motifs of length K (the random motifs are generated by sampling from the set of all positions in the known motifs data base). If the score of the search motif aligned to a known motif has an

empirical p-value <0.005, we report the two as similar and present the alignment (refer to Additional Data File 5).

Searching with subsequences of known motifs:
We also searched the query sequences summarized above for enrichment of *cis* elements with a previously described link to regulation of AS in the nervous system (Additional Data File 6). The same scoring system (based on the hypergeometric p-value) was employed as described above. Since many of the reported motifs were either very short (i.e. 4 or 5 nt) or quite long (>12nt) we searched for all possible subsequences (i.e. with 1 nt shift) of length 7, 8, and 10 nt of the original motifs. Motifs less than 7 nt in length were self-concatenated to generate longer motifs for these searches. A total of ~350 subsequences were used in the searches. Results are only reported for enriched subsequences with a hypergeometric p value score <0.001 (Additional Data File 7).

Motif searching with Improbizer:
To further validate our motif search results and ensure the difference between our results and those reported by Sugnet et al. is not just a result of a difference in the motif search algorithm, we also performed motif search with the Improbizer software used by Sugnet et al. We used the same event groups and sequence regions as described above to perform *ab initio* motif searches with the Improbizer algorithm described by Sugnet et al (2006). The searches were performed as per instructions provided by the Improbizer browser (http://www.soe.ucsc.edu/~kent/improbizer/improbizer.html). Default parameters were used, with the number of reported motifs increased to 5 to avoid missing any significant motifs (in practice only the first one or two motifs reported in each search were found to be significant as described below). We ran each Improbizer search an additional 250 times using the randomization option of the software. Motifs were scored as significant if they exceeded the 95 percentile of the scores of the matching 250 random runs (i.e. their score had an empirical p-value less then 0.05 according to these score definitions).

Motif conservation analysis:
A SeedSearcher motif detected in a specified mouse sequence was considered to be "conserved" if the same motif could also be detected within the corresponding intron or exon sequence of the orthologous human gene (i.e. the actual motif location need not be the same). Thus, this definition of conservation does not rely on a specific global alignment and is not effected by insertion/deletion events that shift the motif. Accordingly, the conservation ratio (CR) of a SeedSearcher motif in a given set of sequences was defined as the ratio between the number of mouse sequences it was originally found in and the number of orthologous human sequences it was found to be conserved in. To analyze the relative conservation of the motifs identified by SeedSearcher, we performed the following procedure: First, the mouse exon sequences were mapped to the UCSC human genome hg18 based on pre-computed blastZ alignments using the Galaxy program (Giardine et al. 2005). The 150 nucleotide flanking intron sequences were then extracted from the same human genome build based on the exon coordinates. Of the original 3707 cassette events, 3164 to 3410 events could be unambiguously mapped to the corresponding human genomic sequences, depending on the sequence region. The size of the "positive" CNS groups originally defined for the SeedSearcher analysis varied from 35 (CNSex, "C1_A" exon region) to 100 (CNSch, C2I2 region). We then estimated the average CR (P) of motifs of a specific configuration (i.e. same length and number of wild card positions) in a specific sequence region and for a given set of events (e.g. CNSin) by randomly sampling 1000 subsequences of the specified configuration from the same group of sequences. We then compared the number of times (X) a given motif identified by SeedSearcher was found to be conserved out of the total number of times (N) it occurred in the given set of sequences, and computed the statistical significance of this conservation ratio using the tail of a Binomial distribution

(Binomial p-value(X|N,P)). This statistical test corresponds to the following hypothesis testing: Assuming the conservation of the given motif's occurrences has the same ratio as the average one (P), what is the probability of observing its conservation in at least X out of N occurrences? If this latter probability is low (p-value < 0.05) we reject this hypothesis and declare the motif to be significantly conserved. The results are summarized in Additional Data File 10. For 25/26 of the motifs analyzed, their CR was found to be higher in the "positive" groups (a positive number in column "RatioDiff" in the table), i.e. the motifs were found to be more conserved in the groups identified with CNS regulated splicing. The average CR for randomly selected motifs was also found to be higher in the "positive" groups (a positive number in column "RandRatioDiff" in the table). This result is consistent with the observation of enrichment of PhastCons regions in the intron sequences flanking the CNS regulated AS events (see main text; Sugnet et. al, 2005). Most importantly, 20/26 of the motifs had a CR that is higher than the average "background" conservation level in the same regions, and in 17 of these cases this difference was found to be statistically significant (last column in Additional Data File 10)

REFERENCES

Abell, A. N., Rivera-Perez, J. A., Cuevas, B. D., Uhlik, M. T., Sather, S., Johnson, N. L., Minton, S. K., Lauder, J. M., Winter-Vann, A. M., Nakamura, K., *et al.* (2005). Ablation of MEKK4 kinase activity causes neurulation and skeletal patterning defects in the mouse embryo. Mol Cell Biol *25*, 8948-8959.

Acarin, L., Paris, J., Gonzalez, B., and Castellano, B. (2002). Glial expression of small heat shock proteins following an excitotoxic lesion in the immature rat brain. Glia *38*, 1-14.

Alexander, C., Votruba, M., Pesch, U. E., Thiselton, D. L., Mayer, S., Moore, A., Rodriguez, M., Kellner, U., Leo-Kottler, B., Auburger, G., *et al.* (2000). OPA1, encoding a dynamin-related GTPase, is mutated in autosomal dominant optic atrophy linked to chromosome 3q28. Nat Genet *26*, 211-215.

Altschul S.F., Madden T.L., Schaffer A.A., Zhang J., Zhang Z., Miller W., Lipman D.J. (1997). Gapped BLAST and PSI-BLAST: a new generation of protein database search programs. Nucleic Acids Res. *17*, 3389-3402.

Aoki, K., Nakamura, T., Fujikawa, K., and Matsuda, M. (2005). Local phosphatidylinositol 3,4,5-trisphosphate accumulation recruits Vav2 and Vav3 to activate Rac1/Cdc42 and initiate neurite outgrowth in nerve growth factor-stimulated PC12 cells. Mol Biol Cell *16*, 2207-2217.

Apel, E. D., Lewis, R. M., Grady, R. M., and Sanes, J. R. (2000). Syne-1, a dystrophin- and Klarsicht-related protein associated with synaptic nuclei at the neuromuscular junction. J Biol Chem *275*, 31986-31995.

Arevalo, J. C., Pereira, D. B., Yano, H., Teng, K. K., and Chao, M. V. (2006). Identification of a switch in neurotrophin signaling by selective tyrosine phosphorylation. J Biol Chem *281*, 1001-1007.

Arevalo, J. C., Yano, H., Teng, K. K., and Chao, M. V. (2004). A unique pathway for sustained neurotrophin signaling through an ankyrin-rich membrane-spanning protein. Embo J *23*, 2358-2368.

Ashiya, M., and Grabowski, P. J. (1997). A neuron-specific splicing switch mediated by an array of pre-mRNA repressor sites: evidence of a regulatory role for the polypyrimidine tract binding protein and a brain-specific PTB counterpart. Rna *3*, 996-1015.

Ashour, F., and Deuchars, J. (2004). Electron microscopic localisation of P2X4 receptor subunit immunoreactivity to pre- and post-synaptic neuronal elements and glial processes in the dorsal vagal complex of the rat. Brain Res *1026*, 44-55.

Audebert, S., Navarro, C., Nourry, C., Chasserot-Golaz, S., Lecine, P., Bellaiche, Y., Dupont, J. L., Premont, R. T., Sempere, C., Strub, J. M., *et al.* (2004). Mammalian Scribble forms a tight complex with the betaPIX exchange factor. Curr Biol *14*, 987-995.

Azim, A. C., Kim, A. C., Lutchman, M., Andrabi, S., Peters, L. L., and Chishti, A. H. (1999). cDNA sequence, genomic structure, and expression of the mouse dematin gene (Epb4.9). Mamm Genome *10*, 1026-1029.

Azim, A. C., Knoll, J. H., Beggs, A. H., and Chishti, A. H. (1995). Isoform cloning, actin binding, and chromosomal localization of human erythroid dematin, a member of the villin superfamily. J Biol Chem *270*, 17407-17413.

Azim, A. C., Marfatia, S. M., Korsgren, C., Dotimas, E., Cohen, C. M., and Chishti, A. H. (1996). Human erythrocyte dematin and protein 4.2 (pallidin) are ATP binding proteins. Biochemistry *35*, 3001-3006.

Bach, D., Pich, S., Soriano, F. X., Vega, N., Baumgartner, B., Oriola, J., Daugaard, J. R., Lloberas, J., Camps, M., Zierath, J. R., *et al.* (2003). Mitofusin-2 determines mitochondrial network architecture and mitochondrial metabolism. A novel regulatory mechanism altered in obesity. J Biol Chem *278*, 17190-17197.

Ballif, B. A., Arnaud, L., Arthur, W. T., Guris, D., Imamoto, A., and Cooper, J. A. (2004). Activation of a Dab1/CrkL/C3G/Rap1 pathway in Reelin-stimulated neurons. Curr Biol *14*, 606-610.

Barash, Y., Bejerano, G., and Friedman, N. (2001). A simple hyper-geometric approach for discovering putative transcription

factor binding sites. . Lecture Notes in Computer Science *2149*, 278-293.
Bayer, K. U., De Koninck, P., and Schulman, H. (2002). Alternative splicing modulates the frequency-dependent response of CaMKII to Ca(2+) oscillations. Embo J *21*, 3590-3597.
Becamel, C., Alonso, G., Galeotti, N., Demey, E., Jouin, P., Ullmer, C., Dumuis, A., Bockaert, J., and Marin, P. (2002). Synaptic multiprotein complexes associated with 5-HT(2C) receptors: a proteomic approach. Embo J *21*, 2332-2342.
Beissbarth, T. and Speed, T.P. (2004). GOstat: find statistically overrepresented Gene Ontologies within a group of genes. Bioinformatics. *12*, 1464-1465.
Belfort, G. M., and Kandror, K. V. (2003). Cellugyrin and synaptogyrin facilitate targeting of synaptophysin to a ubiquitous synaptic vesicle-sized compartment in PC12 cells. J Biol Chem *278*, 47971-47978.
Bence, M., Arbuckle, M. I., Dickson, K. S., and Grant, S. G. (2005). Analyses of murine postsynaptic density-95 identify novel isoforms and potential translational control elements. Brain Res Mol Brain Res *133*, 143-152.
Benjamini, Y. and Hochberg, Y. (1995). Controlling the False Discovery Rate- a practical and powerful approach to multiple testing. Journal of the Royal Statistical Society Series B- Methodological *57*, 289-300.
Benjamini, Y. and Yekutieli, D. (2001). The control of the false discovery rate in multiple testing under dependency. Annals of Statistics *29*, 1165-1188.
Bobanovic, L. K., Royle, S. J., and Murrell-Lagnado, R. D. (2002). P2X receptor trafficking in neurons is subunit specific. J Neurosci *22*, 4814-4824.
Breitkreutz, B. J., Stark, C., and Tyers, M. (2003). Osprey: a network visualization system. Genome Biol *4*, R22.
Bridgman, P. C. (1999). Myosin Va movements in normal and dilute-lethal axons provide support for a dual filament motor complex. J Cell Biol *146*, 1045-1060.
Briggs, M. W., and Sacks, D. B. (2003). IQGAP proteins are integral components of cytoskeletal regulation. EMBO Rep *4*, 571-574.
Brondyk, W. H., McKiernan, C. J., Fortner, K. A., Stabila, P., Holz, R. W., and Macara, I. G. (1995). Interaction cloning of Rabin3, a novel protein that associates with the Ras-like GTPase Rab3A. Mol Cell Biol *15*, 1137-1143.
Brown, A., Bernier, G., Mathieu, M., Rossant, J., and Kothary, R. (1995). The mouse dystonia musculorum gene is a neural isoform of bullous pemphigoid antigen 1. Nat Genet *10*, 301-306.
Brown, K. R., and Jurisica, I. (2005). Online predicted human interaction database. Bioinformatics *21*, 2076-2082.
Burd, C. G., and Dreyfuss, G. (1994). RNA binding specificity of hnRNP A1: significance of hnRNP A1 high-affinity binding sites in pre-mRNA splicing. Embo J *13*, 1197-1204.
Butler, M. H., David, C., Ochoa, G. C., Freyberg, Z., Daniell, L., Grabs, D., Cremona, O., and De Camilli, P. (1997). Amphiphysin II (SH3P9; BIN1), a member of the amphiphysin/Rvs family, is concentrated in the cortical cytomatrix of axon initial segments and nodes of ranvier in brain and around T tubules in skeletal muscle. J Cell Biol *137*, 1355-1367.
Cabrera-Poch, N., Sanchez-Ruiloba, L., Rodriguez-Martinez, M., and Iglesias, T. (2004). Lipid raft disruption triggers protein kinase C and Src-dependent protein kinase D activation and Kidins220 phosphorylation in neuronal cells. J Biol Chem *279*, 28592-28602.
Cao, H., Garcia, F., and McNiven, M. A. (1998). Differential distribution of dynamin isoforms in mammalian cells. Mol Biol Cell *9*, 2595-2609.
Cao, X., and Sudhof, T. C. (2001). A transcriptionally [correction of transcriptively] active complex of APP with Fe65 and histone acetyltransferase Tip60. Science *293*, 115-120.
Carpenter, D., Meadows, H. J., Brough, S., Chapman, G., Clarke, C., Coldwell, M., Davis, R., Harrison, D., Meakin, J., McHale, M.*, et al.* (1999). Site-specific splice variation of the human P2X4 receptor. Neurosci Lett *273*, 183-186.
Cartegni, L., Wang, J., Zhu, Z., Zhang, M. Q., and Krainer, A. R. (2003). ESEfinder: A web resource to identify exonic splicing enhancers. Nucleic Acids Res *31*, 3568-3571.
Cartoni, R., Leger, B., Hock, M. B., Praz, M., Crettenand, A., Pich, S., Ziltener, J. L., Luthi, F., Deriaz, O., Zorzano, A.*, et al.* (2005). Mitofusins 1/2 and ERRalpha expression are increased in human skeletal muscle after physical exercise. J Physiol *567*, 349-358.
Caudy, M., Vassin, H., Brand, M., Tuma, R., Jan, L. Y., and Jan, Y. N. (1988). daughterless, a Drosophila gene essential for both neurogenesis and sex determination, has sequence similarities to myc and the achaete-scute complex. Cell *55*, 1061-1067.
Cecconi, F., Proetzel, G., Alvarez-Bolado, G., Jay, D., and Gruss, P. (1997). Expression of Meis2, a Knotted-related murine homeobox gene, indicates a role in the differentiation of the forebrain and the somitic mesoderm. Dev Dyn *210*, 184-190.
Chan, R. C., and Black, D. L. (1997). The polypyrimidine tract binding protein binds upstream of neural cell-specific c-src exon N1 to repress the splicing of the intron downstream. Mol Cell Biol *17*, 4667-4676.
Chang, M. S., Arevalo, J. C., and Chao, M. V. (2004). Ternary complex with Trk, p75, and an ankyrin-rich membrane spanning protein. J Neurosci Res *78*, 186-192.
Chen, H., Detmer, S. A., Ewald, A. J., Griffin, E. E., Fraser, S. E., and Chan, D. C. (2003). Mitofusins Mfn1 and Mfn2 coordinately regulate mitochondrial fusion and are essential for embryonic development. J Cell Biol *160*, 189-200.
Chi, H., Sarkisian, M. R., Rakic, P., and Flavell, R. A. (2005). Loss of mitogen-activated protein kinase kinase kinase 4 (MEKK4) results in enhanced apoptosis and defective neural tube development. Proc Natl Acad Sci U S A *102*, 3846-3851.

Chen, H., and Sharp, B. M. (2004). Content-rich biological network constructed by mining PubMed abstracts. BMC Bioinformatics *5*, 147.
Chiaramello, A., Soosaar, A., Neuman, T., and Zuber, M. X. (1995). Differential expression and distinct DNA-binding specificity of ME1a and ME2 suggest a unique role during differentiation and neuronal plasticity. Brain Res Mol Brain Res *29*, 107-118.
Chou, M. Y., Underwood, J. G., Nikolic, J., Luu, M. H., and Black, D. L. (2000). Multisite RNA binding and release of polypyrimidine tract binding protein during the regulation of c-src neural-specific splicing. Mol Cell *5*, 949-957.
Cipolat, S., Martins de Brito, O., Dal Zilio, B., and Scorrano, L. (2004). OPA1 requires mitofusin 1 to promote mitochondrial fusion. Proc Natl Acad Sci U S A *101*, 15927-15932.
Clark, M. B., Ma, Y., Bloom, M. L., Barker, J. E., Zagon, I. S., Zimmer, W. E., and Goodman, S. R. (1994). Brain alpha erythroid spectrin: identification, compartmentalization, and beta spectrin associations. Brain Res *663*, 223-236.
Colbran, R. J., and Brown, A. M. (2004). Calcium/calmodulin-dependent protein kinase II and synaptic plasticity. Curr Opin Neurobiol *14*, 318-327.
Comtesse, N., Niedermayer, I., Glass, B., Heckel, D., Maldener, E., Nastainczyk, W., Feiden, W., and Meese, E. (2002). MGEA6 is tumor-specific overexpressed and frequently recognized by patient-serum antibodies. Oncogene *21*, 239-247.
Cote, J. F., and Vuori, K. (2002). Identification of an evolutionarily conserved superfamily of DOCK180-related proteins with guanine nucleotide exchange activity. J Cell Sci *115*, 4901-4913.
Cottrell, J. R., Borok, E., Horvath, T. L., and Nedivi, E. (2004). CPG2: a brain- and synapse-specific protein that regulates the endocytosis of glutamate receptors. Neuron *44*, 677-690.
Cowan, C. W., Shao, Y. R., Sahin, M., Shamah, S. M., Lin, M. Z., Greer, P. L., Gao, S., Griffith, E. C., Brugge, J. S., and Greenberg, M. E. (2005). Vav family GEFs link activated Ephs to endocytosis and axon guidance. Neuron *46*, 205-217.
Cox, P. R., Fowler, V., Xu, B., Sweatt, J. D., Paylor, R., and Zoghbi, H. Y. (2003). Mice lacking Tropomodulin-2 show enhanced long-term potentiation, hyperactivity, and deficits in learning and memory. Mol Cell Neurosci *23*, 1-12.
Cox, P. R., and Zoghbi, H. Y. (2000). Sequencing, expression analysis, and mapping of three unique human tropomodulin genes and their mouse orthologs. Genomics *63*, 97-107.
Cuadra, A. E., Kuo, S. H., Kawasaki, Y., Bredt, D. S., and Chetkovich, D. M. (2004). AMPA receptor synaptic targeting regulated by stargazin interactions with the Golgi-resident PDZ protein nPIST. J Neurosci *24*, 7491-7502.
Dan, I., Watanabe, N. M., Kobayashi, T., Yamashita-Suzuki, K., Fukagaya, Y., Kajikawa, E., Kimura, W. K., Nakashima, T. M., Matsumoto, K., Ninomiya-Tsuji, J., and Kusumi, A. (2000). Molecular cloning of MINK, a novel member of mammalian GCK family kinases, which is up-regulated during postnatal mouse cerebral development. FEBS Lett *469*, 19-23.
Davison, A. C. and Hinkley, D. V. (1997). Bootstrap Methods and Their Application . Cambridge University Press.
Deguchi-Tawarada, M., Inoue, E., Takao-Rikitsu, E., Inoue, M., Ohtsuka, T., and Takai, Y. (2004). CAST2: identification and characterization of a protein structurally related to the presynaptic cytomatrix protein CAST. Genes Cells *9*, 15-23.
Delettre, C., Lenaers, G., Griffoin, J. M., Gigarel, N., Lorenzo, C., Belenguer, P., Pelloquin, L., Grosgeorge, J., Turc-Carel, C., Perret, E., *et al.* (2000). Nuclear gene OPA1, encoding a mitochondrial dynamin-related protein, is mutated in dominant optic atrophy. Nat Genet *26*, 207-210.
Dobrosotskaya, I., Guy, R. K., and James, G. L. (1997). MAGI-1, a membrane-associated guanylate kinase with a unique arrangement of protein-protein interaction domains. J Biol Chem *272*, 31589-31597.
Donai, H., Murakami, T., Amano, T., Sogawa, Y., and Yamauchi, T. (2000). Induction and alternative splicing of delta isoform of Ca(2+)/calmodulin-dependent protein kinase II during neural differentiation of P19 embryonal carcinoma cells and during brain development. Brain Res Mol Brain Res *85*, 189-199.
Dong, H., Light, P. E., French, R. J., and Lytton, J. (2001). Electrophysiological characterization and ionic stoichiometry of the rat brain K(+)-dependent NA(+)/CA(2+) exchanger, NCKX2. J Biol Chem *276*, 25919-25928.
Drosopoulos, N. E., Walsh, F. S., and Doherty, P. (1999). A soluble version of the receptor-like protein tyrosine phosphatase kappa stimulates neurite outgrowth via a Grb2/MEK1-dependent signaling cascade. Mol Cell Neurosci *13*, 441-449.
Dunah, A. W., Hueske, E., Wyszynski, M., Hoogenraad, C. C., Jaworski, J., Pak, D. T., Simonetta, A., Liu, G., and Sheng, M. (2005). LAR receptor protein tyrosine phosphatases in the development and maintenance of excitatory synapses. Nat Neurosci *8*, 458-467.
Erkman, L., Yates, P. A., McLaughlin, T., McEvilly, R. J., Whisenhunt, T., O'Connell, S. M., Krones, A. I., Kirby, M. A., Rapaport, D. H., Bermingham, J. R., *et al.* (2000). A POU domain transcription factor-dependent program regulates axon pathfinding in the vertebrate visual system. Neuron *28*, 779-792.
Fairbrother, W. G., Yeh, R. F., Sharp, P. A., and Burge, C. B. (2002). Predictive identification of exonic splicing enhancers in human genes. Science *297*, 1007-1013.
Farhan, H., Korkhov, V. M., Paulitschke, V., Dorostkar, M. M., Scholze, P., Kudlacek, O., Freissmuth, M., and Sitte, H. H. (2004). Two discontinuous segments in the carboxyl terminus are required for membrane targeting of the rat gamma-aminobutyric acid transporter-1 (GAT1). J Biol Chem *279*, 28553-28563.
Fournier, K. M., and Robinson, M. B. (2006). A dominant-negative variant of SNAP-23 decreases the cell surface expression of the neuronal glutamate transporter EAAC1 by slowing constitutive delivery. Neurochem Int *48*, 596-603.

Fox, J. (2002). An R and S-Plus Companion to Applied Regression. Sage Publications, Thousand Oaks, CA, USA.
Frank, B. S., Vardar, D., Chishti, A. H., and McKnight, C. J. (2004). The NMR structure of dematin headpiece reveals a dynamic loop that is conformationally altered upon phosphorylation at a distal site. J Biol Chem *279*, 7909-7916.
Fukata, M., Watanabe, T., Noritake, J., Nakagawa, M., Yamaga, M., Kuroda, S., Matsuura, Y., Iwamatsu, A., Perez, F., and Kaibuchi, K. (2002). Rac1 and Cdc42 capture microtubules through IQGAP1 and CLIP-170. Cell *109*, 873-885.
Gad, J. M., Keeling, S. L., Wilks, A. F., Tan, S. S., and Cooper, H. M. (1997). The expression patterns of guidance receptors, DCC and Neogenin, are spatially and temporally distinct throughout mouse embryogenesis. Dev Biol *192*, 258-273.
Galus, A., Lagos, A., Romanik, E. A., and O'Connor, C. M. (1994). Structural analysis of transcripts for the protein L-isoaspartyl methyltransferase reveals multiple transcription initiation sites and a distinct pattern of expression in mouse testis: identification of a 5'-flanking sequence with promoter activity. Arch Biochem Biophys *312*, 524-533.
Gao, X., Satoh, T., Liao, Y., Song, C., Hu, C. D., Kariya Ki, K., and Kataoka, T. (2001). Identification and characterization of RA-GEF-2, a Rap guanine nucleotide exchange factor that serves as a downstream target of M-Ras. J Biol Chem *276*, 42219-42225.
Ge, Y. C.; Dudoit, S. & Speed, T. P. Resampling-based multiple testing for microarray data analysis. (2003). Test, *12*, 1-77
Ge, K., DuHadaway, J., Du, W., Herlyn, M., Rodeck, U., and Prendergast, G. C. (1999). Mechanism for elimination of a tumor suppressor: aberrant splicing of a brain-specific exon causes loss of function of Bin1 in melanoma. Proc Natl Acad Sci U S A *96*, 9689-9694.
Gelot, A., Moreau, J., Khrestchatisky, M., Ben Ari, Y., and Pollard, H. (1994). Developmental change of alpha-spectrin mRNA in the rat brain. Brain Res Dev Brain Res *81*, 240-246.
Gentleman, R. C.; Carey, V. J.; Bates, D. M.; Bolstad, B.; Dettling, M.; Dudoit, S.; Ellis, B.; Gautier, L.; Ge, Y.; Gentry, J.; Hornik, K.; Hothorn, T.; Huber, W.; Iacus, S.; Irizarry, R.; Leisch, F.; Li, C.; Maechler, M.;
Rossini, A. J.; Sawitzki, G.; Smith, C.; Smyth, G.; Tierney, L.; Yang, J. Y. H. & Zhang, J. Bioconductor: open software development for computational biology and bioinformatics. (2004). Genome Biol, *5*, R80
Gerges, N. Z., Backos, D. S., Rupasinghe, C. N., Spaller, M. R., and Esteban, J. A. (2006). Dual role of the exocyst in AMPA receptor targeting and insertion into the postsynaptic membrane. Embo J *25*, 1623-1634.
Gerwins, P., Blank, J. L., and Johnson, G. L. (1997). Cloning of a novel mitogen-activated protein kinase kinase kinase, MEKK4, that selectively regulates the c-Jun amino terminal kinase pathway. J Biol Chem *272*, 8288-8295.
Giardine B., Riemer C., Hardison R.C., Burhans R., Elnitski L., Shah P., Zhang Y., Blankenberg D., Albert I., Taylor J., Miller W., Kent W.J., Nekrutenko A. (2005) Galaxy: a platform for interactive large-scale genome analysis. Genome Res. *10*,1451-1455.
Grady, R. M., Starr, D. A., Ackerman, G. L., Sanes, J. R., and Han, M. (2005). Syne proteins anchor muscle nuclei at the neuromuscular junction. Proc Natl Acad Sci U S A *102*, 4359-4364.
Graef, I. A., Wang, F., Charron, F., Chen, L., Neilson, J., Tessier-Lavigne, M., and Crabtree, G. R. (2003). Neurotrophins and netrins require calcineurin/NFAT signaling to stimulate outgrowth of embryonic axons. Cell *113*, 657-670.
Greller, L.D. and Tobin, F.L. (1999). Detecting selective expression of genes and proteins. Genome Res. *3*, 282-96.
Griparic, L., van der Wel, N. N., Orozco, I. J., Peters, P. J., and van der Bliek, A. M. (2004). Loss of the intermembrane space protein Mgm1/OPA1 induces swelling and localized constrictions along the lengths of mitochondria. J Biol Chem *279*, 18792-18798.
Grubbs, F.E. (1950). Sample criteria for testing outlying observations. Annals of Mathematical Statistics. *21*, 27-58.
Grubbs, F.E. (1969). Procedures for detecting outlying observations in samples. Technometrics. *11*. 1.
Guo, L., Degenstein, L., Dowling, J., Yu, Q. C., Wollmann, R., Perman, B., and Fuchs, E. (1995). Gene targeting of BPAG1: abnormalities in mechanical strength and cell migration in stratified epithelia and neurologic degeneration. Cell *81*, 233-243.
Hagens, O., Minina, E., Schweiger, S., Ropers, H. H., and Kalscheuer, V. (2006). Characterization of FBX25, encoding a novel brain-expressed F-box protein. Biochim Biophys Acta *1760*, 110-118.
Hashimoto, K., Miyata, M., Watanabe, M., and Kano, M. (2001). Roles of phospholipase Cbeta4 in synapse elimination and plasticity in developing and mature cerebellum. Mol Neurobiol *23*, 69-82.
Hassan, B., and Vaessin, H. (1997). Daughterless is required for the expression of cell cycle genes in peripheral nervous system precursors of Drosophila embryos. Dev Genet *21*, 117-122.
Hassel, B., Schreff, M., Stube, E. M., Blaich, U., and Schumacher, S. (2003). CALEB/NGC interacts with the Golgi-associated protein PIST. J Biol Chem *278*, 40136-40143.
Hata, Y., and Takai, Y. (1999). Roles of postsynaptic density-95/synapse-associated protein 90 and its interacting proteins in the organization of synapses. Cell Mol Life Sci *56*, 461-472.
Hattula, K., Furuhjelm, J., Arffman, A., and Peranen, J. (2002). A Rab8-specific GDP/GTP exchange factor is involved in actin remodeling and polarized membrane transport. Mol Biol Cell *13*, 3268-3280.
Hawley, S. A., Pan, D. A., Mustard, K. J., Ross, L., Bain, J., Edelman, A. M., Frenguelli, B. G., and Hardie, D. G. (2005). Calmodulin-dependent protein kinase kinase-beta is an alternative upstream kinase for AMP-activated protein kinase. Cell Metab *2*, 9-19.
He, J., Bellini, M., Inuzuka, H., Xu, J., Xiong, Y., Yang, X., Castleberry, A. M., and Hall, R. A. (2006). Proteomic analysis of

beta1-adrenergic receptor interactions with PDZ scaffold proteins. J Biol Chem *281*, 2820-2827.
He, L., Lu, X. Y., Jolly, A. F., Eldridge, A. G., Watson, S. J., Jackson, P. K., Barsh, G. S., and Gunn, T. M. (2003). Spongiform degeneration in mahoganoid mutant mice. Science *299*, 710-712.
Heckel, D., Brass, N., Fischer, U., Blin, N., Steudel, I., Tureci, O., Fackler, O., Zang, K. D., and Meese, E. (1997). cDNA cloning and chromosomal mapping of a predicted coiled-coil proline-rich protein immunogenic in meningioma patients. Hum Mol Genet *6*, 2031-2041.
Higuchi, M., Maas, S., Single, F. N., Hartner, J., Rozov, A., Burnashev, N., Feldmeyer, D., Sprengel, R., and Seeburg, P. H. (2000). Point mutation in an AMPA receptor gene rescues lethality in mice deficient in the RNA-editing enzyme ADAR2. Nature *406*, 78-81.
Hinrichs, A.S., Karolchik, D., Baertsch, R., Barber G.P., Bejerano G., Clawson H., Diekhans M., Furey T.S., Harte R.A., Hsu F., Hillman-Jackson J., Kuhn R.M., Pedersen J.S., Pohl A., Raney B.J., Rosenbloom K.R., Siepel A., Smith K.E., Sugnet C.W., Sultan-Qurraie A., Thomas D.J., Trumbower H., Weber R.J., Weirauch M., Zweig A.S., Haussler D., Kent W.J. (2006). The UCSC Genome Browser Database: update 2006. Nucleic Acids Res. D590-598.
Hirokawa, N., and Takemura, R. (2005). Molecular motors and mechanisms of directional transport in neurons. Nat Rev Neurosci *6*, 201-214.
Hirono, M., Sugiyama, T., Kishimoto, Y., Sakai, I., Miyazawa, T., Kishio, M., Inoue, H., Nakao, K., Ikeda, M., Kawahara, S., *et al.* (2001). Phospholipase Cbeta4 and protein kinase Calpha and/or protein kinase CbetaI are involved in the induction of long term depression in cerebellar Purkinje cells. J Biol Chem *276*, 45236-45242.
Ho, Y. D., Joyal, J. L., Li, Z., and Sacks, D. B. (1999). IQGAP1 integrates Ca2+/calmodulin and Cdc42 signaling. J Biol Chem *274*, 464-470.
Hofmann, Y., Lorson, C. L., Stamm, S., Androphy, E. J., and Wirth, B. (2000). Htra2-beta 1 stimulates an exonic splicing enhancer and can restore full-length SMN expression to survival motor neuron 2 (SMN2). Proc Natl Acad Sci U S A *97*, 9618-9623.
Honkaniemi, J., Zhang, J. S., Yang, T., Zhang, C., Tisi, M. A., and Longo, F. M. (1998). LAR tyrosine phosphatase receptor: proximal membrane alternative splicing is coordinated with regional expression and intraneuronal localization. Brain Res Mol Brain Res *60*, 1-12.
Hruska-Hageman, A. M., Benson, C. J., Leonard, A. S., Price, M. P., and Welsh, M. J. (2004). PSD-95 and Lin-7b interact with acid-sensing ion channel-3 and have opposite effects on H+- gated current. J Biol Chem *279*, 46962-46968.
Hu, Q., Hearn, M. G., Jin, L. W., Bressler, S. L., and Martin, G. M. (1999). Alternatively spliced isoforms of FE65 serve as neuron-specific and non-neuronal markers. J Neurosci Res *58*, 632-640.
Hu, W. H., Pendergast, J. S., Mo, X. M., Brambilla, R., Bracchi-Ricard, V., Li, F., Walters, W. M., Blits, B., He, L., Schaal, S. M., and Bethea, J. R. (2005). NIBP, a novel NIK and IKK(beta)-binding protein that enhances NF-(kappa)B activation. J Biol Chem *280*, 29233-29241.
Hu, Y., Leo, C., Yu, S., Huang, B. C., Wang, H., Shen, M., Luo, Y., Daniel-Issakani, S., Payan, D. G., and Xu, X. (2004). Identification and functional characterization of a novel human misshapen/Nck interacting kinase-related kinase, hMINK beta. J Biol Chem *279*, 54387-54397.
Huang, Y. S., Jung, M. Y., Sarkissian, M., and Richter, J. D. (2002). N-methyl-D-aspartate receptor signaling results in Aurora kinase-catalyzed CPEB phosphorylation and alpha CaMKII mRNA polyadenylation at synapses. Embo J *21*, 2139-2148.
Huynh, D. P., Figueroa, K., Hoang, N., and Pulst, S. M. (2000). Nuclear localization or inclusion body formation of ataxin-2 are not necessary for SCA2 pathogenesis in mouse or human. Nat Genet *26*, 44-50.
Iglesias, T., Cabrera-Poch, N., Mitchell, M. P., Naven, T. J., Rozengurt, E., and Schiavo, G. (2000). Identification and cloning of Kidins220, a novel neuronal substrate of protein kinase D. J Biol Chem *275*, 40048-40056.
Ihaka R. and Gentleman R. (1996), "R: A Language for Data Analysis and Graphics", Journal of Computational and Graphical Statistics. *5*, 299–314
Iijima, T., Imai, T., Kimura, Y., Bernstein, A., Okano, H. J., Yuzaki, M., and Okano, H. (2005). Hzf protein regulates dendritic localization and BDNF-induced translation of type 1 inositol 1,4,5-trisphosphate receptor mRNA. Proc Natl Acad Sci U S A *102*, 17190-17195.
Ikegaya, Y., Yamada, M., Fukuda, T., Kuroyanagi, H., Shirasawa, T., and Nishiyama, N. (2001). Aberrant synaptic transmission in the hippocampal CA3 region and cognitive deterioration in protein-repair enzyme-deficient mice. Hippocampus *11*, 287-298.
Imamura, R., Masuda, E. S., Naito, Y., Imai, S., Fujino, T., Takano, T., Arai, K., and Arai, N. (1998). Carboxyl-terminal 15-amino acid sequence of NFATx1 is possibly created by tissue-specific splicing and is essential for transactivation activity in T cells. J Immunol *161*, 3455-3463.
Imbert, G., Saudou, F., Yvert, G., Devys, D., Trottier, Y., Garnier, J. M., Weber, C., Mandel, J. L., Cancel, G., Abbas, N., *et al.* (1996). Cloning of the gene for spinocerebellar ataxia 2 reveals a locus with high sensitivity to expanded CAG/glutamine repeats. Nat Genet *14*, 285-291.
Janz, R., Sudhof, T. C., Hammer, R. E., Unni, V., Siegelbaum, S. A., and Bolshakov, V. Y. (1999). Essential roles in synaptic plasticity for synaptogyrin I and synaptophysin I. Neuron *24*, 687-700.

Jayanthi, S., Deng, X., Ladenheim, B., McCoy, M. T., Cluster, A., Cai, N. S., and Cadet, J. L. (2005). Calcineurin/NFAT-induced up-regulation of the Fas ligand/Fas death pathway is involved in methamphetamine-induced neuronal apoptosis. Proc Natl Acad Sci U S A *102*, 868-873.
Jensen, K. B., Musunuru, K., Lewis, H. A., Burley, S. K., and Darnell, R. B. (2000). The tetranucleotide UCAY directs the specific recognition of RNA by the Nova K-homology 3 domain. Proc Natl Acad Sci U S A *97*, 5740-5745.
Lin, J. Divergence measures based on the Shannon entropy. (1991). IEEE Trans. Inform. Theory, *37*, 145– 151.
Jin, Y., Suzuki, H., Maegawa, S., Endo, H., Sugano, S., Hashimoto, K., Yasuda, K., and Inoue, K. (2003). A vertebrate RNA-binding protein Fox-1 regulates tissue-specific splicing via the pentanucleotide GCAUG. Embo J *22*, 905-912.
Jones, E. A., Sun, D., Kobierski, L., and Symes, A. J. (2003). NFAT4 is expressed in primary astrocytes and activated by glutamate. J Neurosci Res *72*, 191-197.
Jordan, J. D., He, J. C., Eungdamrong, N. J., Gomes, I., Ali, W., Nguyen, T., Bivona, T. G., Philips, M. R., Devi, L. A., and Iyengar, R. (2005). Cannabinoid receptor-induced neurite outgrowth is mediated by Rap1 activation through G(alpha)o/i-triggered proteasomal degradation of Rap1GAPII. J Biol Chem *280*, 11413-11421.
Kadota K, Nishimura S, Bono H, Nakamura S, Hayashizaki Y, Okazaki Y, Takahashi K. (2003). Detection of genes with tissue-specific expression patterns using Akaike's information criterion procedure. Physiol Genomics. *3*, 251-9.
Kaibuchi, K., Mizuno, T., Fujioka, H., Yamamoto, T., Kishi, K., Fukumoto, Y., Hori, Y., and Takai, Y. (1991). Molecular cloning of the cDNA for stimulatory GDP/GTP exchange protein for smg p21s (ras p21-like small GTP-binding proteins) and characterization of stimulatory GDP/GTP exchange protein. Mol Cell Biol *11*, 2873-2880.
Kawabe, H., Nakanishi, H., Asada, M., Fukuhara, A., Morimoto, K., Takeuchi, M., and Takai, Y. (2001). Pilt, a novel peripheral membrane protein at tight junctions in epithelial cells. J Biol Chem *276*, 48350-48355.
Kawahara, Y., Ito, K., Ito, M., Tsuji, S., and Kwak, S. (2005). Novel splice variants of human ADAR2 mRNA: skipping of the exon encoding the dsRNA-binding domains, and multiple C-terminal splice sites. Gene *363*, 193-201.
Kawamoto, S. (1996). Neuron-specific alternative splicing of nonmuscle myosin II heavy chain-B pre-mRNA requires a cis-acting intron sequence. J Biol Chem *271*, 17613-17616.
Kedra, D., Pan, H. Q., Seroussi, E., Fransson, I., Guilbaud, C., Collins, J. E., Dunham, I., Blennow, E., Roe, B. A., Piehl, F., and Dumanski, J. P. (1998). Characterization of the human synaptogyrin gene family. Hum Genet *103*, 131-141.
Kee, Y., Yoo, J. S., Hazuka, C. D., Peterson, K. E., Hsu, S. C., and Scheller, R. H. (1997). Subunit structure of the mammalian exocyst complex. Proc Natl Acad Sci U S A *94*, 14438-14443.
Keeling, S. L., Gad, J. M., and Cooper, H. M. (1997). Mouse Neogenin, a DCC-like molecule, has four splice variants and is expressed widely in the adult mouse and during embryogenesis. Oncogene *15*, 691-700.
Kempermann, G., Chesler, E. J., Lu, L., Williams, R. W., and Gage, F. H. (2006). Natural variation and genetic covariance in adult hippocampal neurogenesis. Proc Natl Acad Sci U S A *103*, 780-785.
Kessels, M. M., and Qualmann, B. (2002). Syndapins integrate N-WASP in receptor-mediated endocytosis. Embo J *21*, 6083-6094.
Kessels, M. M., and Qualmann, B. (2004). The syndapin protein family: linking membrane trafficking with the cytoskeleton. J Cell Sci *117*, 3077-3086.
Khanna, R., Chang, S. H., Andrabi, S., Azam, M., Kim, A., Rivera, A., Brugnara, C., Low, P. S., Liu, S. C., and Chishti, A. H. (2002). Headpiece domain of dematin is required for the stability of the erythrocyte membrane. Proc Natl Acad Sci U S A *99*, 6637-6642.
Kholmanskikh, S. S., Koeller, H. B., Wynshaw-Boris, A., Gomez, T., Letourneau, P. C., and Ross, M. E. (2006). Calcium-dependent interaction of Lis1 with IQGAP1 and Cdc42 promotes neuronal motility. Nat Neurosci *9*, 50-57.
Kim, A. C., Azim, A. C., and Chishti, A. H. (1998). Alternative splicing and structure of the human erythroid dematin gene. Biochim Biophys Acta *1398*, 382-386.
Kim, D., Jun, K. S., Lee, S. B., Kang, N. G., Min, D. S., Kim, Y. H., Ryu, S. H., Suh, P. G., and Shin, H. S. (1997a). Phospholipase C isozymes selectively couple to specific neurotransmitter receptors. Nature *389*, 290-293.
Kim, E., Lowenson, J. D., Clarke, S., and Young, S. G. (1999). Phenotypic analysis of seizure-prone mice lacking L-isoaspartate (D-aspartate) O-methyltransferase. J Biol Chem *274*, 20671-20678.
Kim, E., Lowenson, J. D., MacLaren, D. C., Clarke, S., and Young, S. G. (1997b). Deficiency of a protein-repair enzyme results in the accumulation of altered proteins, retardation of growth, and fatal seizures in mice. Proc Natl Acad Sci U S A *94*, 6132-6137.
Kim, E., and Sheng, M. (2004). PDZ domain proteins of synapses. Nat Rev Neurosci *5*, 771-781.
Kim, S., Ko, J., Shin, H., Lee, J. R., Lim, C., Han, J. H., Altrock, W. D., Garner, C. C., Gundelfinger, E. D., Premont, R. T., *et al.* (2003). The GIT family of proteins forms multimers and associates with the presynaptic cytomatrix protein Piccolo. J Biol Chem *278*, 6291-6300.
Knudsen, B. S., Feller, S. M., and Hanafusa, H. (1994). Four proline-rich sequences of the guanine-nucleotide exchange factor C3G bind with unique specificity to the first Src homology 3 domain of Crk. J Biol Chem *269*, 32781-32787.
Kong, H., Boulter, J., Weber, J. L., Lai, C., and Chao, M. V. (2001). An evolutionarily conserved transmembrane protein that is a novel downstream target of neurotrophin and ephrin receptors. J Neurosci *21*, 176-185.

Kotani, K., Kikuchi, A., Doi, K., Kishida, S., Sakoda, T., Kishi, K., and Takai, Y. (1992). The functional domain of the stimulatory GDP/GTP exchange protein (smg GDS) which interacts with the C-terminal geranylgeranylated region of rap1/Krev-1/smg p21. Oncogene *7*, 1699-1704.
Kuiperij, H. B., de Rooij, J., Rehmann, H., van Triest, M., Wittinghofer, A., Bos, J. L., and Zwartkruis, F. J. (2003). Characterisation of PDZ-GEFs, a family of guanine nucleotide exchange factors specific for Rap1 and Rap2. Biochim Biophys Acta *1593*, 141-149.
Lahuna, O., Quellari, M., Achard, C., Nola, S., Meduri, G., Navarro, C., Vitale, N., Borg, J. P., and Misrahi, M. (2005). Thyrotropin receptor trafficking relies on the hScrib-betaPIX-GIT1-ARF6 pathway. Embo J *24*, 1364-1374.
Lai, F., Chen, C. X., Carter, K. C., and Nishikura, K. (1997). Editing of glutamate receptor B subunit ion channel RNAs by four alternatively spliced DRADA2 double-stranded RNA adenosine deaminases. Mol Cell Biol *17*, 2413-2424.
Laura, R. P., Ross, S., Koeppen, H., and Lasky, L. A. (2002). MAGI-1: a widely expressed, alternatively spliced tight junction protein. Exp Cell Res *275*, 155-170.
Lee, H., Engel, U., Rusch, J., Scherrer, S., Sheard, K., and Van Vactor, D. (2004). The microtubule plus end tracking protein Orbit/MAST/CLASP acts downstream of the tyrosine kinase Abl in mediating axon guidance. Neuron *42*, 913-926.
Lee, S. H., Kim, M. H., Park, K. H., Earm, Y. E., and Ho, W. K. (2002). K+-dependent Na+/Ca2+ exchange is a major Ca2+ clearance mechanism in axon terminals of rat neurohypophysis. J Neurosci *22*, 6891-6899.
Leprince, C., Romero, F., Cussac, D., Vayssiere, B., Berger, R., Tavitian, A., and Camonis, J. H. (1997). A new member of the amphiphysin family connecting endocytosis and signal transduction pathways. J Biol Chem *272*, 15101-15105.
Leung, C. L., Zheng, M., Prater, S. M., and Liem, R. K. (2001). The BPAG1 locus: Alternative splicing produces multiple isoforms with distinct cytoskeletal linker domains, including predominant isoforms in neurons and muscles. J Cell Biol *154*, 691-697.
Li, L., and Chin, L. S. (2003). The molecular machinery of synaptic vesicle exocytosis. Cell Mol Life Sci *60*, 942-960.
Libby, R. T., Lillo, C., Kitamoto, J., Williams, D. S., and Steel, K. P. (2004). Myosin Va is required for normal photoreceptor synaptic activity. J Cell Sci *117*, 4509-4515.
Liu, J., Koyano-Nakagawa, N., Amasaki, Y., Saito-Ohara, F., Ikeuchi, T., Imai, S., Takano, T., Arai, N., Yokota, T., and Arai, K. (1997). Calcineurin-dependent nuclear translocation of a murine transcription factor NFATx: molecular cloning and functional characterization. Mol Biol Cell *8*, 157-170.
Liu, J. J., Ding, J., Kowal, A. S., Nardine, T., Allen, E., Delcroix, J. D., Wu, C., Mobley, W., Fuchs, E., and Yang, Y. (2003). BPAG1n4 is essential for retrograde axonal transport in sensory neurons. J Cell Biol *163*, 223-229.
Lu, C., Huang, X., Ma, H. F., Gooley, J. J., Aparacio, J., Roof, D. J., Chen, C., Chen, D. F., and Li, T. (2003). Normal retinal development and retinofugal projections in mice lacking the retina-specific variant of actin-binding LIM domain protein. Neuroscience *120*, 121-131.
Lundquist, E. A., Herman, R. K., Shaw, J. E., and Bargmann, C. I. (1998). UNC-115, a conserved protein with predicted LIM and actin-binding domains, mediates axon guidance in C. elegans. Neuron *21*, 385-392.
Luo, S., Chen, Y., Lai, K. O., Arevalo, J. C., Froehner, S. C., Adams, M. E., Chao, M. V., and Ip, N. Y. (2005). {alpha}-Syntrophin regulates ARMS localization at the neuromuscular junction and enhances EphA4 signaling in an ARMS-dependent manner. J Cell Biol *169*, 813-824.
Lutchman, M., Kim, A. C., Cheng, L., Whitehead, I. P., Oh, S. S., Hanspal, M., Boukharov, A. A., Hanada, T., and Chishti, A. H. (2002). Dematin interacts with the Ras-guanine nucleotide exchange factor Ras-GRF2 and modulates mitogen-activated protein kinase pathways. Eur J Biochem *269*, 638-649.
Maglott, D., Ostell, J., Pruitt, K. D., and Tatusova, T. (2005). Entrez Gene: gene-centered information at NCBI. Nucleic Acids Res *33*, D54-58.
Maragno, A. L., Baqui, M. M., and Gomes, M. D. (2006). FBXO25, an F-box protein homologue of atrogin-1, is not induced in atrophying muscle. Biochim Biophys Acta *1760*, 966-972.
Markovtsov, V., Nikolic, J. M., Goldman, J. A., Turck, C. W., Chou, M. Y., and Black, D. L. (2000). Cooperative assembly of an hnRNP complex induced by a tissue-specific homolog of polypyrimidine tract binding protein. Mol Cell Biol *20*, 7463-7479.
Marks, B., and McMahon, H. T. (1998). Calcium triggers calcineurin-dependent synaptic vesicle recycling in mammalian nerve terminals. Curr Biol *8*, 740-749.
Mateer, S. C., McDaniel, A. E., Nicolas, V., Habermacher, G. M., Lin, M. J., Cromer, D. A., King, M. E., and Bloom, G. S. (2002). The mechanism for regulation of the F-actin binding activity of IQGAP1 by calcium/calmodulin. J Biol Chem *277*, 12324-12333.
Mateer, S. C., Morris, L. E., Cromer, D. A., Bensenor, L. B., and Bloom, G. S. (2004). Actin filament binding by a monomeric IQGAP1 fragment with a single calponin homology domain. Cell Motil Cytoskeleton *58*, 231-241.
Matsunaga, E., Tauszig-Delamasure, S., Monnier, P. P., Mueller, B. K., Strittmatter, S. M., Mehlen, P., and Chedotal, A. (2004). RGM and its receptor neogenin regulate neuronal survival. Nat Cell Biol *6*, 749-755.
Meller, N., Irani-Tehrani, M., Kiosses, W. B., Del Pozo, M. A., and Schwartz, M. A. (2002). Zizimin1, a novel Cdc42 activator, reveals a new GEF domain for Rho proteins. Nat Cell Biol *4*, 639-647.

Meller, N., Irani-Tehrani, M., Ratnikov, B. I., Paschal, B. M., and Schwartz, M. A. (2004). The novel Cdc42 guanine nucleotide exchange factor, zizimin1, dimerizes via the Cdc42-binding CZH2 domain. J Biol Chem *279*, 37470-37476.

Meller, N., Merlot, S., and Guda, C. (2005). CZH proteins: a new family of Rho-GEFs. J Cell Sci *118*, 4937-4946.

Meyer, G., Varoqueaux, F., Neeb, A., Oschlies, M., and Brose, N. (2004). The complexity of PDZ domain-mediated interactions at glutamatergic synapses: a case study on neuroligin. Neuropharmacology *47*, 724-733.

Meyerhardt, J. A., Look, A. T., Bigner, S. H., and Fearon, E. R. (1997). Identification and characterization of neogenin, a DCC-related gene. Oncogene *14*, 1129-1136.

Mino, A., Ohtsuka, T., Inoue, E., and Takai, Y. (2000). Membrane-associated guanylate kinase with inverted orientation (MAGI)-1/brain angiogenesis inhibitor 1-associated protein (BAP1) as a scaffolding molecule for Rap small G protein GDP/GTP exchange protein at tight junctions. Genes Cells *5*, 1009-1016.

Minovitsky, S., Gee, S. L., Schokrpur, S., Dubchak, I., and Conboy, J. G. (2005). The splicing regulatory element, UGCAUG, is phylogenetically and spatially conserved in introns that flank tissue-specific alternative exons. Nucleic Acids Res *33*, 714-724.

Misaka, T., Miyashita, T., and Kubo, Y. (2002). Primary structure of a dynamin-related mouse mitochondrial GTPase and its distribution in brain, subcellular localization, and effect on mitochondrial morphology. J Biol Chem *277*, 15834-15842.

Miyata, M., Kashiwadani, H., Fukaya, M., Hayashi, T., Wu, D., Suzuki, T., Watanabe, M., and Kawakami, Y. (2003). Role of thalamic phospholipase C[beta]4 mediated by metabotropic glutamate receptor type 1 in inflammatory pain. J Neurosci *23*, 8098-8108.

Miyata, M., Kim, H. T., Hashimoto, K., Lee, T. K., Cho, S. Y., Jiang, H., Wu, Y., Jun, K., Wu, D., Kano, M., and Shin, H. S. (2001). Deficient long-term synaptic depression in the rostral cerebellum correlated with impaired motor learning in phospholipase C beta4 mutant mice. Eur J Neurosci *13*, 1945-1954.

Mizobuchi, M., Murao, K., Takeda, R., and Kakimoto, Y. (1994). Tissue-specific expression of isoaspartyl protein carboxyl methyltransferase gene in rat brain and testis. J Neurochem *62*, 322-328.

Mizuhara, E., Nakatani, T., Minaki, Y., Sakamoto, Y., Ono, Y., and Takai, Y. (2005). MAGI1 recruits Dll1 to cadherin-based adherens junctions and stabilizes it on the cell surface. J Biol Chem *280*, 26499-26507.

Mizuno, T., Kaibuchi, K., Yamamoto, T., Kawamura, M., Sakoda, T., Fujioka, H., Matsuura, Y., and Takai, Y. (1991). A stimulatory GDP/GTP exchange protein for smg p21 is active on the post-translationally processed form of c-Ki-ras p21 and rhoA p21. Proc Natl Acad Sci U S A *88*, 6442-6446.

Modregger, J., Ritter, B., Witter, B., Paulsson, M., and Plomann, M. (2000). All three PACSIN isoforms bind to endocytic proteins and inhibit endocytosis. J Cell Sci *113 Pt 24*, 4511-4521.

Monier, S., Jollivet, F., Janoueix-Lerosey, I., Johannes, L., and Goud, B. (2002). Characterization of novel Rab6-interacting proteins involved in endosome-to-TGN transport. Traffic *3*, 289-297.

Murdoch, J. N., Henderson, D. J., Doudney, K., Gaston-Massuet, C., Phillips, H. M., Paternotte, C., Arkell, R., Stanier, P., and Copp, A. J. (2003). Disruption of scribble (Scrb1) causes severe neural tube defects in the circletail mouse. Hum Mol Genet *12*, 87-98.

Nagao, T., Endo, K., Kawauchi, H., Walldorf, U., and Furukubo-Tokunaga, K. (2000). Patterning defects in the primary axonal scaffolds caused by the mutations of the extradenticle and homothorax genes in the embryonic Drosophila brain. Dev Genes Evol *210*, 289-299.

Nagata, K., Puls, A., Futter, C., Aspenstrom, P., Schaefer, E., Nakata, T., Hirokawa, N., and Hall, A. (1998). The MAP kinase kinase kinase MLK2 co-localizes with activated JNK along microtubules and associates with kinesin superfamily motor KIF3. Embo J *17*, 149-158.

Naisbitt, S., Valtschanoff, J., Allison, D. W., Sala, C., Kim, E., Craig, A. M., Weinberg, R. J., and Sheng, M. (2000). Interaction of the postsynaptic density-95/guanylate kinase domain-associated protein complex with a light chain of myosin-V and dynein. J Neurosci *20*, 4524-4534.

Nakahata, S., and Kawamoto, S. (2005). Tissue-dependent isoforms of mammalian Fox-1 homologs are associated with tissue-specific splicing activities. Nucleic Acids Res *33*, 2078-2089.

Nakata, T., Yokota, T., Emi, M., and Minami, S. (2002). Differential expression of multiple isoforms of the ELKS mRNAs involved in a papillary thyroid carcinoma. Genes Chromosomes Cancer *35*, 30-37.

Neudauer, C. L., Joberty, G., and Macara, I. G. (2001). PIST: a novel PDZ/coiled-coil domain binding partner for the rho-family GTPase TC10. Biochem Biophys Res Commun *280*, 541-547.

Neuman, T., Keen, A., Knapik, E., Shain, D., Ross, M., Nornes, H. O., and Zuber, M. X. (1993). ME1 and GE1: basic helix-loop-helix transcription factors expressed at high levels in the developing nervous system and in morphogenetically active regions. Eur J Neurosci *5*, 311-318.

Neuspiel, M., Zunino, R., Gangaraju, S., Rippstein, P., and McBride, H. (2005). Activated mitofusin 2 signals mitochondrial fusion, interferes with Bax activation, and reduces susceptibility to radical induced depolarization. J Biol Chem *280*, 25060-25070.

Nielsen, A. L., Pallisgaard, N., Pedersen, F. S., and Jorgensen, P. (1992). Murine helix-loop-helix transcriptional activator proteins binding to the E-box motif of the Akv murine leukemia virus enhancer identified by cDNA cloning. Mol Cell Biol *12*,

3449-3459.
Nishikimi, A., Meller, N., Uekawa, N., Isobe, K., Schwartz, M. A., and Maruyama, M. (2005). Zizimin2: a novel, DOCK180-related Cdc42 guanine nucleotide exchange factor expressed predominantly in lymphocytes. FEBS Lett *579*, 1039-1046.
Nuriya, M., Oh, S., and Huganir, R. L. (2005). Phosphorylation-dependent interactions of alpha-Actinin-1/IQGAP1 with the AMPA receptor subunit GluR4. J Neurochem *95*, 544-552.
Ogawara, M., Takahashi, M., Shimizu, T., Nakajima, M., Setoguchi, Y., and Shirasawa, T. (2002). Adenoviral expression of protein-L-isoaspartyl methyltransferase (PIMT) partially attenuates the biochemical changes in PIMT-deficient mice. J Neurosci Res *69*, 353-361.
Okumura, M., Yamakawa, H., Ohara, O., and Owaribe, K. (2002). Novel alternative splicings of BPAG1 (bullous pemphigoid antigen 1) including the domain structure closely related to MACF (microtubule actin cross-linking factor). J Biol Chem *277*, 6682-6687.
Orita, S., Kaibuchi, K., Kuroda, S., Shimizu, K., Nakanishi, H., and Takai, Y. (1993). Comparison of kinetic properties between two mammalian ras p21 GDP/GTP exchange proteins, ras guanine nucleotide-releasing factor and smg GDP dissociation stimulation. J Biol Chem *268*, 25542-25546.
Orth, J. D., and McNiven, M. A. (2003). Dynamin at the actin-membrane interface. Curr Opin Cell Biol *15*, 31-39.
Osterweil, E., Wells, D. G., and Mooseker, M. S. (2005). A role for myosin VI in postsynaptic structure and glutamate receptor endocytosis. J Cell Biol *168*, 329-338.
Oulad-Abdelghani, M., Chazaud, C., Bouillet, P., Sapin, V., Chambon, P., and Dolle, P. (1997). Meis2, a novel mouse Pbx-related homeobox gene induced by retinoic acid during differentiation of P19 embryonal carcinoma cells. Dev Dyn *210*, 173-183.
Pan, Q., Saltzman, A. L., Kim, Y. K., Misquitta, C., Shai, O., Maquat, L. E., Frey, B. J., and Blencowe, B. J. (2006). Quantitative microarray profiling provides evidence against widespread coupling of alternative splicing with nonsense-mediated mRNA decay to control gene expression. Genes Dev *20*, 153-158.
Pan, Q., Shai, O., Misquitta, C., Zhang, W., Saltzman, A. L., Mohammad, N., Babak, T., Siu, H., Hughes, T. R., Morris, Q. D., *et al.* (2004). Revealing global regulatory features of mammalian alternative splicing using a quantitative microarray platform. Mol Cell *16*, 929-941.
Parnis, A., Rawet, M., Regev, L., Barkan, B., Rotman, M., Gaitner, M., and Cassel, D. (2006). Golgi localization determinants in ArfGAP1 and in new tissue-specific ArfGAP1 isoforms. J Biol Chem *281*, 3785-3792.
Peters, M., Mizuno, K., Ris, L., Angelo, M., Godaux, E., and Giese, K. P. (2003). Loss of Ca2+/calmodulin kinase kinase beta affects the formation of some, but not all, types of hippocampus-dependent long-term memory. J Neurosci *23*, 9752-9760.
Pineda-Lucena, A., Ho, C. S., Mao, D. Y., Sheng, Y., Laister, R. C., Muhandiram, R., Lu, Y., Seet, B. T., Katz, S., Szyperski, T., *et al.* (2005). A structure-based model of the c-Myc/Bin1 protein interaction shows alternative splicing of Bin1 and c-Myc phosphorylation are key binding determinants. J Mol Biol *351*, 182-194.
Poinat, P., De Arcangelis, A., Sookhareea, S., Zhu, X., Hedgecock, E. M., Labouesse, M., and Georges-Labouesse, E. (2002). A conserved interaction between beta1 integrin/PAT-3 and Nck-interacting kinase/MIG-15 that mediates commissural axon navigation in C. elegans. Curr Biol *12*, 622-631.
Predescu, S. A., Predescu, D. N., Timblin, B. K., Stan, R. V., and Malik, A. B. (2003). Intersectin regulates fission and internalization of caveolae in endothelial cells. Mol Biol Cell *14*, 4997-5010.
Prekeris, R., and Terrian, D. M. (1997). Brain myosin V is a synaptic vesicle-associated motor protein: evidence for a Ca2+-dependent interaction with the synaptobrevin-synaptophysin complex. J Cell Biol *137*, 1589-1601.
Premont, R. T., Claing, A., Vitale, N., Perry, S. J., and Lefkowitz, R. J. (2000). The GIT family of ADP-ribosylation factor GTPase-activating proteins. Functional diversity of GIT2 through alternative splicing. J Biol Chem *275*, 22373-22380.
Pulst, S. M., Nechiporuk, A., Nechiporuk, T., Gispert, S., Chen, X. N., Lopes-Cendes, I., Pearlman, S., Starkman, S., Orozco-Diaz, G., Lunkes, A., *et al.* (1996). Moderate expansion of a normally biallelic trinucleotide repeat in spinocerebellar ataxia type 2. Nat Genet *14*, 269-276.
Qualmann, B., and Kelly, R. B. (2000). Syndapin isoforms participate in receptor-mediated endocytosis and actin organization. J Cell Biol *148*, 1047-1062.
Rajagopalan, S., Deitinghoff, L., Davis, D., Conrad, S., Skutella, T., Chedotal, A., Mueller, B. K., and Strittmatter, S. M. (2004). Neogenin mediates the action of repulsive guidance molecule. Nat Cell Biol *6*, 756-762.
Ramain, P., Khechumian, R., Khechumian, K., Arbogast, N., Ackermann, C., and Heitzler, P. (2000). Interactions between chip and the achaete/scute-daughterless heterodimers are required for pannier-driven proneural patterning. Mol Cell *6*, 781-790.
Ramjaun, A. R., and McPherson, P. S. (1998). Multiple amphiphysin II splice variants display differential clathrin binding: identification of two distinct clathrin-binding sites. J Neurochem *70*, 2369-2376.
Ramjaun, A. R., Micheva, K. D., Bouchelet, I., and McPherson, P. S. (1997). Identification and characterization of a nerve terminal-enriched amphiphysin isoform. J Biol Chem *272*, 16700-16706.
Ramjaun, A. R., Philie, J., de Heuvel, E., and McPherson, P. S. (1999). The N terminus of amphiphysin II mediates dimerization and plasma membrane targeting. J Biol Chem *274*, 19785-19791.

Rana, A. P., Ruff, P., Maalouf, G. J., Speicher, D. W., and Chishti, A. H. (1993). Cloning of human erythroid dematin reveals another member of the villin family. Proc Natl Acad Sci U S A *90*, 6651-6655.
Rebhun, J. F., Castro, A. F., and Quilliam, L. A. (2000). Identification of guanine nucleotide exchange factors (GEFs) for the Rap1 GTPase. Regulation of MR-GEF by M-Ras-GTP interaction. J Biol Chem *275*, 34901-34908.
Ren, M., Zeng, J., De Lemos-Chiarandini, C., Rosenfeld, M., Adesnik, M., and Sabatini, D. D. (1996). In its active form, the GTP-binding protein rab8 interacts with a stress-activated protein kinase. Proc Natl Acad Sci U S A *93*, 5151-5155.
Ritter, B., Modregger, J., Paulsson, M., and Plomann, M. (1999). PACSIN 2, a novel member of the PACSIN family of cytoplasmic adapter proteins. FEBS Lett *454*, 356-362.
Roof, D. J., Hayes, A., Adamian, M., Chishti, A. H., and Li, T. (1997). Molecular characterization of abLIM, a novel actin-binding and double zinc finger protein. J Cell Biol *138*, 575-588.
Roy, M., Li, Z., and Sacks, D. B. (2004). IQGAP1 binds ERK2 and modulates its activity. J Biol Chem *279*, 17329-17337.
Rueter, S. M., Dawson, T. R., and Emeson, R. B. (1999). Regulation of alternative splicing by RNA editing. Nature *399*, 75-80.
Sabo, S. L., Ikin, A. F., Buxbaum, J. D., and Greengard, P. (2003). The amyloid precursor protein and its regulatory protein, FE65, in growth cones and synapses in vitro and in vivo. J Neurosci *23*, 5407-5415.
Sakurai, A., Fukuhara, S., Yamagishi, A., Sako, K., Kamioka, Y., Masuda, M., Nakaoka, Y., and Mochizuki, N. (2006). MAGI-1 is required for Rap1 activation upon cell-cell contact and for enhancement of vascular endothelial cadherin-mediated cell adhesion. Mol Biol Cell *17*, 966-976.
Sanpei, K., Takano, H., Igarashi, S., Sato, T., Oyake, M., Sasaki, H., Wakisaka, A., Tashiro, K., Ishida, Y., Ikeuchi, T., *et al.* (1996). Identification of the spinocerebellar ataxia type 2 gene using a direct identification of repeat expansion and cloning technique, DIRECT. Nat Genet *14*, 277-284.
Saulnier, R., De Repentigny, Y., Yong, V. W., and Kothary, R. (2002). Alterations in myelination in the central nervous system of dystonia musculorum mice. J Neurosci Res *69*, 233-242.
Schafer, D. A. (2004). Regulating actin dynamics at membranes: a focus on dynamin. Traffic *5*, 463-469.
Schmitz, F. (2001). Immunological heterogeneity of synaptic and extrasynaptic forms of non-erythroid alpha-spectrin in the rat retina. Neurosci Lett *313*, 25-28.
Schwab, J. M., Guo, L., and Schluesener, H. J. (2005). Spinal cord injury induces early and persistent lesional P2X4 receptor expression. J Neuroimmunol *163*, 185-189.
Sergeeva, O. A., Amberger, B. T., Vorobjev, V. S., Eriksson, K. S., and Haas, H. L. (2004). AMPA receptor properties and coexpression with sodium-calcium exchangers in rat hypothalamic neurons. Eur J Neurosci *19*, 957-965.
Shai O, Morris QD, Blencowe BJ, Frey BJ. Inferring global levels of alternative splicing isoforms using a generative modelof microarray data. Bioinformatics. (2006) *22*:606-13
Shannon, P., Markiel, A., Ozier, O., Baliga, N. S., Wang, J. T., Ramage, D., Amin, N., Schwikowski, B., and Ideker, T. (2003). Cytoscape: a software environment for integrated models of biomolecular interaction networks. Genome Res *13*, 2498-2504.
Sharma, S., Dimasi, D., Higginson, K., and Della, N. G. (2004). RZF, a zinc-finger protein in the photoreceptors of human retina. Gene *342*, 219-229.
Shibata, A., Hattori, M., Suda, H., and Sakaki, Y. (1996). Identification of cis-acting elements involved in an alternative splicing of the amyloid precursor protein (APP) gene. Gene *175*, 203-208.
Shimizu, K., Kawabe, H., Minami, S., Honda, T., Takaishi, K., Shirataki, H., and Takai, Y. (1996). SMAP, an Smg GDS-associating protein having arm repeats and phosphorylated by Src tyrosine kinase. J Biol Chem *271*, 27013-27017.
Shirataki, H., Kaibuchi, K., Hiroyoshi, M., Isomura, M., Araki, S., Sasaki, T., and Takai, Y. (1991). Inhibition of the action of the stimulatory GDP/GTP exchange protein for smg p21 by the geranylgeranylated synthetic peptides designed from its C-terminal region. J Biol Chem *266*, 20672-20677.
Shiratsuchi, T., Futamura, M., Oda, K., Nishimori, H., Nakamura, Y., and Tokino, T. (1998). Cloning and characterization of BAI-associated protein 1: a PDZ domain-containing protein that interacts with BAI1. Biochem Biophys Res Commun *247*, 597-604.
Shivakrupa, Singh, R., and Swarup, G. (1999). Identification of a novel splice variant of C3G which shows tissue-specific expression. DNA Cell Biol *18*, 701-708.
Shukla, A., Corydon, T. J., Nielsen, S., Hoffmann, H. J., and Dahl, R. (2001). Identification of three new splice variants of the SNARE protein SNAP-23. Biochem Biophys Res Commun *285*, 320-327.
Siepel, A., Bejerano, G., Pedersen, J. S., Hinrichs, A. S., Hou, M., Rosenbloom, K., Clawson, H., Spieth, J., Hillier, L. W., Richards, S., *et al.* (2005). Evolutionarily conserved elements in vertebrate, insect, worm, and yeast genomes. Genome Res *15*, 1034-1050.
Slavov, D., and Gardiner, K. (2002). Phylogenetic comparison of the pre-mRNA adenosine deaminase ADAR2 genes and transcripts: conservation and diversity in editing site sequence and alternative splicing patterns. Gene *299*, 83-94.
Smillie, K. J., and Cousin, M. A. (2005). Dynamin I phosphorylation and the control of synaptic vesicle endocytosis. Biochem Soc Symp, 87-97.

Stamm, S., Casper, D., Hanson, V., and Helfman, D. M. (1999). Regulation of the neuron-specific exon of clathrin light chain B. Brain Res Mol Brain Res *64*, 108-118.
Stamm, S., Riethoven, J. J., Le Texier, V., Gopalakrishnan, C., Kumanduri, V., Tang, Y., Barbosa-Morais, N. L., and Thanaraj, T. A. (2006). ASD: a bioinformatics resource on alternative splicing. Nucleic Acids Res *34*, D46-55.
Stenius, K., Janz, R., Sudhof, T. C., and Jahn, R. (1995). Structure of synaptogyrin (p29) defines novel synaptic vesicle protein. J Cell Biol *131*, 1801-1809.
Struckhoff, E. C., and Lundquist, E. A. (2003). The actin-binding protein UNC-115 is an effector of Rac signaling during axon pathfinding in C. elegans. Development *130*, 693-704.
Sugita, S., Janz, R., and Sudhof, T. C. (1999). Synaptogyrins regulate Ca2+-dependent exocytosis in PC12 cells. J Biol Chem *274*, 18893-18901.
Sugnet, C. W., Srinivasan, K., Clark, T. A., O'Brien, G., Cline, M. S., Wang, H., Williams, A., Kulp, D., Blume, J. E., Haussler, D., and Ares, M. (2006). Unusual Intron Conservation near Tissue-Regulated Exons Found by Splicing Microarrays. PLoS Comput Biol *2*, e4.
Takakura, A., Miyoshi, J., Ishizaki, H., Tanaka, M., Togawa, A., Nishizawa, Y., Yoshida, H., Nishikawa, S., and Takai, Y. (2000). Involvement of a small GTP-binding protein (G protein) regulator, small G protein GDP dissociation stimulator, in antiapoptotic cell survival signaling. Mol Biol Cell *11*, 1875-1886.
Takechi, H., Hosokawa, N., Hirayoshi, K., and Nagata, K. (1994). Alternative 5' splice site selection induced by heat shock. Mol Cell Biol *14*, 567-575.
Takeda, S., Yamazaki, H., Seog, D. H., Kanai, Y., Terada, S., and Hirokawa, N. (2000). Kinesin superfamily protein 3 (KIF3) motor transports fodrin-associating vesicles important for neurite building. J Cell Biol *148*, 1255-1265.
Teng, J., Rai, T., Tanaka, Y., Takei, Y., Nakata, T., Hirasawa, M., Kulkarni, A. B., and Hirokawa, N. (2005). The KIF3 motor transports N-cadherin and organizes the developing neuroepithelium. Nat Cell Biol *7*, 474-482.
Toresson, H., Parmar, M., and Campbell, K. (2000). Expression of Meis and Pbx genes and their protein products in the developing telencephalon: implications for regional differentiation. Mech Dev *94*, 183-187.
Townsend-Nicholson, A., King, B. F., Wildman, S. S., and Burnstock, G. (1999). Molecular cloning, functional characterization and possible cooperativity between the murine P2X4 and P2X4a receptors. Brain Res Mol Brain Res *64*, 246-254.
Tran, Q., Coleman, T. P., and Roesser, J. R. (2003). Human transformer 2beta and SRp55 interact with a calcitonin-specific splice enhancer. Biochim Biophys Acta *1625*, 141-152.
Trevaskis, J., Walder, K., Foletta, V., Kerr-Bayles, L., McMillan, J., Cooper, A., Lee, S., Bolton, K., Prior, M., Fahey, R*., et al.* (2005). Src homology 3-domain growth factor receptor-bound 2-like (endophilin) interacting protein 1, a novel neuronal protein that regulates energy balance. Endocrinology *146*, 3757-3764.
Tsoi, M., Rhee, K. H., Bungard, D., Li, X. F., Lee, S. L., Auer, R. N., and Lytton, J. (1998). Molecular cloning of a novel potassium-dependent sodium-calcium exchanger from rat brain. J Biol Chem *273*, 4155-4162.
Tsuda, M., Shigemoto-Mogami, Y., Koizumi, S., Mizokoshi, A., Kohsaka, S., Salter, M. W., and Inoue, K. (2003). P2X4 receptors induced in spinal microglia gate tactile allodynia after nerve injury. Nature *424*, 778-783.
Tsutsui, K., Maeda, Y., Seki, S., and Tokunaga, A. (1997). cDNA cloning of a novel amphiphysin isoform and tissue-specific expression of its multiple splice variants. Biochem Biophys Res Commun *236*, 178-183.
Uittenbogaard, M., and Chiaramello, A. (1999). Expression of the basic Helix-Loop-Helix ME1 E-protein during development and aging of the murine cerebellum. Neurosci Lett *274*, 191-194.
Uittenbogaard, M., and Chiaramello, A. (2002). Expression of the bHLH transcription factor Tcf12 (ME1) gene is linked to the expansion of precursor cell populations during neurogenesis. Brain Res Gene Expr Patterns *1*, 115-121.
Ule, J., Ule, A., Spencer, J., Williams, A., Hu, J. S., Cline, M., Wang, H., Clark, T., Fraser, C., Ruggiu, M*., et al.* (2005). Nova regulates brain-specific splicing to shape the synapse. Nat Genet *37*, 844-852.
Underwood, J. G., Boutz, P. L., Dougherty, J. D., Stoilov, P., and Black, D. L. (2005). Homologues of the Caenorhabditis elegans Fox-1 protein are neuronal splicing regulators in mammals. Mol Cell Biol *25*, 10005-10016.
Usener, D., Schadendorf, D., Koch, J., Dubel, S., and Eichmuller, S. (2003). cTAGE: a cutaneous T cell lymphoma associated antigen family with tumor-specific splicing. J Invest Dermatol *121*, 198-206.
Vaessin, H., Brand, M., Jan, L. Y., and Jan, Y. N. (1994). daughterless is essential for neuronal precursor differentiation but not for initiation of neuronal precursor formation in Drosophila embryo. Development *120*, 935-945.
Vailaya, A., Bluvas, P., Kincaid, R., Kuchinsky, A., Creech, M., and Adler, A. (2005). An architecture for biological information extraction and representation. Bioinformatics *21*, 430-438.
Vallano, M. L., Beaman-Hall, C. M., Mathur, A., and Chen, Q. (2000). Astrocytes express specific variants of CaM KII delta and gamma, but not alpha and beta, that determine their cellular localizations. Glia *30*, 154-164.
Van Lieshout, E. M., Van der Heijden, I., Hendriks, W. J., and Van der Zee, C. E. (2001). A decrease in size and number of basal forebrain cholinergic neurons is paralleled by diminished hippocampal cholinergic innervation in mice lacking leukocyte common antigen-related protein tyrosine phosphatase activity. Neuroscience *102*, 833-841.
Venables W.N. and Ripley B.D. (2002). Modern Applied Statistics with S. Fourth Edition Springer.

Verma, R., Chauhan, C., Saleem, Q., Gandhi, C., Jain, S., and Brahmachari, S. K. (2004). A nonsense mutation in the synaptogyrin 1 gene in a family with schizophrenia. Biol Psychiatry *55*, 196-199.
Verma, R., Kubendran, S., Das, S. K., Jain, S., and Brahmachari, S. K. (2005). SYNGR1 is associated with schizophrenia and bipolar disorder in southern India. J Hum Genet *50*, 635-640.
Vielmetter, J., Kayyem, J. F., Roman, J. M., and Dreyer, W. J. (1994). Neogenin, an avian cell surface protein expressed during terminal neuronal differentiation, is closely related to the human tumor suppressor molecule deleted in colorectal cancer. J Cell Biol *127*, 2009-2020.
Walsh, D., Li, Z., Wu, Y., and Nagata, K. (1997). Heat shock and the role of the HSPs during neural plate induction in early mammalian CNS and brain development. Cell Mol Life Sci *53*, 198-211.
Wang, B., Hu, Q., Hearn, M. G., Shimizu, K., Ware, C. B., Liggitt, D. H., Jin, L. W., Cool, B. H., Storm, D. R., and Martin, G. M. (2004a). Isoform-specific knockout of FE65 leads to impaired learning and memory. J Neurosci Res *75*, 12-24.
Wang, Y., Liu, X., Biederer, T., and Sudhof, T. C. (2002). A family of RIM-binding proteins regulated by alternative splicing: Implications for the genesis of synaptic active zones. Proc Natl Acad Sci U S A *99*, 14464-14469.
Wang, Z., Rolish, M. E., Yeo, G., Tung, V., Mawson, M., and Burge, C. B. (2004b). Systematic identification and analysis of exonic splicing silencers. Cell *119*, 831-845.
Washbourne, P., Liu, X. B., Jones, E. G., and McAllister, A. K. (2004). Cycling of NMDA receptors during trafficking in neurons before synapse formation. J Neurosci *24*, 8253-8264.
Watakabe, A., Kobayashi, R., and Helfman, D. M. (1996). N-tropomodulin: a novel isoform of tropomodulin identified as the major binding protein to brain tropomyosin. J Cell Sci *109 (Pt 9)*, 2299-2310.
Watanabe, M., Nomura, K., Ohyama, A., Ishikawa, R., Komiya, Y., Hosaka, K., Yamauchi, E., Taniguchi, H., Sasakawa, N., Kumakura, K.*, et al.* (2005). Myosin-Va regulates exocytosis through the submicromolar Ca2+-dependent binding of syntaxin-1A. Mol Biol Cell *16*, 4519-4530.
Watanabe, T., Wang, S., Noritake, J., Sato, K., Fukata, M., Takefuji, M., Nakagawa, M., Izumi, N., Akiyama, T., and Kaibuchi, K. (2004). Interaction with IQGAP1 links APC to Rac1, Cdc42, and actin filaments during cell polarization and migration. Dev Cell *7*, 871-883.
Wechsler-Reya, R., Sakamuro, D., Zhang, J., Duhadaway, J., and Prendergast, G. C. (1997). Structural analysis of the human BIN1 gene. Evidence for tissue-specific transcriptional regulation and alternate RNA splicing. J Biol Chem *272*, 31453-31458.
Wheeler, D. L., Barrett, T., Benson, D. A., Bryant, S. H., Canese, K., Chetvernin, V., Church, D. M., DiCuccio, M., Edgar, R., Federhen, S.*, et al.* (2006). Database resources of the National Center for Biotechnology Information. Nucleic Acids Res *34*, D173-180.
Wightman, B., Baran, R., and Garriga, G. (1997). Genes that guide growth cones along the C. elegans ventral nerve cord. Development *124*, 2571-2580.
Wright, G. J., Leslie, J. D., Ariza-McNaughton, L., and Lewis, J. (2004). Delta proteins and MAGI proteins: an interaction of Notch ligands with intracellular scaffolding molecules and its significance for zebrafish development. Development *131*, 5659-5669.
Wu, C., Lai, C. F., and Mobley, W. C. (2001). Nerve growth factor activates persistent Rap1 signaling in endosomes. J Neurosci *21*, 5406-5416.
Wu, H., Nash, J. E., Zamorano, P., and Garner, C. C. (2002). Interaction of SAP97 with minus-end-directed actin motor myosin VI. Implications for AMPA receptor trafficking. J Biol Chem *277*, 30928-30934.
Wu, Y., Dowbenko, D., Spencer, S., Laura, R., Lee, J., Gu, Q., and Lasky, L. A. (2000). Interaction of the tumor suppressor PTEN/MMAC with a PDZ domain of MAGI3, a novel membrane-associated guanylate kinase. J Biol Chem *275*, 21477-21485.
Xie, J., Jan, C., Stoilov, P., Park, J., and Black, D. L. (2005). A consensus CaMK IV-responsive RNA sequence mediates regulation of alternative exons in neurons. Rna *11*, 1825-1834.
Xu, X., Yang, D., Ding, J. H., Wang, W., Chu, P. H., Dalton, N. D., Wang, H. Y., Bermingham, J. R., Jr., Ye, Z., Liu, F.*, et al.* (2005). ASF/SF2-regulated CaMKIIdelta alternative splicing temporally reprograms excitation-contraction coupling in cardiac muscle. Cell *120*, 59-72.
Yamamoto, A., Takagi, H., Kitamura, D., Tatsuoka, H., Nakano, H., Kawano, H., Kuroyanagi, H., Yahagi, Y., Kobayashi, S., Koizumi, K.*, et al.* (1998). Deficiency in protein L-isoaspartyl methyltransferase results in a fatal progressive epilepsy. J Neurosci *18*, 2063-2074.
Yamashita, T., Hige, T., and Takahashi, T. (2005). Vesicle endocytosis requires dynamin-dependent GTP hydrolysis at a fast CNS synapse. Science *307*, 124-127.
Yamazaki, H., Nakata, T., Okada, Y., and Hirokawa, N. (1996). Cloning and characterization of KAP3: a novel kinesin superfamily-associated protein of KIF3A/3B. Proc Natl Acad Sci U S A *93*, 8443-8448.
Yang, J. H., Sklar, P., Axel, R., and Maniatis, T. (1997). Purification and characterization of a human RNA adenosine deaminase for glutamate receptor B pre-mRNA editing. Proc Natl Acad Sci U S A *94*, 4354-4359.
Yang, L., Sym, M., and Kenyon, C. (2005). The roles of two C. elegans HOX co-factor orthologs in cell migration and vulva development. Development *132*, 1413-1428.

Yang, Y., Bauer, C., Strasser, G., Wollman, R., Julien, J. P., and Fuchs, E. (1999). Integrators of the cytoskeleton that stabilize microtubules. Cell *98*, 229-238.
Yang, Y., Dowling, J., Yu, Q. C., Kouklis, P., Cleveland, D. W., and Fuchs, E. (1996). An essential cytoskeletal linker protein connecting actin microfilaments to intermediate filaments. Cell *86*, 655-665.
Yang, Y., Hwang, C. K., D'Souza, U. M., Lee, S. H., Junn, E., and Mouradian, M. M. (2000). Three-amino acid extension loop homeodomain proteins Meis2 and TGIF differentially regulate transcription. J Biol Chem *275*, 20734-20741.
Yang, Y., and Lundquist, E. A. (2005). The actin-binding protein UNC-115/abLIM controls formation of lamellipodia and filopodia and neuronal morphogenesis in Caenorhabditis elegans. Mol Cell Biol *25*, 5158-5170.
York, R. D., Yao, H., Dillon, T., Ellig, C. L., Eckert, S. P., McCleskey, E. W., and Stork, P. J. (1998). Rap1 mediates sustained MAP kinase activation induced by nerve growth factor. Nature *392*, 622-626.
Yoshimura, Y., Aoi, C., and Yamauchi, T. (2000). Investigation of protein substrates of Ca(2+)/calmodulin-dependent protein kinase II translocated to the postsynaptic density. Brain Res Mol Brain Res *81*, 118-128.
Yu, W., Sharp, D. J., Kuriyama, R., Mallik, P., and Baas, P. W. (1997). Inhibition of a mitotic motor compromises the formation of dendrite-like processes from neuroblastoma cells. J Cell Biol *136*, 659-668.
Yue, Z., Horton, A., Bravin, M., DeJager, P. L., Selimi, F., and Heintz, N. (2002). A novel protein complex linking the delta 2 glutamate receptor and autophagy: implications for neurodegeneration in lurcher mice. Neuron *35*, 921-933.
Zhai, B., Huo, H., and Liao, K. (2001). C3G, a guanine nucleotide exchange factor bound to adapter molecule c-Crk, has two alternative splicing forms. Biochem Biophys Res Commun *286*, 61-66.
Zhang, H., Webb, D. J., Asmussen, H., Niu, S., and Horwitz, A. F. (2005). A GIT1/PIX/Rac/PAK signaling module regulates spine morphogenesis and synapse formation through MLC. J Neurosci *25*, 3379-3388.
Zhang, J. S., Honkaniemi, J., Yang, T., Yeo, T. T., and Longo, F. M. (1998). LAR Tyrosine Phosphatase Receptor: A Developmental Isoform Is Present in Neurites and Growth Cones and Its Expression Is Regional- and Cell-Specific. Mol Cell Neurosci *10*, 271-286.
Zhang, J. S., and Longo, F. M. (1995). LAR tyrosine phosphatase receptor: alternative splicing is preferential to the nervous system, coordinated with cell growth and generates novel isoforms containing extensive CAG repeats. J Cell Biol *128*, 415-431.
Zhang, L., Ashiya, M., Sherman, T. G., and Grabowski, P. J. (1996). Essential nucleotides direct neuron-specific splicing of gamma 2 pre-mRNA. Rna *2*, 682-698.
Zhang, Q., Ragnauth, C., Greener, M. J., Shanahan, C. M., and Roberts, R. G. (2002). The nesprins are giant actin-binding proteins, orthologous to Drosophila melanogaster muscle protein MSP-300. Genomics *80*, 473-481.
Zhang, X. H., and Chasin, L. A. (2004). Computational definition of sequence motifs governing constitutive exon splicing. Genes Dev *18*, 1241-1250.
Zhao, W., and Manley, J. L. (1996). Complex alternative RNA processing generates an unexpected diversity of poly(A) polymerase isoforms. Mol Cell Biol *16*, 2378-2386.

ADDITIONAL DATA FILE 1 FIGURE LEGENDS

Additional Data File 2: Information on tissue-specific AS events.

The columns are labeled as follows: Id, Array event Identifier; accession, GenBank accession id; gene, original Unigene cluster used; UGCluster, updated Unigene cluster; Name, long form description of the gene name; Symbol: The NCBI Gene Symbol Pattern; the tissue-specific AS pattern associated with the respective tissues listed. The label "CNS-Cotranscriptional" denotes events which have significant CNS-specific AS change p-values using a model that does not take transcript levels into account but are not found to have significant p-values when transcript levels are taken into account.

Additional Data File 3: Information on CNS-regulated AS events.

A more detailed version of Table 1 (main text) indicating relevant information on individual AS events in the published literature, and the corresponding references (included above).

Additional Data File 4: Information on correlated pairs of AS events belonging to the same genes.

Each row contains data for an exon pair. The columns are labeled as follows: Gene_id, Unigene identifier for the gene; Accession, Genbank accession number used; Spearman_normal_cor, the standard Spearman correlation of AS levels; Spearman_partial_cor, the partial Spearman correlation with respect to transcript levels; Lower partial correlation indicates that transcript levels are correlating with AS levels; GeneName, the long description of the gene name; Symbol, the standard NCBI Gene symbol for the genes; num_obs, the number of tissues with transcript levels above the 95th percentile of the negative control probes; dist_between, the genomic distance between the exons; num_exons_between, the number of exons separating the exon pair; length_exons_between, the nucleotide length of all the exons separating the exon pair.

Additional Data File 5: Motifs associated with CNS-specific AS events detected from *ab initio* searches.

The following terminology/abbreviations are used:

Group

Refers to the group a sequence/motif is correlated with. The following groups were defined:

CNSex - The group of alternative exons displaying preferential exclusion in CNS tissues

CNSin - The group of alternative exons displaying preferential inclusion in CNS tissues

CNSch - The group of alternative exons displaying preferential inclusion or exclusion in CNS tissues.

Location

Refers to the exonic and intronic sequence region(s) a motif is found to be significantly enriched in. The following locations were searched:

A - The alternative exon

C1 - The constitutive exon upstream of the alternative exon

C2 - The constitutive exon downstream of the alternative exon

AI1 - The 150 nt intronic region upstream of the alternative exon

AI2 - The 150 nt intronic region downstream of the alternative exon

CII1 - The 150 nt intronic region downstream of the C1 exon

C2I2 - The 150 nt intronic region upstream of the C2 exon

"-" - Indicates a concatenation (with a 20 nt spacer) of two of the regions listed above.

Score

Hypergeometric p-values are given The second number is the original p-value (-log10 transformed) and the first is the corrected p-value (also –log10 transformed), as described above in the Materials and Methods.

ScoreParams:

Gives the parameters for the hypergeometric-based score (see Materials and Methods).

X - the number of sequences from the "positive" group (i.e. number of CNS–regulated AS event sequences from the Up, Down or Change groups) found to have the motif

N - the number of events in the "positive" group.

M - the total number of events (both "positive" and "negative" groups).

K - the total number of events found to have the motifs (in either the "positive" or the "negative" group).

KnownCompare:

This column list the known motifs that were found to be similar to each motif.

It includes the best alignment of the two motifs, the estimated p-value of the alignment score and references to the relevant literature (see Materials and Methods)

Additional Data File 6: Experimentally-defined sequences/motifs associated with neural-specific AS used for searches (refer to Materials and Methods above for details).

Additional Data File 7: Motifs associated with CNS-specific AS events detected from searches using

subsequences of known motifs.
Experimentally-defined motifs/subsequences significantly enriched in exons and introns associated with CNS-regulated AS events identified in the AS microarray data (refer to Materials and Methods). Experimentally defined sequences were broken down into overlapping 7 to 10 nt subsequences, and these were used in the searches. The "Motif/Subseq" column shows the search subsequence. The "Description" column shows the name of the splicing factor (if known; refer to Table S5A) following by the notation: _x_y, where x indicates the length of the subsequence and y indicates the location of the subsequence within a longer sequence. All possible subsequence of length x and overlapping by 1 nt were used in the searches.
The following abbreviations are used:
Group - Refers to the group a sequence/motif is correlated with. The following groups were defined:
CNSex - The group of alternative exons displaying preferential exclusion in CNS tissues
CNSin - The group of alternative exons displaying preferential inclusion in CNS tissues
CNSch - The group of alternative exons displaying preferential inclusion or exclusion in CNS tissues.
Location – Refers to the exonic and intronic sequence region(s) a motif is found to be significantly enriched in. The following locations were searched:
A - The alternative exon
C1 - The constitutive exon upstream of the alternative exon
C2 - The constitutive exon downstream of the alternative exon
AI1 - The 150 nt intronic region upstream of the alternative exon
AI2 - The 150 nt intronic region downstream of the alternative exon
CII1 - The 150 nt intronic region downstream of the C1 exon
C2I2 - The 150 nt intronic region upstream of the C2 exon
"-" - Indicates a concatenation (with a 20 nt spacer) of two of the regions listed above.
hgPval - Indicates the hypergeometric p-value of the motif (refer to Materials and Methods above) in the specified group and location.

Additional Data File 8: Number and statistical significance of *ab initio* motifs detected at each exonic and intronic location, and in each group.
Locations and groups with the highest numbers of statistically significant motifs are highlighted in yellow. Abbreviations are as listed above in the Table S5 legend.

Additional Data File 9: Motifs associated with CNS-specific AS events detected by searching with the Improbizer program.
Abbreviations are as listed above in the Table S5 legend.

Additional Data File 10: Conservation levels of motifs associated with CNS-specific AS events detected from *ab initio* searches
The Following abbreviations are used:
Grp – The groups as defined in Table S5 legend.
Seq - The sequence regions as defined in Table S5 legend.
Pos – The number of events where the motif was found in the "positive" group matching the definition in "Grp".
PosC – The number of conserved "positive" motif occurrences, i.e. when the motif found in a sequence from the "positive" group and was also found in the corresponding region in the orthologous human gene.
Neg – The number of events where the motif was found in the "negative" group matching the definition

in "Grp".
NegC – The number of conserved "negative" motif occurrences, i.e. when the motif was found in a sequence from the "negative" group and was also found in its orthologous human sequence.
RatioP – (PocC/Pos) The ratio of conserved motif occurrences in the "positive" group matching the definition in "Grp".
RatioN – (NecC/Nec) The ratio of conserved motif occurrences in the "negative" group matching the definition in "Grp".
RatioDiff – (RatioP – RatioN) The difference in conservation ratio of the motif between the "positive" and "negative" groups matching the definition in "Grp". Colored indicates a higher conservation in the "positive" set .
RatioRandPos – The average ratio of conservation of random motifs, of the same complexity as the given motif, in the "positive" group matching the definition in "Grp".
RatioRandNeg – The average ratio of conservation of random motifs, of the same complexity as the given motif, in the "negative" group matching the definition in "Grp".
RatioRandDiff – (RatioRandPos – RatioRandNeg). The difference in average conservation ratio of random motif between the "positive" and "negative" groups matching the definition in "Grp". Colored indicates a higher conservation in the "positive" set .
-log10(pvalPC) – The statistical significance of the conservation ratio of the motif in the "positive" group matching the definition in "Grp", based on the Binomial tail distribution. P-values are –log10 transformed. P-values less then 0.05 (i.e. larger then 1.31 after transformation) are colored.

ADDITIONAL DATA FILE FIGURE LEGENDS

Figure 1. Correlation between GenASAP and RT-PCR values for percent exon exclusion levels of correlated pairs of AS events.
GenASAP values and RT-PCR measurements for percent exon exclusion levels were compared for exons belonging to pairs of correlated exons, for six pairs of correlated AS events across ten mouse tissues (120 independent measurements). An overall Pearson correlation coefficient >8.0 was observed.

Figure 2. RT-PCR analysis of pairs of AS events belonging to the same genes with predicted correlated exclusion levels.
Refer to Figure 2 (main text) legend.

Figure 3. Analysis of exon counts and nucleotide distances between pairs of exons belonging to the same genes that display correlated splicing patterns.

(A) Empirical cumulative distribution function plot of the proportion of alternative exon pairs with intervening exon counts that are less than or equal to a given value on the x-axis.

(B) Empirical cumulative distribution function plot of the proportion of alternative exon pairs with intervening exon lengths (in nucleotides) that are less than or equal to a given value on the x-axis.

(C) Empirical cumulative distribution function plot of the proportion of alternative exon pairs with intervening pre-mRNA lengths (or genomic distances, in nucleotides) that are less than or equal to a given value on the x-axis.

In all plots, the three curves show the group without high correlation (-0.6 < Correlation < 0.6, black, n=564), the group with high negative correlation (Correlation <= -0.6, red, n=16), and the group with high positive correlation (Correlation >= 0.6, blue, n=22), where Correlation is the standard Spearman correlation.

Figure 4. Analysis of microarray profiled genes with CNS-specific transcript levels
(A) Heatmap showing genes with the top 100-most significant transcript level differences between CNS and non-CNS tissues, in which the rows and columns have been 2-dimensionally hierarchically clustered. Increasingly bright yellow represents lower transcript levels, and increasingly bright cyan blue represents higher transcript level. The range of values is from 4 to 9.5 on the arcsinh scale. CNS tissues are indicated.
(B) AS levels detected in the same set of genes as shown in (A). The order of the rows and columns are the same as in (A).

Figure 5. Conservation analysis of intronic regions flanking CNS-specific AS event exons.
Empirical cumulative distribution function plots of the numbers of nucleotides in a given intronic region that overlap with phastCons Most Conserved elements which are among the most highly conserved regions in several vertebrate genomes (Siepel et al., 2005) are shown. The y-axis represents the number of all AS events in a group that have less than or equal to a given number of nucleotides present in the Most Conserved elements as shown on the x-axis. The plots for the CNS-specific AS events are shown in blue with closed circles, and the plots for the other events are shown in red with open circles.
(a) The plot for the intronic region from 1 to 50 nt upstream of the exon.
(b) The plot for the intronic region from 1 to 50 nt downstream of the exon.
(c) The plot for the intronic region from 51 to 100 nt upstream of the exon.
(d) The plot for the intronic region from 51 to 100 nt downstream of the exon.
(e) The plot for the intronic region from 101 to 150 nt upstream of the exon.
(f) The plot for the intronic region from 101 to 150 nt downstream of the exon.

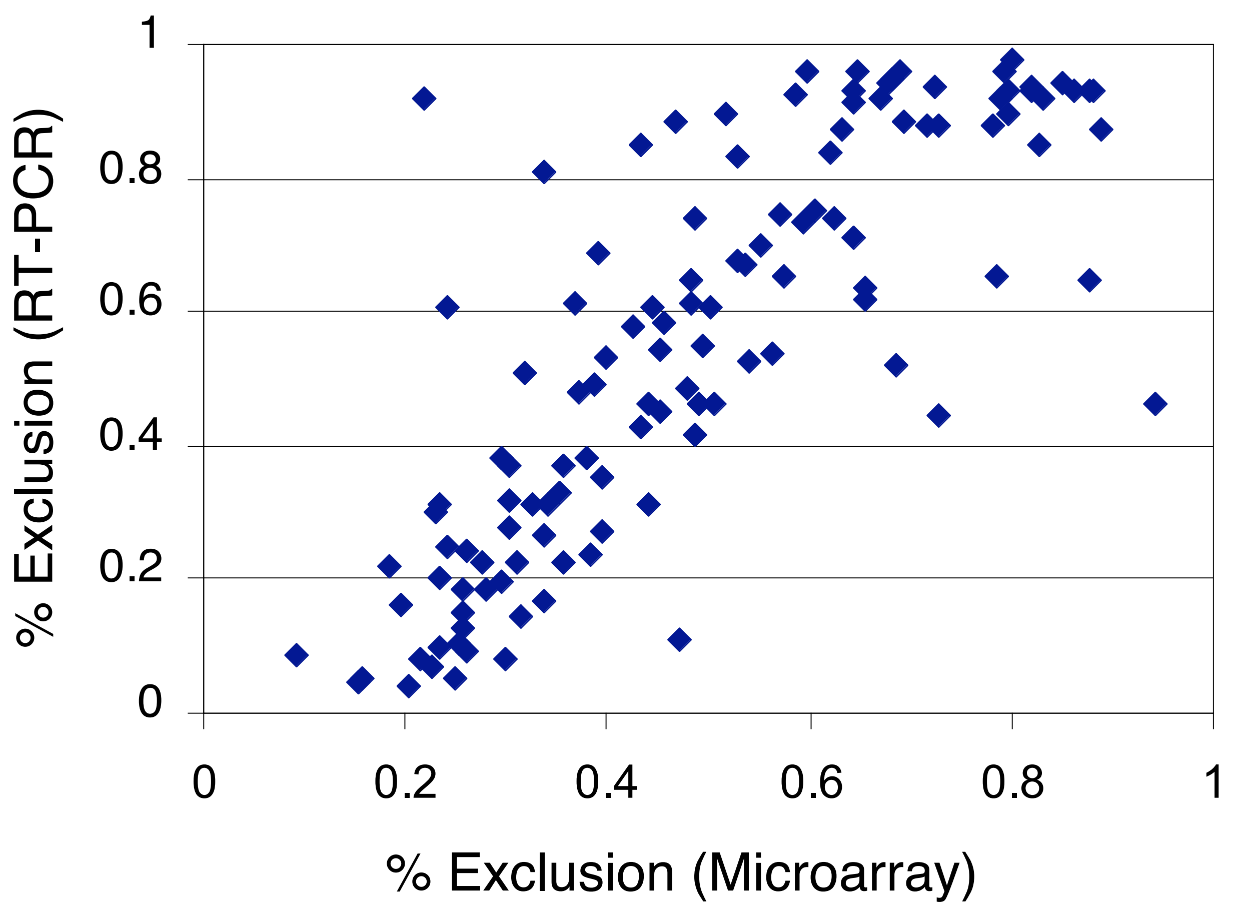

Fagnani/Barash et al. Figure S1

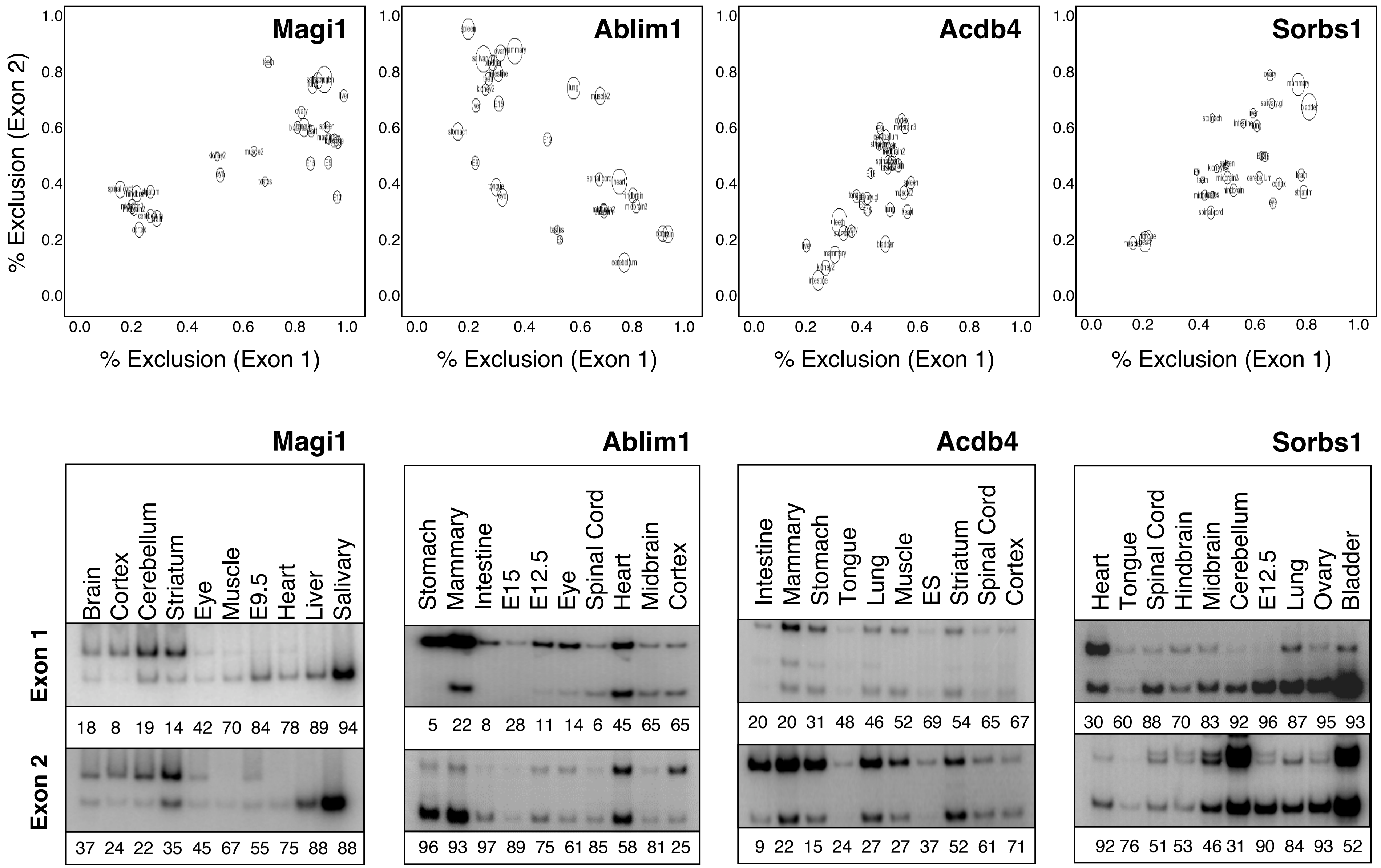

Fagnani/Barash et al. Figure S2

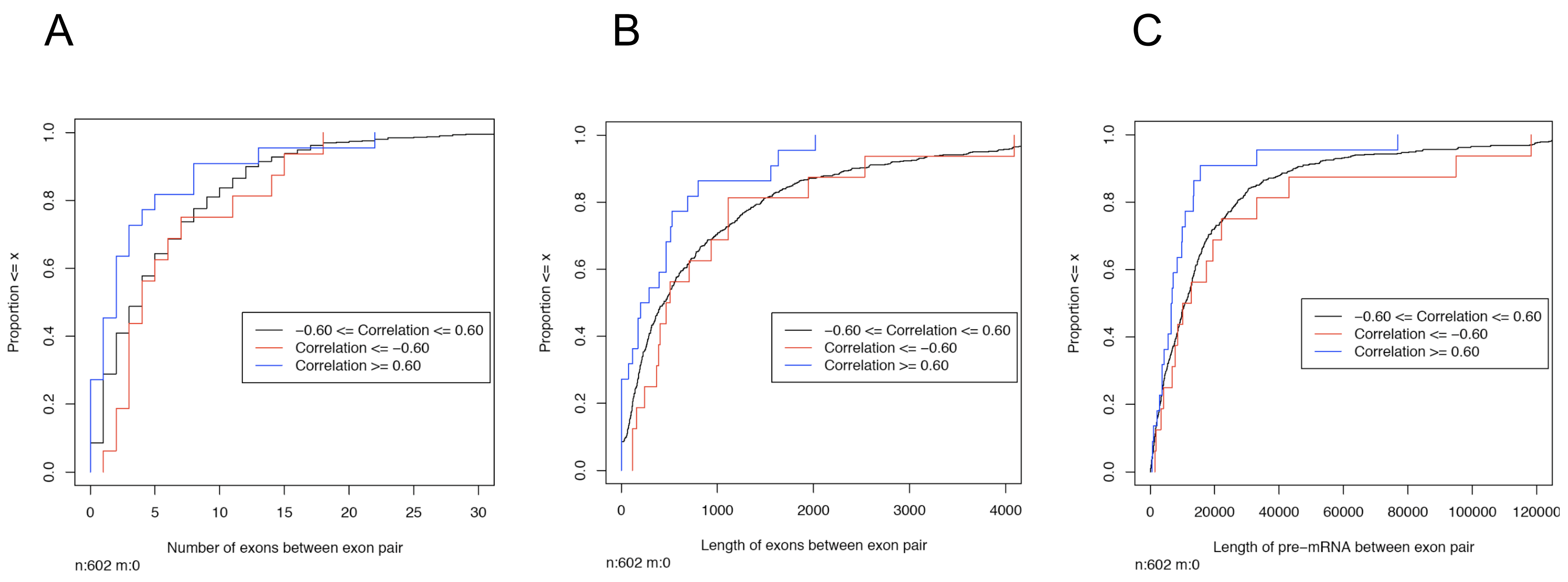

Fagnani/Barash et al. Figure S3

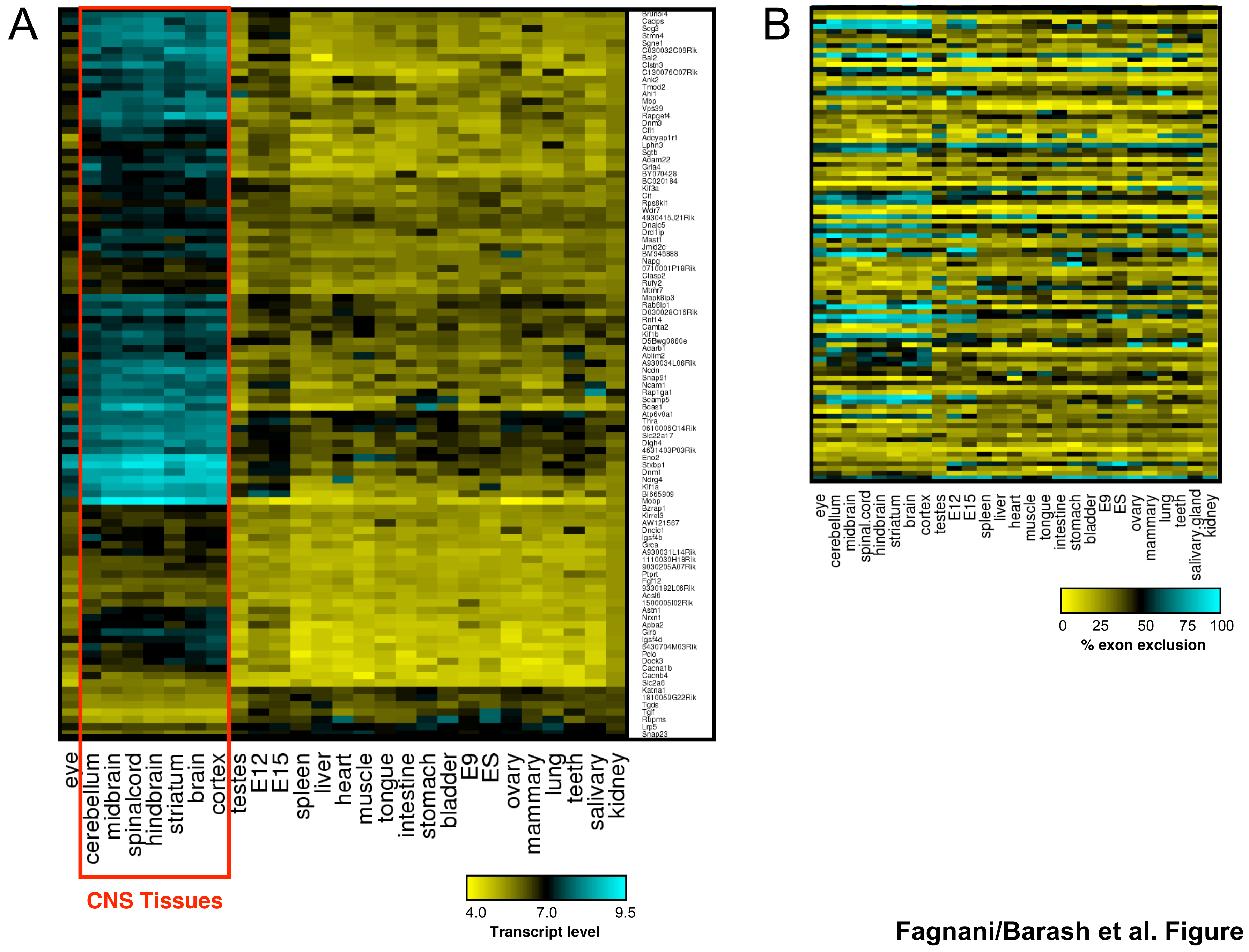

Fagnani/Barash et al. Figure S4

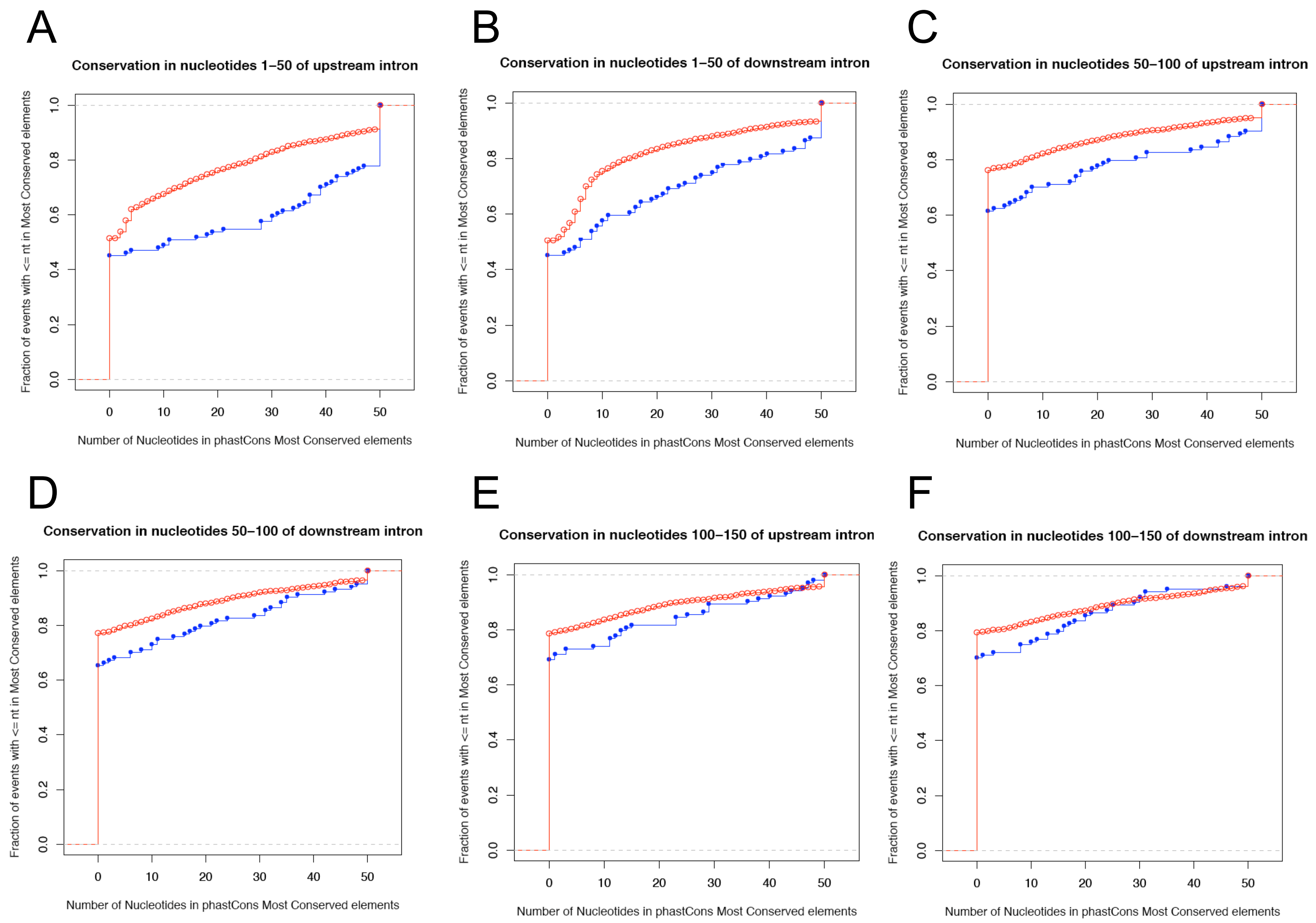
A
Conservation in nucleotides 1–50 of upstream intron
B
Conservation in nucleotides 1–50 of downstream intron
C
Conservation in nucleotides 50–100 of upstream intron
D
Conservation in nucleotides 50–100 of downstream intron
E
Conservation in nucleotides 100–150 of upstream intron
F
Conservation in nucleotides 100–150 of downstream intron
Fraction of events with <= nt in Most Conserved elements
Number of Nucleotides in phastCons Most Conserved elements
0.0
0.2
0.4
0.6
0.8
1.0
0
10
20
30
40
50

Figure S5
