# Supplementary material for: Functional coordination of alternative splicing in the mammalian central nervous system
Source: Genome Biol. 2007 Jun 12;8(6):R108. doi: 10.1186/gb-2007-8-6-r108 (PMC2394768; doi:10.1186/gb-2007-8-6-r108)
Supplement: Additional data file 9 — Summarized are motifs associated with CNS specific AS events detected by searching with the Improbizer program. [file gb-2007-8-6-r108-S9.pdf]

Additional Data File 9: Motifs associated with CNS-specific AS events detected by searching with the Improbizer program

SeqArea	CNSex	CNSin	CNSch
A	0	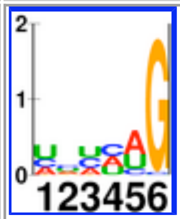	0
A_C2	0	0	0
C1I1	0	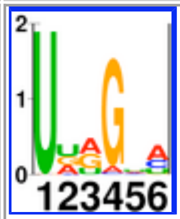	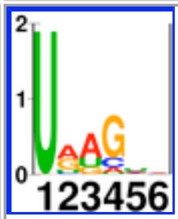
C1I1_C2I2	0	0	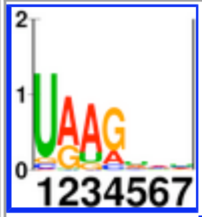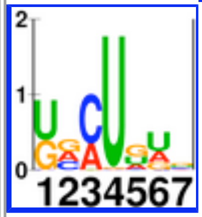
C1_C2	0	0	0
C2I2	0	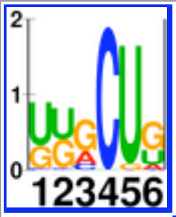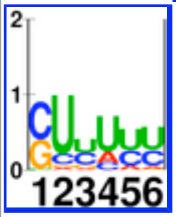	0

I1I2	0	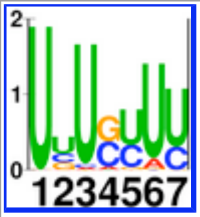	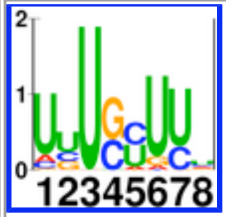
AI2_C2I2	0	0	0
C1	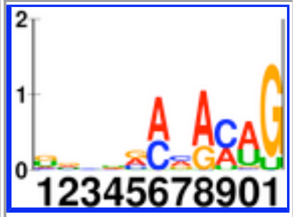	0	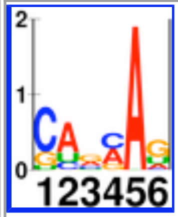
C1I1_AI1	0	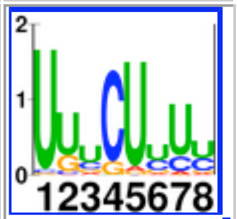 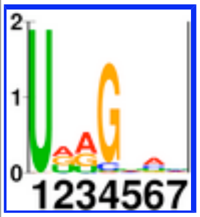	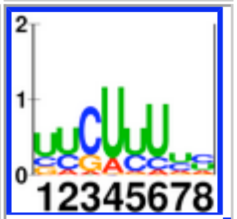 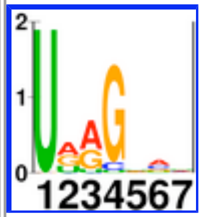
C1_A	0	0	0
C2	0	0	0
I1	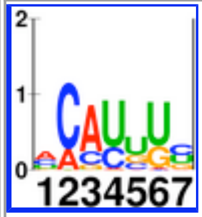	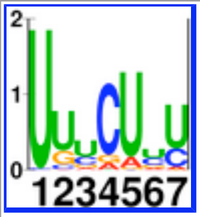	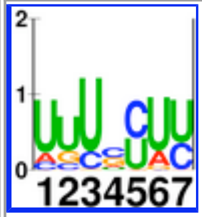
I2	0	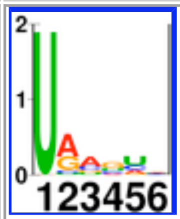	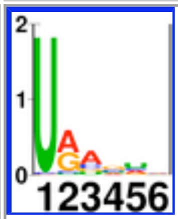
